# Design, Synthesis, and Biological Activities of Novel 1,3,5-Trimethylpyrazole-Containing Malonamide Derivatives

**DOI:** 10.3390/molecules24030562

**Published:** 2019-02-03

**Authors:** Qi-Bo Li, Min Liao, Qing Liu, Tong Feng, Zhi-Yuan Xu, Chang-Hui Rui, Shang-Zhong Liu

**Affiliations:** 1 Department of Applied Chemistry, College of Science, China Agricultural University, Beijing 100193, China; lqb@cau.edu.cn (Q.-B.L.); cygnus@cau.edu.cn (M.L.); qingqing@cau.edu.cn (Q.L.); fibber@cau.edu.cn (T.F.); xuzhiyuan@cau.edu.cn (Z.-Y.X.); 2 Key Laboratory of Integrated Pest Management in Crops, Ministry of Agriculture, Institute of Plant Protection, Chinese Academy of Agricultural Sciences, Beijing 100193, China

**Keywords:** malonamide, pyflubumide, synthesis, acaricidal and insecticidal activities

## Abstract

New 1,3,5-trimethylpyrazole-containing malonamide derivatives based on pyflubumide were designed, synthesized, and characterized using ^1^H-NMR, ^13^C-NMR, and high-resolution mass spectra (HRMS). The results of preliminary bioassays showed that the target compounds possessed good activities against *Tetranychus cinnabarinus*, *Plutella xylostella*, and *Aphis craccivora*. Most of the target compounds exhibited moderate to good acaricidal activity against *Tetranychus cinnabarinus* at a concentration of 400 µg/mL, and some showed moderate activity at a concentration of 200 µg/mL; in particular, compounds **8m** and **8p** exhibited 70.0% mortality. In addition, some of the target compounds exhibited good insecticidal activities against *Plutella xylostella* at a concentration of 200 µg/mL, especially compounds **8i** and **8o,** which achieved 100.0% mortality at a concentration of 100 µg/mL. Interestingly, some of the target compounds exhibited potent anti-aphid activity against *Aphis craccivora* at a concentration of 200 µg/mL; furthermore, compounds **8p** and **8q** demonstrated 100.0% anti-aphid activity at a concentration of 50 µg/mL. The preliminary analyses of the structure–activity relationships (SAR) indicated that the acaricidal and insecticidal activities varied significantly depending on the type of substituent and substitution pattern, which provides guidance for the further investigation of such structural modifications.

## 1. Introduction

Amide bonds are an important functional group in many of the active ingredients of agrochemicals, such as the representative amide herbicides acetochlor and metolachlor [1,2] and the succinate dehydrogenase inhibitor (SDHI) fungicides boscalid and thifluzamide [3,4,5]. The nitrogen-containing heterocycle pyrazole ring is important in the field of medicinal chemistry [6,7,8], and also endows many pesticide molecules with versatile biological activities, as exemplified by fipronil, tebufenpyrad, pyraclostrobin, and pyroxasulfone [9,10,11,12,13].

**Figure 1:**
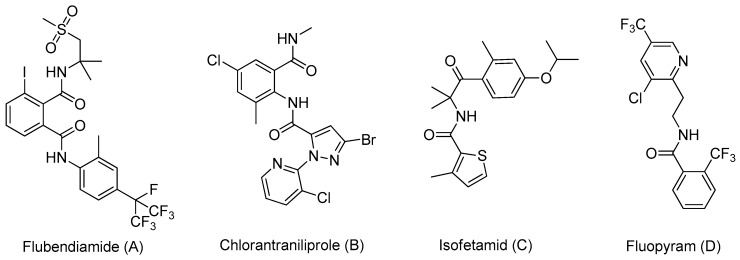
Structures of flubendiamide (**A**), chlorantraniliprole (**B**), isofetamid (**C**), and fluopyram (**D**).

**Figure 2:**
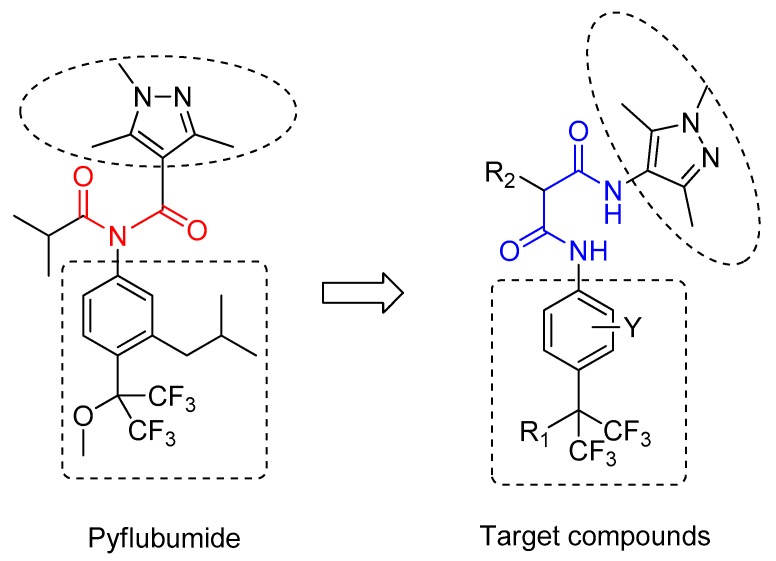
The design of the target compounds.

Due to the discovery of the excellent performance of flubendiamide **A** (Figure 1), chlorantraniliprole **B** (Figure 1), and pyflubumide (Figure 2) against pest insects and phytophagous mites in crop protection and their subsequent commercialization, the design and synthesis of new compounds containing diamide groups has become a major trend in the search for potential compounds with insecticidal and acaricidal activities [14,15].

Pyflubumide is a novel acraricide with a unique carboxanilide structure and was specifically and highly active against all stages of agriculturally important spider mites, such as two-spotted spider mite, kanzawa spider mite. and citrus red mite (LC_50_ = 1–3 µg/mL). However, it showed low activity against other pests, including *hemiptera* and *lepidoptera* (LC_50_ > 500 µg/mL). Furthermore, it is harmless to non-target arthropods, beneficial insects, and natural enemies [16,17]. It was discovered by Nihon Nohyaku Co. Ltd. when a heptafluoroisopropyl group was added to a benzene ring during the optimization of carboxamide derivatives to discover new SDHI fungicidal molecules. As SDHI fungicides, pyflubumide acts on mitochondrial complex II in the respiratory chain of phytophagous mites. Similarly, the insecticide flubendiamide, which is also based on modification of carboxamides, was also discovered and developed as the first commercial phthalic diamide insecticide by Nihon Nohyaku Co. Ltd.; however, flubendiamide targets the insect ryanodine receptor rather than mitochondrial complex II in the respiratory chain [18,19]. Subsequently, chlorantraniliprole, another insecticide that acts on the insect ryanodine receptor was developed. Chlorantraniliprole contains an anthranilic diamide skeleton and was initially discovered through converting the amide bond connection of phthalic diamides [20]. Inspired by this strategy and by the excellent acaricidal and insecticidal activities of diamide compounds, we were motivated to explore new bioactive compounds by modifying the structure of pyflubumide.

In most SDHI fungicides, two aromatic moieties are connected directly via a carboxamide. However, isofetamid **C** (Figure 1) and fluopyram **D** (Figure 1), in which two aromatic moieties are bridged by an *N*-ethyl carboxamide or *N*-oxo-ethyl carboxamide, also exhibit excellent fungicidal activity, demonstrating the possibility of discovering new compounds by modifying the connection between the two aromatic moieties [21,22].

Inspired by the design of chlorantraniliprole from flubendiamide, pyflubumide was selected as the lead compound, and a series of novel 1,3,5-trimethylpyrazole-containing malonamide derivatives were designed and synthesized (Figure 2). The malonamide skeletons of the new compounds were designed by modifying two amide bonds on one nitrogen atom, and the phenyl ring was modified with a heptafluoroisopropyl group or a methoxy-substituted hexafluoroisopropyl group to improve activity based on previous reports [23]. Moreover, all the new compounds were investigated for their acaricidal and insecticidal activities. To the best of our knowledge, it is the first report on 1,3,5-trimethylpyrazole-containing malonamide derivatives with potent acaricidal and insecticidal activities.

## 2. Results and Discussion

### 2.1. Synthesis

**Scheme 1:**
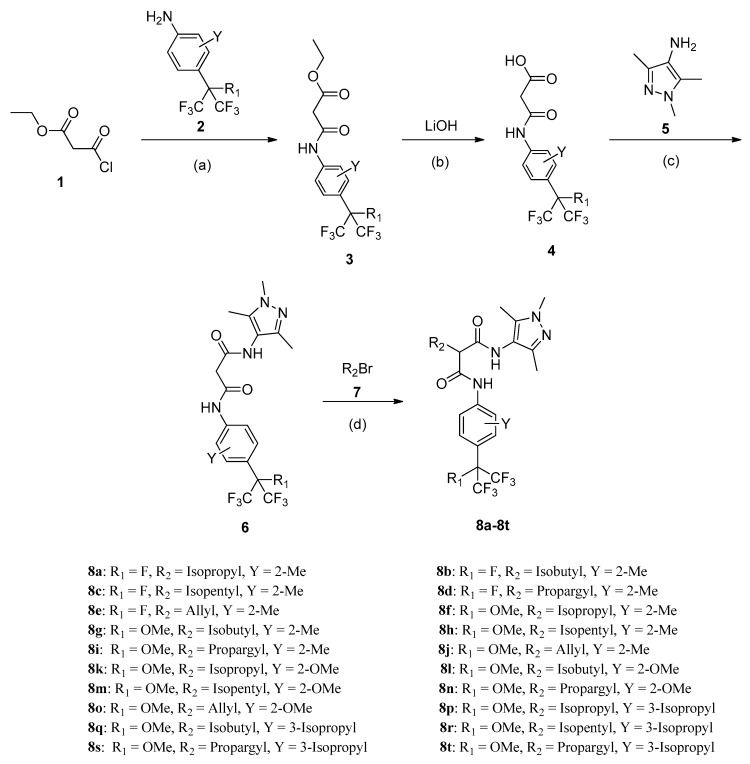
General synthetic route for compounds **8a**–**8t**. Reagents and conditions: (**a**) Et_3_N, CH_2_Cl_2_, 0–5 °C; (**b**) LiOH, H_2_O, THF, 0–25 °C; (**c**) DCC, CH_2_Cl_2_, 0–25 °C; (**d**) KTB, NMP, 0–25 °C.

The synthetic route to the target compounds **8a**~**8t** is outlined in Scheme 1; these compounds were prepared in four steps. The commercially available ethyl 3-chloro-3-oxopropanoate (**1)** was reacted with the aromatic amine derivative **2** in the presence of triethylamine to afford the intermediate **3** via a general condensation reaction in good yields. Intermediate **3** was hydrolyzed to obtain intermediate **4**, which was used in the next reaction without further purification. In the presence of the condensation agent dicyclohexylcarbodiimide (DCC), intermediate **4** was reacted with 1,3,5-trimethyl-*1H*-pyrazol-4-amine (**5**) to afford the key intermediate **6** in good yields. The last step was α-alkylation of malonamide with brominated compound **7** in the presence of potassium t-butoxide (KTB) to obtain the target compounds **8a**–**8t** [24]. Then the reaction condition of the last step was investigated to optimize the yield. The examination of the base, solvent, and temperature led to the following informative observations: (i) potassium t-butoxide was the ideal base; (ii) *N*-methyl pyrrolidone (NMP) was the suitable solvent; (iii) the product yield was highest at 0 to 5 °C. It was observed that the steric effect and electronic effect of substituents (R_2_) had a significant effect on the yield of target compounds. For example, with the isopentyl group, compound **8c** was 48.4% yield. Otherwise, compound **8e** with allyl group was 61.7% yield. The target compounds have all been structurally confirmed through ^1^H-NMR, ^13^C-NMR and high-resolution mass spectra (HRMS).

### 2.2. Biological Activity

As the primary objective of this study, preliminary tests of the acaricidal and insecticidal activity of the target compounds **8a**–**8t** against the pest species *Tetranychus cinnabarinus*, *Plutella xylostella*, and *Aphis craccivora* were carried out, as shown in Tables 1–3.

First, the acaricidal activity of the target compounds **8a**–**8t** against *Tetranychus cinnabarinus* was carried out, and the results were summarized in Table 1. Fenpyroximate was tested under the same conditions as a comparison compound. As indicated in Table 1, all new compounds exhibited certain acaricidal activity against *Tetranychus cinnabarinus*, with LC_50_ values ranging from 107.2 to 540.4 µg/mL. Among the target compounds, **8a**, **8i**–**8k**, **8m**–**8n**, and **8p**–**8r** exhibited good acaricidal activities against *Tetranychus cinnabarinus* with more than 90.0% mortality at 400 µg/mL, respectively, which were comparable to that of the control fenpyroximate (100.0%). When the concentrations of the compounds were reduced from 400 to 200 µg/mL, compounds **8m** and **8p** still showed good mortality (70.0%) against *Tetranychus cinnabarinus*. However, a great decrease of bioactivities (0–37.8%) was observed, when the concentrations were reduced from 200 to 100 µg/mL. The activities of these compounds varied drastically depending upon the types and patterns of substitution on the malonamide bridge and the phenyl ring.

For the effect of substituents R_2_ on the malonamide bridge in the series of **8a**~**8t** compounds, when the substituent R_2_ was saturated alkyl (isopropyl, isobutyl, and isopentyl), it was observed that compounds with isopropyl group, like **8a**, **8f**, **8k**, and **8p**, exhibited higher acaricidal potency than the corresponding analog with isobutyl groups, such as **8b**, **8g**, **8l**, and **8q**. Meanwhile, compared with compounds with isobutyl group, compounds with isopentyl (**8h**, **8m**, **8r**, except **8c**) revealed more activities. When the substituent R_2_ was unsaturated alkyl (propargyl and allyl), it was observed that compounds with propargyl group, like **8d**, **8i**, **8n**, and **8s**, exhibited higher acaricidal potency than the corresponding analog with allyl groups, such as **8e**, **8j**, **8o**, and **8t**. On the whole, the introduction of saturated alkyl with appropriate chain length at position R_2_ was beneficial to the acaricidal activity.

For the effect of substituents on the phenyl ring in the series of **8a**–**8t** compounds, from the activities against *Tetranychus cinnabarinus*, the compounds **8f**–**8t** with the methoxy-substituted hexafluoroisopropyl group gave increased acaricidal activity than the compounds **8a**–**8e** with the heptafluoroisopropyl group, respectively. In addition, when R_2_ was saturated alkyl, the compounds (**8k**–**8m**) with methoxy group on the 2-position of phenyl ring displayed relatively better acaricidal activity than the corresponding compounds (**8f**–**8h**) with a methyl group on the 2-position of the phenyl ring.

Next, we evaluated their insecticidal activities against *Plutella xylostella* and *Aphis craccivora,* and the results are shown in Table 2 and Table 3. Flubendiamide and imidacloprid were tested under the same conditions as a comparison compound.

As shown in Table 2, the bioactivity results indicated that most compounds exhibited certain insecticidal activities against *Plutella xylostella*, with LC_50_ values ranging from 24.2 to 569.8 µg/mL. Among the target compounds, some compounds possessed excellent insecticidal activities against *Plutella xylostella* at a concentration of 200 μg/mL. For instance, the mortalities of compounds **8g**, **8i,** and **8o** against *Plutella xylostella* were all 100.0%, respectively, which were similar to that of the control flubendiamide. When the concentration was reduced to 100 μg/mL, compounds **8i** and **8o** had 100.0% inhibition rates, and they were still active against *Plutella xylostella* even when the concentration was reduced to 50 μg/mL with inhibitory values of 83.3% and 76.7%, respectively. Based on the structure–activity data, we found that unsaturated alkyl (propargyl and allyl) on the position R_2_ were beneficial to improve the insecticidal activity of compounds, for example, compounds **8e**, **8i**, **8o**, and **8s** were the most active of the corresponding series of compounds **8a**–**8e**, **8f**–**8j**, **8k**–**8o**, and **8p**–**8t**, respectively. In addition, we found that when the substituent at 4-position of the phenyl ring was a methoxy-substituted hexafluoroisopropyl group, it was more advantageous than heptafluoroisopropyl group to increase the insecticidal activities against *Plutella xylostella*. Moreover, compared with compounds (**8p**–**8t**) with the substituent (isopropyl group) at 3-position of the phenyl ring, compounds (**8a**–**8o**) with the substituent (methyl and methoxy groups) at 2-position of phenyl ring showed more advantages in insecticidal activity.

As shown in Table 3, the bioactivity results indicated that all compounds exhibited certain insecticidal activities against *Aphis craccivora*, with LC_50_ values ranging from 8.1 to 225.3 µg/mL. As can be seen, some target compounds demonstrated excellent insecticidal activities against *Aphis craccivora* at a concentration of 100 μg/mL, for instance, compounds **8a**, **8b**, **8c**, **8j**, **8k**, **8p**, and **8q** all had over 90.0% inhibition rates, which were comparable to that of the control imidacloprid (100.0%). When the concentration was reduced to 50 µg/mL, the mortalities of compounds **8a**, **8k**, **8p**, and **8q** were 80.0%, 76.7%, 100.0%, and 100.0%, respectively. Even if the concentration was reduced to 20 µg/mL, compounds **8p** and **8q** were still active against *Aphis craccivora* with the mortalities of 88.3% and 83.3%, respectively. Based on the structure–activity data, we found that the saturated alkyl on the position R_2_ was more helpful to increase the mortality activities against *Aphis craccivora* than the unsaturated alkyl. Especially, when R_2_ substituent group was an isopropyl group, it showed better insecticidal activities against *Aphis craccivora* than other types of R_2_ substituents. In addition, compared with compounds (**8a**–**8e**) with the substituent heptafluoroisopropyl group at 4-position of the phenyl ring, compounds (**8f**–**8t**) with the substituent methoxy-substituted hexafluoroisopropyl group showed more advantages in insecticidal activity. Moreover, we found that the introduction of isopropyl group at 3-position of the phenyl ring influenced the insecticidal activities greatly, for example, compounds **8p** and **8q** were a more effective insecticidal agent than other compounds.

## 3. Experimental

### 3.1. Chemicals and Instrumentation

Nuclear magnetic resonance spectra were recorded using a Bruker Avance DPX300 (San Jose, CA, USA) spectrometer in CDCl_3_ or DMSO-d_6_ solution (^1^H-NMR at 300 MHz and ^13^C-NMR at 75 MHz). Chemical shift values (δ) are given in parts per million with tetramethylsilane as the internal standard. The melting points of all compounds were determined using a B-III microscope (Beijing Technical Instrument Co., Beijing, China) and are uncorrected. High-resolution mass spectra (HRMS) were obtained using a Thermo Scientific Q Exactive instrument. Analytical TLC was performed on silica gel GF_254_ (200–300 mesh). Column chromatographic purification was carried out using silica gel.

Reagents and solvents were obtained from Beijing Chemical Reagents Co. (Beijing, China) and were used without purification. As shown in Scheme 1, ethyl malonyl chloride (compound **1**), 1,3,5-trimethyl-*1H*-pyrazol-4-amine (compound **5**)**,** and compound **7** were purchased from Shanghai Aladdin Bio-Chem Technology Co., LTD. Compound **2** was prepared according to literature methods without purification [25,26].

### 3.2. Synthesis Procedures

#### 3.2.1. General synthesis Procedure for Intermediate **3**

A solution of intermediate **2** (60 mmol) and triethylamine (60 mmol) dissolved in dichloromethane (100 mL) was cooled to 0–5 °C. Ethyl malonyl chloride (60 mmol) was added dropwise over ten minutes, and the mixture was stirred for a further thirty minutes at this temperature. The reaction mixture was then stirred at room temperature until the reaction was complete as indicated by TLC. At the end of the reaction, the reaction mixture was successively washed with diluted hydrochloric acid (30 mL, 5% vol) and brine, dried over anhydrous sodium sulfate, and concentrated in vacuo. The residue was purified by flash chromatography on silica gel using petroleum ether (60–90 °C) and ethyl acetate (*v*/*v* = 15:1) as the eluent to give intermediate **3**, with yields ranging from 89.4% to 93.5%. Physical, spectral data, and ^1^H-NMR spectra of intermediate **3** were provided in the Supplementary Materials.

#### 3.2.2. General Synthesis Procedure for Intermediate **4**

To a solution of intermediate **3** (20 mmol) in tetrahydrofuran (50 mL) was added LiOH (2 mol/L, 60 mL) dropwise at 0 °C, and the reaction mixture was then warmed to room temperature and stirred overnight until the reaction was completed as indicated by TLC. The solvent was vacuum-evaporated, the residue was acidified with diluted hydrochloric acid (2 M, 20 mL), and the aqueous layer was extracted with dichloromethane (30 mL × 3). The combined organic phase was dried over anhydrous sodium sulfate and then concentrated in vacuo to give intermediate **4**, which was used in the next reaction without further purification.

#### 3.2.3. General Synthesis Procedure for Intermediate **6**

To a mixture of intermediate **4** (15 mmol) and 1,3,5-trimethyl-*1H*-pyrazol-4-amine (15 mmol) in dichloromethane (50 mL) was added dicyclohexylcarbodiimide (DCC, 22.5 mmol) in dichloromethane (20 mL) dropwise at 0 °C. The reaction mixture was then warmed to room temperature and stirred overnight until the reaction was completed. The mixture was then filtered, and the filtrate was washed successively with saturated sodium bicarbonate solution and saturated brine, dried over anhydrous sodium sulfate, and concentrated in vacuo. The residue was purified by flash chromatography on silica gel using petroleum ether (60−90 °C) and ethyl acetate (*v/v* = 1:1) as the eluent to give intermediate **6**, with yields ranging from 82.5% to 86.7%. Physical, spectral data, and ^1^H-NMR spectra of intermediate **6** were provided in the Supplementary Materials.

#### 3.2.4. General Synthesis Procedure for the Target Compounds **8a**–**8t**

To a solution of intermediate **6** (4 mmol) in *N*-methyl pyrrolidone (NMP) was added potassium t-butoxide (KTB, 4.4 mmol) in portions at 0 °C. After 30 min, intermediate **7** (4.4 mmol) was added at 0 °C. The mixture was stirred 6 h at room temperature and then poured into ice-water and extracted with ethyl acetate (30 mL × 3). The combined organic phase was washed with saturated brine (30 mL × 3), dried over anhydrous sodium sulfate, and concentrated in vacuo. The residue was purified by flash chromatography on silica gel using petroleum ether (60−90 °C) and ethyl acetate (*v*/*v* = 1:3) as the eluent to give the target compounds **8a**~**8t**, with yields ranging from 46.7% to 62.5%. All twenty 1,3,5-trimethylpyrazole-containing malonamide derivatives **8a**~**8t** were novel, and their physical and spectral data are listed below. ^1^H-NMR, ^13^C-NMR, and HRMS spectra are provided in the Supplementary Materials.

*2-Isopropyl-N^1^-(2-methyl-4-(perfluoropropan-2-yl)phenyl)-N^3^-(1,3,5-trimethyl-1H-pyrazol-4-yl)malonamide* (**8a**): White solid; m.p. 197–199 °C; yield 53.2%; ^1^H-NMR (300 MHz, Chloroform-*d*): δ 9.54 (s, 1H, pyrazole-NH), 8.51 (s, 1H, Ar-NH), 8.14 (d, *J* = 9.3 Hz, 1H, Ar-H), 7.43-7.41 (m, 2H, Ar-H), 3.69 (s, 3H, N-CH_3_), 3.19 (d, *J* = 9.7 Hz, 1H, O=C-CH-C=O), 2.47-2.39 (m, 1H, CH(CH_3_)_2_), 2.35 (s, 3H, Ar-CH_3_), 2.11 (d, *J* = 1.2 Hz, 6H, pyrazole-CH_3_), 1.13 (dd, *J* = 9.5, 6.7 Hz, 6H, CH(CH_3_)_2_). ^13^C-NMR (75 MHz, Chloroform-*d*): δ 170.69 (C=O), 168.38 (C=O), 142.68 (C=N, pyrazole-C_3_), 137.95 (C-N-N, pyrazole-C_5_), 134.47 (C-N, Ar-C_1_), 128.67 (d, *J*_CF_ = 2.3 Hz, Ar-C_2_), 127.34 (d, *J*_CF_ = 11.3 Hz, Ar-C_3_), 123.81 (d, *J*_CF_ = 10.5 Hz, Ar-C_5_), 122.15 (d, *J*_CF_ = 20.3 Hz, Ar-C_4_), 121.25 (d, *J*_CF_ = 1.5 Hz, Ar-C_6_), 120.22 (qd, ^1^*J*_CF_ = 284.3 Hz, ^2^*J*_CF_ = 27.8 Hz, CF_3_), 113.67 (C-N, pyrazole-C_4_), 90.98 (dsept, ^1^*J*_CF_ = 200.3 Hz, ^2^*J*_CF_ = 33.0 Hz, CF(CF_3_)_2_), 62.71 (O=C-C-C=O), 35.90 (N-C, pyrazole-CH_3_), 32.72 (CH(CH_3_)_2_), 20.53 (CH(CH_3_)_2_), 20.26 (CH(CH_3_)_2_), 17.69 (Ar-CH_3_), 11.00 (pyrazole-CH_3_), 9.15 (pyrazole-CH_3_). HRMS calcd. for C_22_H_25_F_7_N_4_O_2_ ([M+H]^+^), 511.1939; found, 511.1931.

*2-Isobutyl-N^1^-(2-methyl-4-(perfluoropropan-2-yl)phenyl)-N^3^-(1,3,5-trimethyl-1H-pyrazol-4-yl)malonamide* (**8b**): White solid; m.p. 204–206 °C; yield 56.5%; ^1^H-NMR (300 MHz, Chloroform-*d*): δ 9.48 (s, 1H, pyrazole-NH), 8.41 (s, 1H, Ar-NH), 8.11 (d, *J* = 9.3 Hz, 1H, Ar-H), 7.43-7.41 (m, 2H, Ar-H), 3.69 (s, 3H, N-CH_3_), 3.63 (t, *J* = 7.6 Hz, 1H, O=C-CH-C=O), 2.35 (s, 3H, Ar-CH_3_), 2.10 (d, *J* = 1.9 Hz, 6H, pyrazole-CH_3_), 2.01-1.87 (m, 2H, O=C-CH-CH_2_), 1.80-1.71 (m, 1H, CH(CH_3_)_2_), 1.00 (d, *J* = 6.5 Hz, 6H, CH(CH_3_)_2_). ^13^C-NMR (75 MHz, Chloroform-*d*): δ 171.19 (C=O), 169.13 (C=O), 142.64 (C=N, pyrazole-C_3_), 137.89 (C-N-N, pyrazole-C_5_), 134.42 (C-N, Ar-C_1_), 128.81 (d, *J*_CF_ = 2.3 Hz, Ar-C_2_), 127.34 (d, *J*_CF_ = 11.3 Hz, Ar-C_3_), 123.79 (d, *J*_CF_ = 10.5 Hz, Ar-C_5_), 122.28 (d, *J*_CF_ = 20.3 Hz, Ar-C_4_), 121.43 (d, *J*_CF_ = 0.8 Hz, Ar-C_6_), 120.22 (qd, ^1^*J*_CF_ = 284.3 Hz, ^2^*J*_CF_ = 27.8 Hz, CF_3_), 113.60 (C-N, pyrazole-C_4_), 90.97 (dsept, ^1^*J*_CF_ = 200.3 Hz, ^2^*J*_CF_ = 33.0 Hz, CF(CF_3_)_2_), 53.53 (O=C-C-C=O), 42.50 (O=C-CH-CH_2_), 35.89 (N-C, pyrazole-CH_3_), 26.17 (CH(CH_3_)_2_), 22.11 (CH(CH_3_)_2_), 21.69 (CH(CH_3_)_2_), 17.66 (Ar-CH_3_), 10.88 (pyrazole-CH_3_), 9.09 (pyrazole-CH_3_). HRMS calcd. for C_23_H_27_F_7_N_4_O_2_ ([M+H]^+^), 525.2095; found, 525.2086.

*2-Isopentyl-N^1^-(2-methyl-4-(perfluoropropan-2-yl)phenyl)-N^3^-(1,3,5-trimethyl-1H-pyrazol-4-yl)malonamide* (**8c**): Light yellow solid; m.p. 200–202 °C; yield 48.4%; ^1^H-NMR (300 MHz, Chloroform-*d*): δ 9.57 (s, 1H, pyrazole-NH), 8.20 (s, 1H, Ar-NH), 8.16 (d, *J* = 9.3 Hz, 1H, Ar-H), 7.44–7.42 (m, 2H, Ar-H), 3.70 (s, 3H, N-CH_3_), 3.43 (t, *J* = 7.5 Hz, 1H, O=C-CH-C=O), 2.36 (s, 3H, Ar-CH_3_), 2.12 (s, 6H, pyrazole-CH_3_), 2.09-2.03 (m, 2H, O=C-CH-CH_2_), 1.66–1.57 (m, 1H, CH(CH_3_)_2_), 1.41–1.33 (m, 2H, CH_2_-CH(CH_3_)_2_), 0.89 (d, *J* = 6.6 Hz, 6H, CH(CH_3_)_2_). ^13^C-NMR (75 MHz, Chloroform-*d*): δ 171.29 (C=O), 168.90 (C=O), 142.66 (C=N, pyrazole-C_3_), 137.93 (C-N-N, pyrazole-C_5_), 134.48 (C-N, Ar-C_1_), 128.70 (d, *J*_CF_ = 2.3 Hz, Ar-C_2_), 127.33 (d, *J*_CF_ = 10.5 Hz, Ar-C_3_), 123.82 (d, *J*_CF_ = 10.5 Hz, Ar-C_5_), 122.21 (d, *J*_CF_ = 20.3 Hz, Ar-C_4_), 121.34 (d, *J*_CF_ = 1.5 Hz, Ar-C_6_), 120.22 (qd, ^1^*J*_CF_ = 285 Hz, ^2^*J*_CF_ = 28.5 Hz, CF_3_), 113.55 (C-N, pyrazole-C_4_), 90.98 (dsept, ^1^*J*_CF_ = 200.3 Hz, ^2^*J*_CF_ = 33.0 Hz, CF(CF_3_)_2_), 55.15 (O=C-C-C=O), 36.31 (N-C, pyrazole-CH_3_), 35.91 (CH_2_-CH(CH_3_)_2_), 31.64 (CH(CH_3_)_2_), 27.39 (O=C-CH-CH_2_), 21.97 (CH(CH_3_)_2_), 21.89 (CH(CH_3_)_2_), 17.72 (Ar-CH_3_), 10.93 (pyrazole-CH_3_), 9.13 (pyrazole-CH_3_). HRMS calcd. for C_24_H_29_F_7_N_4_O_2_ ([M+H]^+^), 539.2252; found, 539.2244.

*N^1^-(2-Methyl-4-(perfluoropropan-2-yl)phenyl)-2-(prop-2-yn-1-yl)-N^3^-(1,3,5-trimethyl-1H-pyrazol-4-yl)malonamide* (**8d**): White solid; m.p. 83–85 °C; yield 50.2%; ^1^H-NMR (300 MHz, DMSO-*d*_6_): δ 9.70 (s, 1H, pyrazole-NH), 9.25 (s, 1H, Ar-NH), 7.83 (d, *J* = 8.3 Hz, 1H, Ar-H), 7.52-7.49 (m, 2H, Ar-H), 3.80 (t, *J* = 7.5 Hz, 1H, O=C-CH-C=O), 3.62 (s, 3H, N-CH_3_), 2.94 (t, *J* = 2.5 Hz, 1H, C≡CH), 2.84–2.80 (m, 2H, CH_2_-C≡CH), 2.33 (s, 3H, Ar-CH_3_), 2.05 (s, 3H, pyrazole-CH_3_), 1.96 (s, 3H, pyrazole-CH_3_). ^13^C-NMR (75 MHz, DMSO-*d*_6_): δ 167.39 (C=O), 166.93 (C=O), 141.82 (C=N, pyrazole-C_3_), 139.32 (C-N-N, pyrazole-C_5_), 134.23 (C-N, Ar-C_1_), 132.07 (d, *J*_CF_ = 2.3 Hz, Ar-C_2_), 127.24 (d, *J*_CF_ = 9.8 Hz, Ar-C_3_), 124.42 (d, *J*_CF_ = 0.8 Hz, Ar-C_6_), 123.52 (d, *J*_CF_ = 9.8 Hz, Ar-C_5_), 122.23 (d, *J*_CF_ = 21 Hz, Ar-C_4_) 120.42 (qd, ^1^*J*_CF_ = 284.3 Hz, ^2^*J*_CF_ = 27.8 Hz, CF_3_), 114.80 (C-N, pyrazole-C_4_), 91.24 (dsept, ^1^*J*_CF_ = 200.3 Hz, ^2^*J*_CF_ = 33.0 Hz, CF(CF_3_)_2_), 81.57 (C≡CH), 72.83 (C≡CH), 52.45 (O=C-C-C=O), 36.08 (N-C, pyrazole-CH_3_), 19.33 (CH_2_-C≡CH), 17.90 (Ar-CH_3_), 11.22 (pyrazole-CH_3_), 9.10 (pyrazole-CH_3_). HRMS calcd. for C_22_H_21_F_7_N_4_O_2_ ([M+H]^+^), 507.1625; found, 507.1619.

*2-Allyl-N^1^-(2-methyl-4-(perfluoropropan-2-yl)phenyl)-N^3^-(1,3,5-trimethyl-1H-pyrazol-4-yl)malonamide* (**8e**): Light yellow solid; m.p. 155–157 °C; yield 61.7%; ^1^H-NMR (300 MHz, Chloroform-*d*): δ 9.67 (s, 1H, pyrazole-NH), 8.38 (s, 1H, Ar-NH), 8.13 (d, *J* = 9.3 Hz, 1H, Ar-H), 7.43–7.41 (m, 2H, Ar-H), 5.96–5.78 (m, 1H, CH=CH_2_), 5.27–5.12 (m, 2H, CH=CH_2_), 3.68 (s, 3H, N-CH_3_), 3.54 (t, *J* = 7.4 Hz, 1H, O=C-CH-C=O), 3.42–3.33 (m, 1H, O=C-CH-CH_2_), 2.83 (s, 3H, Ar-CH_3_), 2.35 (s, 3H, pyrazole-CH_3_), 2.09 (s, 3H, pyrazole-CH_3_), 2.04–1.99 (m, 1H, O=C-CH-CH_2_). ^13^C-NMR (75 MHz, Chloroform-*d*): δ 170.56 (C=O), 168.14 (C=O), 142.68 (C=N, pyrazole-C_3_), 137.83 (C-N-N, pyrazole-C_5_), 134.58 (C-N, Ar-C_1_), 132.98 (CH=CH_2_), 128.94 (d, *J*_CF_ = 1.5 Hz, Ar-C_2_), 127.33 (d, *J*_CF_ = 10.5 Hz, Ar-C_3_), 123.78 (d, *J*_CF_ = 10.5 Hz, Ar-C_5_), 122.33 (d, *J*_CF_ = 20.3 Hz, Ar-C_4_), 121.53 (d, *J*_CF_ = 1.5 Hz, Ar-C_6_), 115.71 (qd, ^1^*J*_CF_ = 284.3 Hz, ^2^*J*_CF_ = 27.0 Hz, CF_3_), 118.36 (CH=CH_2_), 113.51 (C-N, pyrazole-C_4_), 90.96 (dsept, ^1^*J*_CF_ = 200.3 Hz, ^2^*J*_CF_ = 33.0 Hz, CF(CF_3_)_2_), 54.39 (O=C-C-C=O), 37.41 (N-C, pyrazole-CH_3_), 35.86(O=C-CH-CH_2_), 17.71 (Ar-CH_3_), 10.90 (pyrazole-CH_3_), 9.08 (pyrazole-CH_3_). HRMS calcd. for C_22_H_23_F_7_N_4_O_2_ ([M+H]^+^), 509.1782; found, 509.1777.

*N^1^-(4-(1,1,1,3,3,3-Hexafluoro-2-methoxypropan-2-yl)-2-methylphenyl)-2-isopropyl-N^3^-(1,3,5-trimethyl-1H-pyrazol-4-yl)malonamide* (**8f**): Light yellow solid; m.p. 192–193 °C; yield 58.4%; ^1^H-NMR (300 MHz, Chloroform-*d*): δ 9.50 (s, 1H, pyrazole-NH), 8.59 (s, 1H, Ar-NH), 8.09 (d, *J* = 8.8 Hz, 1H, Ar-H), 7.40–7.37 (m, 2H, Ar-H), 3.68 (s, 3H, N-CH_3_), 3.47 (s, 3H, OCH_3_), 3.20 (d, *J* = 9.7 Hz, 1H, O=C-CH-C=O), 2.47–2.40 (m, 1H, CH(CH_3_)_2_), 2.35 (s, 3H, Ar-CH_3_), 2.11 (s, 6H, pyrazole-CH_3_), 1.13 (dd, *J* = 8.5, 6.7 Hz, 6H, CH(CH_3_)_2_). ^13^C-NMR (75 MHz, Chloroform-*d*): δ 170.71 (C=O), 168.39 (C=O), 142.68 (C=N, pyrazole-C_3_), 137.20 (C-N-N, pyrazole-C_5_), 134.48 (C-N, Ar-C_1_), 129.81 (Ar-C_2_), 128.54 (Ar-C_3_), 126.30 (Ar-C_5_), 123.36 (Ar-C_4_), 122.06 (q, *J*_CF_ = 288.8 Hz, CF_3_), 121.26 (Ar-C_6_), 113.73 (C-N, pyrazole-C_4_), 82.38 (hept, *J*_CF_ = 28.5 Hz, C(CF_3_)_2_), 62.65 (O=C-C-C=O), 53.83 (OCH_3_), 35.89 (N-C, pyrazole-CH_3_), 32.70 (CH(CH_3_)_2_), 20.52 (CH(CH_3_)_2_), 20.27 (CH(CH_3_)_2_), 17.78 (Ar-CH_3_), 10.99 (pyrazole-CH_3_), 9.15 (pyrazole-CH_3_). HRMS calcd. for C_23_H_28_F_6_N_4_O_3_ ([M+H]^+^), 523.2138; found, 523.2132.

*N^1^-(4-(1,1,1,3,3,3-Hexafluoro-2-methoxypropan-2-yl)-2-methylphenyl)-2-isobutyl-N^3^-(1,3,5-trimethyl-1H-pyrazol-4-yl)malonamide* (**8g**): White solid; m.p. 204–205 °C; yield 51.5%; ^1^H-NMR (300 MHz, Chloroform-*d*): δ 9.43 (s, 1H, pyrazole-NH), 8.47 (s, 1H, Ar-NH), 8.06 (d, *J* = 9.2 Hz, 1H, Ar-H), 7.40–7.38 (m, 2H, Ar-H), 3.69 (s, 3H, N-CH_3_), 3.64 (t, *J* = 7.5 Hz, 1H, O=C-CH-C=O), 3.47 (s, 3H, OCH_3_), 2.35 (s, 3H, Ar-CH_3_), 2.10 (s, 6H, pyrazole-CH_3_), 2.03–1.88 (m, 2H, O=C-CH-CH_2_), 1.82–1.69 (m, 1H, CH(CH_3_)_2_), 1.00 (d, *J* = 6.5 Hz, 6H, CH(CH_3_)_2_). ^13^C-NMR (75 MHz, Chloroform-*d*): δ 171.20 (C=O), 169.09 (C=O), 142.65 (C=N, pyrazole-C_3_), 137.13 (C-N-N, pyrazole-C_5_), 134.44 (C-N, Ar-C_1_), 129.80 (Ar-C_2_), 128.63 (Ar-C_3_), 126.30 (Ar-C_5_), 123.49 (Ar-C_4_), 122.06 (q, *J*_CF_ = 288.8 Hz, CF_3_), 121.39 (Ar-C_6_), 113.63 (C-N, pyrazole-C_4_), 82.38 (hept, *J*_CF_ = 28.5 Hz, C(CF_3_)_2_), 53.84 (OCH_3_), 53.53 (O=C-C-C=O), 42.51 (O=C-CH-CH_2_), 35.90 (N-C, pyrazole-CH_3_), 26.18 (CH(CH_3_)_2_), 22.12 (CH(CH_3_)_2_), 21.73 (CH(CH_3_)_2_), 17.75 (Ar-CH_3_), 10.89 (pyrazole-CH_3_), 9.11 (pyrazole-CH_3_). HRMS calcd. for C_24_H_30_F_6_N_4_O_3_ ([M+H]^+^), 537.2295; found, 537.2288.

*N^1^-(4-(1,1,1,3,3,3-Hexafluoro-2-methoxypropan-2-yl)-2-methylphenyl)-2-isopentyl-N^3^-(1,3,5-trimethyl-1H-pyrazol-4-yl)malonamide* (**8h**): White solid; m.p. 186–187 °C; yield 56.1%; ^1^H-NMR (300 MHz, Chloroform-*d*): δ 9.55 (s, 1H, pyrazole-NH), 8.40(s, 1H, Ar-NH), 8.10 (d, *J* = 8.4 Hz, 1H, Ar-H), 7.41–7.38 (m, 2H, Ar-H), 3.69 (s, 3H, N-CH_3_), 3.47 (s, 3H, OCH_3_), 3.45 (t, *J* = 6.0 Hz, 1H, O=C-CH-C=O), 2.35 (s, 3H, Ar-CH_3_), 2.12 (s, 6H, pyrazole-CH_3_), 2.10–2.03 (m, 2H, O=C-CH-CH_2_), 1.65–1.56 (m, 1H, CH(CH_3_)_2_), 1.41–1.33 (m, 2H, CH_2_-CH(CH_3_)_2_), 0.88 (d, *J* = 6.6 Hz, 6H, CH(CH_3_)_2_). ^13^C-NMR (75 MHz, Chloroform-*d*): δ 171.30 (C=O), 169.01 (C=O), 142.63 (C=N, pyrazole-C_3_), 137.17 (C-N-N, pyrazole-C_5_), 134.45 (C-N, Ar-C_1_), 129.81 (Ar-C_2_), 128.64 (Ar-C_3_), 126.28 (Ar-C_5_), 123.45 (Ar-C_4_), 122.06 (q, *J*_CF_ = 288.8 Hz, CF_3_), 121.38 (Ar-C_6_), 113.68 (C-N, pyrazole-C_4_), 82.38 (hept, *J*_CF_ = 28.5 Hz, C(CF_3_)_2_), 55.06 (O=C-C-C=O), 53.81 (OCH_3_), 36.30 (N-C, pyrazole-CH_3_), 35.86 (CH_2_-CH(CH_3_)_2_), 31.62 (CH(CH_3_)_2_), 27.37 (O=C-CH-CH_2_), 21.96 (CH(CH_3_)_2_), 21.88 (CH(CH_3_)_2_), 17.78 (Ar-CH_3_), 10.92 (pyrazole-CH_3_), 9.10 (pyrazole-CH_3_). HRMS calcd. for C_25_H_32_F_6_N_4_O_3_ ([M+H]^+^), 551.2451; found, 551.2446.

*N^1^-(4-(1,1,1,3,3,3-Hexafluoro-2-methoxypropan-2-yl)-2-methylphenyl)-2-(prop-2-yn-1-yl)-N^3^-(1,3,5-trimethyl-1H-pyrazol-4-yl)malonamide* (**8i**): Light yellow solid; m.p. 54–55 °C; yield 46.7%; ^1^H-NMR (300 MHz, Chloroform-d): δ 9.61 (s, 1H, pyrazole-NH), 8.48 (s, 1H, Ar-NH), 8.05 (d, *J* = 8.5 Hz, 1H, Ar-H), 7.40-7.37 (m, 2H, Ar-H), 3.69-3.64 (m, 4H, O=C-CH-C=O and N-CH_3_), 3.46 (s, 3H, OCH_3_), 2.97 (dd, *J* = 7.5, 2.5 Hz, 2H, CH_2_-C≡CH), 2.33 (s, 3H, Ar-CH_3_), 2.15 (t, *J* = 2.6 Hz, 1H, C≡CH), 2.11 (d, *J* = 2.9 Hz, 6H, pyrazole-CH_3_). ^13^C-NMR (75 MHz, Chloroform-d): δ 169.54 (C=O), 166.75 (C=O), 142.79 (C=N, pyrazole-C_3_), 136.94 (C-N-N, pyrazole-C_5_), 134.78 (C-N, Ar-C_1_), 129.80 (Ar-C_2_), 129.09 (Ar-C_3_), 126.27 (Ar-C_5_), 123.77 (Ar-C_4_), 122.04 (q, *J*_CF_ = 288.8 Hz, CF_3_), 121.79 (Ar-C_6_), 113.47 (C-N, pyrazole-C_4_), 82.36 (hept, *J*_CF_ = 28.5 Hz, C(CF_3_)_2_), 79.41 (C≡CH), 71.64 (C≡CH), 53.83 (O=C-C-C=O), 52.84 (OCH_3_), 35.83 (N-C, pyrazole-CH_3_), 22.06 (CH_2_-C≡CH), 17.78 (Ar-CH_3_), 10.86 (pyrazole-CH_3_), 9.03 (pyrazole-CH_3_). HRMS calcd. for C_23_H_24_F_6_N_4_O_3_ ([M+H]^+^), 519.1825; found, 519.1818.

*2-Allyl-N^1^-(4-(1,1,1,3,3,3-hexafluoro-2-methoxypropan-2-yl)-2-methylphenyl)-N^3^-(1,3,5-trimethyl-1H-pyrazol-4-yl)malonamide* (**8j**): White solid; m.p. 146–148 °C; yield 62.5%; ^1^H-NMR (300 MHz, Chloroform-*d*): δ 9.56 (s, 1H, pyrazole-NH), 8.30 (s, 1H, Ar-NH), 8.07 (d, *J* = 8.7 Hz, 1H, Ar-H), 7.40–7.38 (m, 2H, Ar-H), 5.93–5.79 (m, 1H, CH=CH_2_), 5.28–5.12 (m, 2H, CH=CH_2_), 3.68 (s, 3H, N-CH_3_), 3.55 (t, *J* = 7.4 Hz, 1H, O=C-CH-C=O), 3.47 (s, 3H, OCH_3_), 2.82 (t, *J* = 7.2 Hz, 2H, O=C-CH-CH_2_), 2.34 (s, 3H, Ar-CH_3_), 2.09 (d, *J* = 1.4 Hz, 6H, pyrazole-CH_3_). ^13^C-NMR (75 MHz, Chloroform-*d*): δ 170.56 (C=O), 168.07 (C=O), 142.71 (C=N, pyrazole-C_3_), 137.08 (C-N-N, pyrazole-C_5_), 134.61 (C-N, Ar-C_1_), 133.04 (CH=CH_2_), 129.80 (Ar-C_2_), 128.73 (Ar-C_3_), 126.31 (Ar-C_5_), 123.54 (Ar-C_4_), 122.05 (q, *J*_CF_ = 288.8 Hz, CF_3_), 121.49 (Ar-C_6_), 118.35 (CH=CH_2_), 113.50 (C-N, pyrazole-C_4_), 82.38 (hept, *J*_CF_ = 28.5 Hz, C(CF_3_)_2_), 54.41 (O=C-C-C=O), 53.84 (OCH_3_), 37.40 (N-C, pyrazole-CH_3_), 35.88 (O=C-CH-CH_2_), 17.81 (Ar-CH_3_), 10.90 (pyrazole-CH_3_), 9.11 (pyrazole-CH_3_). HRMS calcd. for C_23_H_26_F_6_N_4_O_3_ ([M + H]^+^), 521.1982; found, 521.1976.

*N^1^-(4-(1,1,1,3,3,3-Hexafluoro-2-methoxypropan-2-yl)-2-methoxyphenyl)-2-isopropyl-N^3^-(1,3,5-trimethyl-1H-pyrazol-4-yl)malonamide* (**8k**): Light yellow solid; m.p. 74–76 °C; yield 58.3%; ^1^H-NMR (300 MHz, Chloroform-d): δ 9.39 (s, 1H, pyrazole-NH), 8.42 (d, *J* = 8.6 Hz, 1H, Ar-H), 8.37 (s, 1H, Ar-NH), 7.16 (d, *J* = 8.6 Hz, 1H, Ar-H), 7.05 (s, 1H, Ar-H), 3.91 (s, 3H, Ar-OCH_3_), 3.68 (s, 3H, N-CH_3_), 3.49 (s, 3H, OCH_3_), 3.09 (d, *J* = 9.9 Hz, 1H, O=C-CH-C=O), 2.51–2.40 (m, 1H, CH(CH_3_)_2_), 2.11 (s, 6H, pyrazole-CH_3_), 1.11 (dd, *J* = 16.9, 6.6 Hz, 6H, CH(CH_3_)_2_). ^13^C-NMR (75 MHz, Chloroform-d): δ 169.60 (C=O), 168.48 (C=O), 148.25 (Ar-C_2_), 142.72 (C=N, pyrazole-C_3_), 134.46 (C-N, Ar-C_1_), 128.74 (C-N-N, pyrazole-C_5_), 122.90 (Ar-C_4_), 122.05 (q, *J*_CF_ = 288.8 Hz, CF_3_), 120.76 (Ar-C_5_), 119.24 (Ar-C_6_), 113.84 (C-N, pyrazole-C_4_), 109.51 (Ar-C_3_), 82.45 (hept, *J*_CF_ = 28.5 Hz, C(CF_3_)_2_), 63.58 (O=C-C-C=O), 55.64 (Ar-OCH_3_), 53.90 (OCH_3_), 35.87 (N-C, pyrazole-CH_3_), 32.35 (CH(CH_3_)_2_), 20.45 (CH(CH_3_)_2_), 20.24 (CH(CH_3_)_2_), 10.95 (pyrazole-CH_3_), 9.19 (pyrazole-CH_3_). HRMS calcd. for C_23_H_28_F_6_N_4_O_4_ ([M+H]^+^), 539.2088; found, 539.2081.

*N^1^-(4-(1,1,1,3,3,3-Hexafluoro-2-methoxypropan-2-yl)-2-methoxyphenyl)-2-isobutyl-N^3^-(1,3,5-trimethyl-1H-pyrazol-4-yl)malonamide* (**8l**): White solid; m.p. 193–195 °C; yield 51.8%; ^1^H-NMR (300 MHz, Chloroform-d): δ 9.32 (s, 1H, pyrazole-NH), 8.41 (d, *J* = 8.6 Hz, 1H, Ar-H), 8.29 (s, 1H, Ar-NH), 7.16 (d, *J* = 8.6 Hz, 1H, Ar-H), 7.05 (s, 1H, Ar-H), 3.92 (s, 3H, Ar-OCH_3_), 3.68 (s, 3H, N-CH_3_), 3.55 (t, *J* = 7.6 Hz, 1H, O=C-CH-C=O), 3.49 (s, 3H, OCH_3_), 2.10 (d, *J* = 1.1 Hz, 6H, pyrazole-CH_3_), 2.04–1.84 (m, 2H, O=C-CH-CH_2_), 1.80-1.67 (m, 1H, CH(CH_3_)_2_), 0.99 (d, *J* = 6.5 Hz, 6H, CH(CH_3_)_2_). ^13^C-NMR (75 MHz, Chloroform-d): δ 170.02 (C=O), 169.05 (C=O), 148.18 (Ar-C_2_), 142.72 (C=N, pyrazole-C_3_), 134.47 (C-N, Ar-C_1_), 128.70 (C-N-N, pyrazole-C_5_), 122.98 (Ar-C_4_), 120.78 (Ar-C_5_), 122.04 (q, *J*_CF_ = 288.8 Hz, CF_3_), 119.27 (Ar-C_6_), 113.72 (C-N, pyrazole-C_4_), 109.46 (Ar-C_3_), 82.45 (hept, *J*_CF_ = 28.5 Hz, C(CF_3_)_2_), 55.64 (Ar-OCH_3_), 54.35 (OCH_3_), 53.92 (O=C-C-C=O), 41.75 (O=C-CH-CH_2_), 35.90 (N-C, pyrazole-CH_3_), 26.16 (CH(CH_3_)_2_), 22.11 (CH(CH_3_)_2_), 21.78 (CH(CH_3_)_2_), 10.85 (pyrazole-CH_3_), 9.15 (pyrazole-CH_3_). HRMS calcd. for C_24_H_30_F_6_N_4_O_4_ ([M+H]^+^), 553.2244; found, 553.2236.

*N^1^-(4-(1,1,1,3,3,3-Hexafluoro-2-methoxypropan-2-yl)-2-methoxyphenyl)-2-isopentyl-N^3^-(1,3,5-trimethyl-1H-pyrazol-4-yl)malonamide* (**8m**): Light yellow solid; m.p. 70–72 °C; yield 47.2%; ^1^H-NMR (300 MHz, Chloroform-d): δ 9.32 (s, 1H, pyrazole-NH), 8.43 (d, *J* = 8.6 Hz, 1H, Ar-H), 8.17 (s, 1H, Ar-NH), 7.17 (d, *J* = 8.4 Hz, 1H, Ar-H), 7.06 (s, 1H, Ar-H), 3.92 (s, 3H, Ar-OCH_3_), 3.69 (s, 3H, N-CH_3_), 3.49 (s, 3H, OCH_3_), 3.36 (t, *J* = 7.5 Hz, 1H, O=C-CH-C=O), 2.12 (s, 6H, pyrazole-CH_3_), 2.09-2.03 (m, 2H, O=C-CH-CH_2_), 1.67–1.54 (m, 1H, CH(CH_3_)_2_), 1.40–1.31 (m, 2H, CH_2_-CH(CH_3_)_2_), 0.88 (d, *J* = 6.6 Hz, 6H, CH(CH_3_)_2_). ^13^C-NMR (75 MHz, Chloroform-d): δ 170.05 (C=O), 169.01 (C=O), 148.23 (Ar-C_2_), 142.71 (C=N, pyrazole-C_3_), 134.47 (C-N, Ar-C_1_), 128.71 (C-N-N, pyrazole-C_5_), 122.98 (Ar-C_4_), 120.78 (Ar-C_5_), 122.04 (q, *J*_CF_ = 288.8 Hz, CF_3_), 119.34 (Ar-C_6_), 113.74 (C-N, pyrazole-C_4_), 109.48 (Ar-C_3_), 82.45 (hept, *J*_CF_ = 28.5 Hz, C(CF_3_)_2_), 56.01 (Ar-OCH_3_), 55.63 (O=C-C-C=O), 53.90 (OCH_3_), 36.27 (N-C, pyrazole-CH_3_), 35.88 (CH_2_-CH(CH_3_)_2_), 31.01 (CH(CH_3_)_2_), 27.43 (O=C-CH-CH_2_), 22.01 (CH(CH_3_)_2_), 21.92 (CH(CH_3_)_2_), 10.88 (pyrazole-CH_3_), 9.16 (pyrazole-CH_3_). HRMS calcd. for C_25_H_32_F_6_N_4_O_4_ ([M+H]^+^), 567.2401; found, 567.2391.

*N^1^-(4-(1,1,1,3,3,3-Hexafluoro-2-methoxypropan-2-yl)-2-methoxyphenyl)-2-(prop-2-yn-1-yl)-N^3^-(1,3,5-trimethyl-1H-pyrazol-4-yl)malonamide* (**8n**): Light yellow solid; m.p. 178–180 °C; yield 60.2%; ^1^H-NMR (300 MHz, DMSO-d_6_): δ 9.64 (s, 1H, pyrazole-NH), 9.37 (s, 1H, Ar-NH), 8.31 (d, *J* = 8.6 Hz, 1H, Ar-H), 7.17 (d, *J* = 8.7 Hz, 1H, Ar-H), 7.08 (s, 1H, Ar-H), 3.92 (s, 3H, Ar-OCH_3_), 3.86 (t, *J* = 7.6 Hz, 1H, O=C-CH-C=O), 3.62 (s, 3H, N-CH_3_), 3.48 (s, 3H, OCH_3_), 2.93 (t, *J* = 2.5 Hz, 1H, C≡CH), 2.78–2.74 (m, 2H, CH_2_-C≡CH), 2.05 (s, 3H, pyrazole-CH_3_), 1.96 (s, 3H, pyrazole-CH_3_). ^13^C-NMR (75 MHz, DMSO-d_6_): δ 167.45 (C=O), 166.67 (C=O), 148.93 (Ar-C_2_), 141.74 (C=N, pyrazole-C_3_), 134.18 (C-N, Ar-C_1_), 129.47 (C-N-N, pyrazole-C_5_), 122.35 (q, *J*_CF_ = 288.8 Hz, CF_3_), 121.93 (Ar-C_4_), 120.64 (Ar-C_5_), 120.51 (Ar-C_6_), 114.70 (C-N, pyrazole-C_4_), 110.17 (Ar-C_3_), 82.47 (hept, *J*_CF_ = 28.5 Hz, C(CF_3_)_2_), 81.48 (C≡CH), 72.89 (C≡CH), 56.28 (Ar-OCH_3_), 54.48 (O=C-C-C=O), 52.50 (OCH_3_), 36.09 (N-C, pyrazole-CH_3_), 19.32 (CH_2_-C≡CH), 11.15 (pyrazole-CH_3_), 9.05 (pyrazole-CH_3_). HRMS calcd. for C_23_H_24_F_6_N_4_O_4_ ([M+H]^+^), 535.1775; found, 535.1768.

*2-Allyl-N^1^-(4-(1,1,1,3,3,3-hexafluoro-2-methoxypropan-2-yl)-2-methoxyphenyl)-N^3^-(1,3,5-trimethyl-1H-pyrazol-4-yl)malonamide* (**8o**): White solid; m.p. 190–191 °C; yield 55.2%; ^1^H-NMR (300 MHz, Chloroform-*d*): δ 9.45 (s, 1H, pyrazole-NH), 8.40 (d, *J* = 8.6 Hz, 1H, Ar-H), 8.34 (s, 1H, Ar-NH), 7.15 (d, *J* = 8.6 Hz, 1H, Ar-H), 7.05 (s, 1H, Ar-H), 5.91-5.77 (m, 1H, CH=CH_2_), 5.25-5.08 (m, 2H, CH=CH_2_), 3.90 (s, 3H, Ar-OCH_3_), 3.66 (s, 3H, N-CH_3_), 3.50 (t, *J* = 7.5 Hz, 1H, O=C-CH-C=O), 3.48 (s, 3H, OCH_3_), 2.84-2.76 (m, 2H, O=C-CH-CH_2_), 2.09 (d, *J* = 2.7 Hz, 6H, pyrazole-CH_3_). ^13^C-NMR (75 MHz, Chloroform-*d*): δ 169.48 (C=O), 168.11 (C=O), 148.29 (Ar-C_2_), 142.71 (C=N, pyrazole-C_3_), 134.55 (C-N, Ar-C_1_), 133.23 (CH=CH_2_), 128.67 (C-N-N, pyrazole-C_5_), 123.00 (Ar-C_4_), 122.03 (q, *J*_CF_ = 288.8 Hz, CF_3_), 120.74 (Ar-C_5_), 119.40 (Ar-C_6_), 118.05 (CH=CH_2_), 113.72 (C-N, pyrazole-C_4_), 109.50 (Ar-C_3_), 82.44 (hept, *J*_CF_ = 28.5 Hz, C(CF_3_)_2_), 55.61 (Ar-OCH_3_), 55.14 (O=C-C-C=O), 53.88 (OCH_3_), 36.74 (N-C, pyrazole-CH_3_), 35.83 (O=C-CH-CH_2_), 10.85 (pyrazole-CH_3_), 9.10 (pyrazole-CH_3_). HRMS calcd. for C_23_H_26_F_6_N_4_O_4_ ([M+H]^+^), 537.1931; found, 537.1922.

*N^1^-(4-(1,1,1,3,3,3-Hexafluoro-2-methoxypropan-2-yl)-3-isopropylphenyl)-2-isopropyl-N^3^-(1,3,5-trimethyl-1H-pyrazol-4-yl)malonamide* (**8p**): White solid; m.p. 86–88 °C; yield 51.7%; ^1^H-NMR (300 MHz, Chloroform-d): δ 9.46 (s, 1H, pyrazole-NH), 8.39 (d, *J* = 1.5 Hz, 1H, Ar-H), 8.06 (s, 1H, Ar-NH), 7.41 (d, *J* = 8.4 Hz, 1H, Ar-H), 7.08 (dd, *J* = 8.5, 1.8 Hz, 1H, Ar-H), 3.68 (s, 3H, N-CH_3_), 3.56 (s, 3H, OCH_3_), 2.97 (d, *J* = 10.4 Hz, 1H, O=C-CH-C=O), 2.95–2.86 (m, 1H, Ar-CH(CH_3_)_2_), 2.53–2.38 (m, 1H, CH(CH_3_)_2_), 2.09 (d, *J* = 5.2 Hz, 6H, pyrazole-CH_3_), 1.26 (d, *J* = 6.9 Hz, 6H, Ar-CH(CH_3_)_2_), 1.12 (dd, *J* = 17.5, 6.6 Hz, 6H, CH(CH_3_)_2_). ^13^C-NMR (75 MHz, Chloroform-d): δ 168.66 (C=O), 168.48 (C=O), 151.92 (Ar-C_3_), 142.66 (C=N, pyrazole-C_3_), 137.24 (C-N-N, pyrazole-C_5_), 134.50 (C-N, Ar-C_1_), 129.37 (Ar-C_4_), 122.40 (Ar-C_5_), 122.04 (q, *J*_CF_ = 288.8 Hz, CF_3_), 121.35 (Ar-C_6_), 113.80 (C-N, pyrazole-C_4_), 112.11 (Ar-C_2_), 84.49 (hept, *J*_CF_ = 28.5 Hz, C(CF_3_)_2_), 65.18 (O=C-C-C=O), 54.28 (OCH_3_), 35.89 (N-C, pyrazole-CH_3_), 33.50 (Ar-CH(CH_3_)_2_), 31.66 (CH(CH_3_)_2_), 23.08 (Ar-CH(CH_3_)_2_), 20.36 (CH(CH_3_)_2_), 20.28 (CH(CH_3_)_2_), 10.80 (pyrazole-CH_3_), 9.12 (pyrazole-CH_3_). HRMS calcd. for C_25_H_32_F_6_N_4_O_3_ ([M+H]^+^), 551.2451; found, 551.2443.

*N^1^-(4-(1,1,1,3,3,3-Hexafluoro-2-methoxypropan-2-yl)-3-isopropylphenyl)-2-isobutyl-N^3^-(1,3,5-trimethyl-1H-pyrazol-4-yl)malonamide* (**8q**): White solid; m.p. 67–69 °C; yield 48.8%; ^1^H-NMR (300 MHz, Chloroform-d): δ 9.41 (s, 1H, pyrazole-NH), 8.33 (d, *J* = 1.8 Hz, 1H, Ar-H), 8.02 (s, 1H, Ar-NH), 7.39 (d, *J* = 8.4 Hz, 1H, Ar-H), 7.06 (dd, *J* = 8.5, 1.8 Hz, 1H, Ar-H), 3.66 (s, 3H, N-CH_3_), 3.54 (s, 3H, OCH_3_), 3.45 (t, *J* = 7.6 Hz, 1H, O=C-CH-C=O), 2.94–2.85 (m, 1H, Ar-CH(CH_3_)_2_), 2.06 (d, *J* = 4.7 Hz, 6H, pyrazole-CH_3_), 2.00–1.83 (m, 2H, O=C-CH-CH_2_), 1.77–1.68 (m, 1H, CH(CH_3_)_2_), 1.24 (d, *J* = 6.9 Hz, 6H, Ar-CH(CH_3_)_2_), 0.99 (dd, *J* = 6.5, 4.0 Hz, 6H, CH(CH_3_)_2_). ^13^C-NMR (75 MHz, Chloroform-d): δ 169.08 (C=O), 169.02 (C=O), 151.90 (Ar-C_3_), 142.67 (C=N, pyrazole-C_3_), 137.17 (C-N-N, pyrazole-C_5_), 134.54 (C-N, Ar-C_1_), 129.36 (Ar-C_4_), 122.46 (Ar-C_5_), 122.09 (q, *J*_CF_ = 288.8 Hz, CF_3_), 121.57 (Ar-C_6_), 113.79 (C-N, pyrazole-C_4_), 112.21 (Ar-C_2_), 84.49 (hept, *J*_CF_ = 28.5 Hz, C(CF_3_)_2_), 55.20 (OCH_3_), 54.27 (O=C-C-C=O), 40.72 (O=C-CH-CH_2_), 35.87 (N-C, pyrazole-CH_3_), 33.50 (Ar-CH(CH_3_)_2_), 26.04 (CH(CH_3_)_2_), 23.07 (Ar-CH(CH_3_)_2_), 22.10 (CH(CH_3_)_2_), 21.81 (CH(CH_3_)_2_), 10.70 (pyrazole-CH_3_), 9.05 (pyrazole-CH_3_). HRMS calcd. for C_26_H_34_F_6_N_4_O_3_ ([M+H]^+^), 565.2608; found, 565.2601.

*N^1^-(4-(1,1,1,3,3,3-Hexafluoro-2-methoxypropan-2-yl)-3-isopropylphenyl)-2-isopentyl-N^3^-(1,3,5-trimethyl-1H-pyrazol-4-yl)malonamide* (**8r**): White solid; m.p. 63–64 °C; yield 54.8%; ^1^H-NMR (300 MHz, Chloroform-d): δ 9.40 (s, 1H, pyrazole-NH), 8.33 (d, *J* = 1.7 Hz, 1H, Ar-H), 7.97 (s, 1H, Ar-NH), 7.39 (d, *J* = 8.4 Hz, 1H, Ar-H), 7.07 (dd, *J* = 8.5, 1.9 Hz, 1H, Ar-H), 3.66 (s, 3H, N-CH_3_), 3.54 (s, 3H, OCH_3_), 3.27 (t, *J* = 7.5 Hz, 1H, O=C-CH-C=O), 2.98–2.84 (m, 1H, Ar-CH(CH_3_)_2_), 2.07 (d, *J* = 4.4 Hz, 6H, pyrazole-CH_3_), 2.05–1.97 (m, 2H, O=C-CH-CH_2_), 1.68–1.55 (m, 1H, CH(CH_3_)_2_), 1.37–1.29 (m, 2H, CH_2_-CH(CH_3_)_2_), 1.25 (d, *J* = 6.9 Hz, 6H, Ar-CH(CH_3_)_2_), 0.90 (d, *J* = 6.6 Hz, 6H, CH(CH_3_)_2_). ^13^C-NMR (75 MHz, Chloroform-d): δ 169.04 (C=O), 168.96 (C=O), 151.92 (Ar-C_3_), 142.68 (C=N, pyrazole-C_3_), 137.15 (C-N-N, pyrazole-C_5_), 134.56 (C-N, Ar-C_1_), 129.36 (Ar-C_4_), 122.49 (Ar-C_5_), 122.09 (q, *J*_CF_ = 288.8 Hz, CF_3_), 121.66 (Ar-C_6_), 113.77 (C-N, pyrazole-C_4_), 112.25 (Ar-C_2_), 84.49 (hept, *J*_CF_ = 28.5 Hz, C(CF_3_)_2_), 57.03 (O=C-C-C=O), 54.27 (OCH_3_), 36.24 (N-C, pyrazole-CH_3_), 35.88 (CH_2_-CH(CH_3_)_2_), 33.50 (Ar-CH(CH_3_)_2_), 29.94 (CH_2_-CH(CH_3_)_2_), 27.47 (O=C-CH-CH_2_), 23.08 (Ar-CH(CH_3_)_2_), 22.06 (CH_2_-CH(CH_3_)_2_), 21.88 (CH_2_-CH(CH_3_)_2_), 10.74 (pyrazole-CH_3_), 9.07 (pyrazole-CH_3_). HRMS calcd. for C_27_H_36_F_6_N_4_O_3_ ([M+H]^+^), 579.2764; found, 579.2756.

*N^1^-(4-(1,1,1,3,3,3-Hexafluoro-2-methoxypropan-2-yl)-3-isopropylphenyl)-2-(prop-2-yn-1-yl)-N^3^-(1,3,5-trimethyl-1H-pyrazol-4-yl)malonamide* (**8s**): White solid; m.p. 69–71 °C; yield 56.5%; ^1^H-NMR (300 MHz, Chloroform-d): δ 9.44 (s, 1H, pyrazole-NH), 8.27 (d, *J* = 1.7 Hz, 1H, Ar-H), 7.93 (s, 1H, Ar-NH), 7.39 (d, *J* = 8.4 Hz, 1H, Ar-H), 7.08 (dd, *J* = 8.5, 1.9 Hz, 1H, Ar-H), 3.66 (s, 3H, N-CH_3_), 3.56–3.51 (m, 4H, O=C-CH-C=O and O-CH_3_), 2.98–2.93 (m, 2H, CH_2_-C≡CH), 2.93–2.86 (m, 1H, Ar-CH(CH_3_)_2_), 2.12 (t, *J* = 2.6 Hz, 1H, C≡CH), 2.08 (d, *J* = 6.1 Hz, 6H, pyrazole-CH_3_), 1.25 (d, *J* = 6.9 Hz, 6H, Ar-CH(CH_3_)_2_). ^13^C-NMR (75 MHz, Chloroform-d): δ 167.52 (C=O), 166.65 (C=O), 151.97 (Ar-C_3_), 142.79 (C=N, pyrazole-C_3_), 136.79 (C-N-N, pyrazole-C_5_), 134.83 (C-N, Ar-C_1_), 129.37 (Ar-C_4_), 122.78 (Ar-C_5_), 122.14 (Ar-C_6_), 122.09 (q, *J*_CF_ = 288.8 Hz, CF_3_), 113.54 (C-N, pyrazole-C_4_), 112.65 (Ar-C_2_), 84.43 (hept, *J*_CF_ = 28.5 Hz, C(CF_3_)_2_), 79.52 (C≡CH), 71.27 (C≡CH), 54.86 (O=C-C-C=O), 54.30 (OCH_3_), 35.87 (N-C, pyrazole-CH_3_), 33.47 (Ar-CH(CH_3_)_2_), 23.07 (Ar-CH(CH_3_)_2_), 20.88 (CH_2_-C≡CH), 10.71 (pyrazole-CH_3_), 9.05 (pyrazole-CH_3_). HRMS calcd. for C_25_H_28_F_6_N_4_O_3_ ([M+H]^+^), 547.2138; found, 547.2131.

*2-Allyl-N^1^-(4-(1,1,1,3,3,3-hexafluoro-2-methoxypropan-2-yl)-3-isopropylphenyl)-N^3^-(1,3,5-trimethyl-1H-pyrazol-4-yl)malonamide* (**8t**): White solid; m.p. 58–60 °C; yield 56.8%; ^1^H-NMR (300 MHz, Chloroform-*d*): δ 9.39 (s, 1H, pyrazole-NH), 8.28 (d, *J* = 1.7 Hz, 1H, Ar-H), 7.90 (s, 1H, Ar-NH), 7.39 (d, *J* = 8.5 Hz, 1H, Ar-H), 7.08 (dd, *J* = 8.5, 1.9 Hz, 1H, Ar-H), 5.92-5.78 (m, 1H, CH=CH_2_), 5.29–5.10 (m, 2H, CH=CH_2_), 3.66 (s, 3H, N-CH_3_), 3.54 (s, 3H, O-CH_3_), 3.38 (t, *J* = 7.5 Hz, 1H, O=C-CH-C=O), 2.96-2.87 (m, 1H, Ar-CH(CH_3_)_2_), 2.80 (t, *J* = 7.2 Hz, 2H, O=C-CH-CH_2_), 2.06 (d, *J* = 4.9 Hz, 6H, pyrazole-CH_3_), 1.25 (d, *J* = 6.9 Hz, 6H, Ar-CH(CH_3_)_2_). ^13^C-NMR (75 MHz, Chloroform-*d*): δ 168.30 (C=O), 168.14 (C=O), 151.95 (Ar-C_3_), 142.71 (C=N, pyrazole-C_3_), 136.97 (C-N-N, pyrazole-C_5_), 134.66 (C-N, Ar-C_1_), 133.18 (CH=CH_2_), 129.37 (Ar-C_4_), 122.09 (q, *J*_CF_ = 288.8 Hz, CF_3_), 122.66 (Ar-C_5_), 121.87 (Ar-C_6_), 118.12 (CH=CH_2_), 113.60 (C-N, pyrazole-C_4_), 112.42 (Ar-C_2_), 84.47 (hept, *J*_CF_ = 28.5 Hz, C(CF_3_)_2_), 56.32 (O=C-C-C=O), 54.28 (OCH_3_), 35.89 (N-C, pyrazole-CH_3_), 35.86 (O=C-CH-CH_2_), 33.48 (Ar-CH(CH_3_)_2_), 23.09 (Ar-CH(CH_3_)_2_), 10.72 (pyrazole-CH_3_), 9.08 (pyrazole-CH_3_). HRMS calcd. for C_25_H_30_F_6_N_4_O_3_ ([M+H]^+^), 549.2295; found, 549.2287.

### 3.3. Bioassay Methods

All of the biological assays were performed on representative test organisms reared in the laboratory. Each bioassay was repeated at 25 ± 1 °C according to statistical requirements. All compounds were dissolved in dimethyl sulfoxide and diluted with distilled water containing Triton X-100 to obtain a series of concentrations. Control check (CK) was treated as the same without compounds. Assessments were based on a dead/alive scale, and mortality rates were rectified using Abbott’s formula [27]. Evaluations of the activities of the compounds were based on a percentage scale of 0 to 100, where 0 equals no activity, and 100 equals total kill. The error of the experiments was kept within 5.0%. Given the lack of appropriate methods and purchasing channels to obtain the pyflubumide standard in our present work, commercial insecticides, such as fenpyroximate, flubendiamide, and imidacloprid, were selected as positive controls under the same conditions.

#### 3.3.1. Acaricidal Activity Against *Tetranychus Cinnabarinus*

The acaricidal activities of the target compounds and fenpyroximate against *Tetranychus cinnabarinus* were evaluated using the reported procedure [28]. Sieva bean plants (*Phaseolus vulgaris*) with primary leaves expanded to 10 cm in size were selected and cut back to one plant per pot. A small piece was cut from a leaf taken from the main colony and placed on each leaf of the test plants. This was done approximately 2 h before treatment to allow the mites to move to the test plant and lay eggs. The size of the leaf piece from the main colony was varied to obtain approximately 30 mites per leaf. At the time of the treatment, the piece of leaf used to transfer the mites was removed and discarded. The mite-infested plants were dipped in the test solution for 3 s with agitation and set in a fume hood to dry. Plants were kept for 48 h before the numbers of live and dead adults were counted. Each treatment was repeated with triplicate experiments, and each replicate involved 30 adult mites.

#### 3.3.2. Insecticidal Activity Against *Plutella xylostella*

The insecticidal activities of the target compounds and flubendiamide against *Plutella xylostella* were evaluated using the reported procedure [29]. Leaf disks (6 cm × 2 cm) were cut from fresh cabbage leaves and then dipped into the test solution for 5 s. After air-drying, the treated leaf disks were placed individually into glass tubes. Each dried treated leaf disk was infested with 10 third-instar *Plutella xylostella* larvae. Mortality was assessed 48 h after treatment. Each treatment was repeated with triplicate experiments and each replicate involved 10 third-instar *Plutella xylostella* larvae.

#### 3.3.3. Insecticidal Activity Against *Aphis craccivora*

The insecticidal activities of the target compounds and imidacloprid against *Aphis craccivora* were evaluated using the reported procedure [30]. Leaves from the soybean plant with 20 apterous adults were dipped in the test solution for 5 s and the excess solution was blotted with filter paper. The mortality rates were evaluated 36 h after treatment. Each treatment was repeated with triplicate experiments and each replicate involved 20 apterous adults.

## 4. Conclusions

In summary, twenty novel 1,3,5-trimethylpyrazole-containing malonamide derivatives were designed and synthesized. The target compounds were evaluated for acaricidal and insecticidal activities against *Tetranychus cinnabarinus*, *Plutella xylostella,* and *Aphis craccivora*. Preliminary bioassay results indicated that compounds **8m** and **8p** were found to have moderate activities against *Tetranychus cinnabarinus*. Compounds **8i** and **8o** exhibited good insecticidal activities against *Plutella xylostella*. In addition, compounds **8p** and **8q** possessed potent insecticidal activities against *Aphis craccivora*. Although the target compounds did not exhibit excellent acaricidal activities against *Tetranychus cinnabarinus*, as we expected, some of the target compounds showed good insecticidal activities against *Plutella xylostella* and *Aphis craccivora*, respectively. The preliminary analyses of structure–activity relationships (SAR) indicated that the methoxy-substituted hexafluoroisopropyl group and variations of Y groups in the position of phenyl ring markedly affected the acaricidal and insecticidal activities. In addition, different types of substituents (R_2_) have a great influence on the biological activity of different pest species. Notably, these findings demonstrated that the synthesis of 1,3,5-trimethylpyrazole-containing malonamide derivatives could be considered as a new template for pesticide development. Further structural optimization and bioactivities are in progress.

## Figures and Tables

**Table 1:** Insecticidal activities of compounds **8a**~**8t** and fenpyroximate (FEN) against *Tetranychus cinnabarinus*.

Compound	Mortality (%) ^a^					LC_50_ (µg/mL)	95% CL (µg/mL)
	400 µg/mL	200 µg/mL	100 µg/mL	50 µg/mL	20 µg/mL		
**8a**	90.0 ± 3.3 ^b^	60.0 ± 5.8	24.4 ± 1.9	11.1 ± 1.9	0.0 ± 0.0	157.2	127.5–197.0
**8b**	85.6 ± 1.9	42.2 ± 1.9	20.0 ± 0.0	7.8 ± 1.9	0.0 ± 0.0	197.1	158.7–254.9
**8c**	60.0 ± 3.3	35.6 ± 1.9	0.0 ± 0.0	0.0 ± 0.0	0.0 ± 0.0	308.8	254.2–405.3
**8d**	61.1 ± 3.8	21.1 ± 1.9	0.0 ± 0.0	0.0 ± 0.0	0.0 ± 0.0	333.1	278.5–431.0
**8e**	31.1 ± 1.9	0.0 ± 0.0	0.0 ± 0.0	0.0 ± 0.0	0.0 ± 0.0	540.4	414.6–1988.6
**8f**	68.9 ± 3.8	31.1 ± 1.9	0.0 ± 0.0	0.0 ± 0.0	0.0 ± 0.0	290.7	244.6–360.1
**8g**	51.1 ± 3.8	20.0 ± 3.3	0.0 ± 0.0	0.0 ± 0.0	0.0 ± 0.0	376.7	305.6–544.9
**8h**	78.9 ± 3.8	41.1 ± 1.9	0.0 ± 0.0	0.0 ± 0.0	0.0 ± 0.0	250.7	212.9–298.7
**8i**	100.0 ± 0.0	64.4 ± 1.9	30.0 ± 3.3	17.8 ± 1.9	7.8 ± 1.9	119.2	52.0–343.6
**8j**	90.0 ± 3.3	54.4 ± 1.9	20.0 ± 3.3	7.8 ± 1.9	0.0 ± 0.0	172.2	140.8–214.8
**8k**	100.0 ± 0.0	60.0 ± 3.3	20.0 ± 0.0	8.9 ± 1.9	0.0 ± 0.0	149.5	124.8–180.9
**8l**	83.3 ± 3.3	51.1 ± 1.9	20.0 ± 5.8	5.6 ± 1.9	0.0 ± 0.0	190.9	154.8–242.8
**8m**	90.0 ± 0.0	70.0 ± 3.3	35.6 ± 1.9	27.8 ± 1.9	13.3 ± 3.3	107.2	79.9–145.5
**8n**	100.0 ± 0.0	50.0 ± 3.3	20.0 ± 3.3	10.0 ± 0.0	0.0 ± 0.0	158.9	84.2–379.2
**8o**	80.0 ± 3.3	40.0 ± 0.0	0.0 ± 0.0	0.0 ± 0.0	0.0 ± 0.0	250.3	212.9–297.1
**8p**	100.0 ± 0.0	70.0 ± 3.3	37.8 ± 1.9	15.6 ± 1.9	5.6 ± 1.9	112.2	90.2–140.4
**8q**	100.0 ± 0.0	50.0 ± 3.3	20.0 ± 0.0	5.6 ± 1.9	0.0 ± 0.0	164.0	103.3–288.0
**8r**	100.0 ± 0.0	60.0 ± 0.0	25.6 ± 1.9	12.2 ± 1.9	7.8 ± 1.9	132.3	39.7–1172.9
**8s**	85.6 ± 1.9	43.3 ± 3.3	20.0 ± 3.3	8.9 ± 1.9	0.0 ± 0.0	194.2	156.0–252.1
**8t**	38.9 ± 1.9	18.9 ± 3.8	0.0 ± 0.0	0.0 ± 0.0	0.0 ± 0.0	460.3	348.5–879.2
FEN	100.0 ± 0.0	100.0 ± 0.0	100.0 ± 0.0	100.0 ± 0.0	100.0 ± 0.0	—	—

^a^ Experimental size: 30 insects per group, three groups. ^b^ Each value represents the mean ± standard deviation of three replications; LC_50_ = 50% lethal concentration; 95% CL = 95% confidence limit; “—” refers to “not calculated”.

**Table 2:** Insecticidal activities of compounds **8a**–**8t** and flubendiamide (FLU) against *Plutella xylostellas*.

Compound	Mortality (%) ^a^					LC_50_ (µg/mL)	95% CL (µg/mL)
	400 µg/mL	200 µg/mL	100 µg/mL	50 µg/mL	20 µg/mL		
**8a**	80.0 ± 10.0 ^b^	60.0 ± 0.0	30.0 ± 10.0	16.7 ± 5.8	10.0 ± 0.0	153.2	89.8–333.2
**8b**	53.3 ± 5.8	30.0 ± 0.0	16.7 ± 5.8	6.7 ± 5.8	0.0 ± 0.0	359.0	204.7–2371.2
**8c**	63.3 ± 5.8	36.7 ± 5.8	10.0 ± 0.0	6.7 ± 5.8	0.0 ± 0.0	289.3	183.8–808.9
**8d**	90.0 ± 0.0	73.3 ± 5.8	30.0 ± 0.0	23.3 ± 5.8	16.7 ± 5.8	109.5	63.0–204.2
**8e**	100.0 ± 0.0	76.7 ± 5.8	33.3 ± 5.8	26.7 ± 5.8	13.3 ± 5.8	94.5	58.7–153.6
**8f**	20.0 ± 0.0	0.0 ± 0.0	0.0 ± 0.0	0.0 ± 0.0	0.0 ± 0.0	—	—
**8g**	100.0 ± 0.0	100.0 ± 0.0	43.3 ± 5.8	20.0 ± 0.0	6.7 ± 5.8	82.6	57.2–119.3
**8h**	86.7 ± 5.8	60.0 ± 0.0	30.0 ± 10.0	13.3 ± 5.8	0.0 ± 0.0	154.0	102.2–250.5
**8i**	100.0 ± 0.0	100.0 ± 0.0	100.0 ± 0.0	83.3 ± 5.8	46.7 ± 5.8	22.1	6.6–33.3
**8j**	70.0 ± 0.0	33.3 ± 5.8	20.0 ± 0.0	6.7 ± 5.8	0.0 ± 0.0	257.3	164.4–636.1
**8k**	60.0 ± 0.0	50.0 ± 0.0	30.0 ± 0.0	16.7 ± 5.8	6.7 ± 5.8	235.4	124.0–1322.9
**8l**	70.0 ± 0.0	46.7 ± 5.8	26.7 ± 5.8	10.0 ± 0.0	0.0 ± 0.0	93.3	57.9–149.3
**8m**	63.3 ± 5.8	30.0 ± 10.0	20.0 ± 0.0	6.7 ± 5.8	0.0 ± 0.0	294.2	179.9–1000.9
**8n**	100.0 ± 0.0	66.7 ± 5.8	40.0 ± 0.0	23.3 ± 5.8	13.3 ± 5.8	99.9	61.2–166.9
**8o**	100.0 ± 0.0	100.0 ± 0.0	100.0 ± 0.0	76.7 ± 5.8	43.3 ± 5.8	24.2	8.9–36.5
**8p**	30.0 ± 0.0	10.0 ± 0.0	0.0 ± 0.0	0.0 ± 0.0	0.0 ± 0.0	569.8	405.6–2013.3
**8q**	76.7 ± 5.8	40.0 ± 0.0	20.0 ± 0.0	10.0 ± 0.0	0.0 ± 0.0	220.0	142.9–454.3
**8r**	56.7 ± 5.8	26.7 ± 5.8	10.0 ± 0.0	6.7 ± 5.8	0.0 ± 0.0	359.0	213.2–1857.2
**8s**	90.0 ± 0.0	56.7 ± 5.8	26.7 ± 5.8	13.3 ± 5.8	6.7 ± 5.8	149.9	95.6–268.6
**8t**	30.0 ± 0.0	0.0 ± 0.0	0.0 ± 0.0	0.0 ± 0.0	0.0 ± 0.0	—	—
FLU	100.0 ± 0.0	100.0 ± 0.0	100.0 ±0.0	100.0 ± 0.0	100.0 ± 0.0	—	—

^a^ Experimental size: 10 insects per group, three groups. ^b^ Each value represents the mean ± standard deviation of three replications; LC_50_ = 50% lethal concentration; 95% CL = 95% confidence limit; “—” refers to “not calculated”.

**Table 3:** Insecticidal activities of compounds **8a**~**8t** and imidacloprid (IMI) against *Aphis craccivora*.

Compound	Mortality (%) ^a^					LC_50_ (µg/mL)	95% CL (µg/mL)
	200 µg/mL	100 µg/mL	50 µg/mL	20 µg/mL	10 µg/mL		
**8a**	100.0 ± 0.0 ^b^	100.0 ± 0.0	80.0 ± 0.0	56.7 ± 2.9	21.7 ± 2.9	19.1	13.5–25.4
**8b**	100.0 ± 0.0	100.0 ± 0.0	70.0 ± 0.0	43.3 ± 2.9	20.0 ± 5.0	23.3	16.8–31.1
**8c**	100.0 ± 0.0	90.0 ± 0.0	65.0 ± 0.0	36.7 ± 2.9	15.0 ± 0.0	29.0	20.8–39.1
**8d**	71.7 ± 2.9	45.0 ± 0.0	20.0 ± 0.0	13.3 ± 2.9	8.3 ± 2.9	111.7	73.0–224.0
**8e**	40.0 ± 0.0	20.0 ± 0.0	0.0 ± 0.0	0.0 ± 0.0	0.0 ± 0.0	225.3	162.9–612.0
**8f**	100.0 ± 0.0	81.7 ± 2.9	58.3 ± 2.9	31.7 ± 2.9	18.3 ± 2.9	32.6	22.7–45.1
**8g**	90.0 ± 0.0	70.0 ± 0.0	50.0 ± 5.0	26.7 ± 2.9	11.7 ± 2.9	46.6	32.0–68.0
**8h**	80.0 ± 5.0	50.0 ± 5.0	30.0 ± 0.0	13.3 ± 2.9	8.3 ± 2.9	85.0	58.3–141.1
**8i**	100.0 ± 0.0	75.0 ± 5.0	50.0 ± 0.0	28.3 ± 5.8	16.7 ± 2.9	38.3	26.8–53.7
**8j**	100.0 ± 0.0	91.7 ± 2.9	60.0 ± 5.0	36.7 ± 2.9	21.7 ± 2.9	27.6	19.2–37.9
**8k**	100.0 ± 0.0	100.0 ± 0.0	76.7 ± 2.9	41.7 ± 2.9	25.0 ± 0.0	21.4	15.2–28.5
**8l**	71.7 ± 2.9	50.0 ± 5.0	40.0 ± 0.0	26.7 ± 2.9	13.3 ± 2.9	79.2	47.6–173.5
**8m**	100.0 ± 0.0	80.0 ± 0.0	50.0 ± 5.0	30.0 ± 5.0	18.3 ± 2.9	35.7	24.9–49.8
**8n**	90.0 ± 0.0	68.3 ± 2.9	50.0 ± 5.0	30.0 ± 0.0	18.3 ± 2.9	42.9	27.8–65.3
**8o**	75.0 ± 0.0	50.0 ± 5.0	30.0 ± 0.0	18.3 ± 2.9	10.0 ± 0.0	88.4	57.7–166.1
**8p**	100.0 ± 0.0	100.0 ± 0.0	100.0 ± 0.0	88.3 ± 2.9	63.3 ± 2.9	8.1	1.8–11.3
**8q**	100.0 ± 0.0	100.0 ± 0.0	100.0 ± 0.0	83.3 ± 2.9	63.3 ± 2.9	8.1	2.1–11.6
**8r**	100.0 ± 0.0	70.0 ± 5.0	60.0 ± 0.0	43.3 ± 2.9	20.0 ± 5.0	30.9	19.7–44.9
**8s**	90.0 ± 5.0	65.0 ± 5.0	45.0 ± 0.0	25.0 ± 0.0	13.3 ± 2.9	50.7	34.5–76.0
**8t**	50.0 ± 5.0	30.0 ± 5.0	0.0 ± 0.0	0.0 ± 0.0	0.0 ± 0.0	181.2	137.8–320.3
IMI	100.0 ± 0.0	100.0 ± 0.0	100.0 ± 0.0	100.0 ± 0.0	100.0 ± 0.0	—	—

^a^ Experimental size: 20 insects per group, three groups. ^b^ Each value represents the mean ± standard deviation of three replications; LC_50_ = 50% lethal concentration; 95% CL = 95% confidence limit; “—” refers to “not calculated”.

## Supplementary Materials

The spectra of ^1^H-NMR, ^13^C-NMR, and HRMS for all the synthesized compounds are available online.
