# Supplementary material for: Design, Synthesis, and Biological Activities of Novel 1,3,5-Trimethylpyrazole-Containing Malonamide Derivatives
Source: Molecules. 2019 Feb 3;24(3):562. doi: 10.3390/molecules24030562 (PMC6385117; doi:10.3390/molecules24030562)

Supporting information

Design, synthesis, and biological activities of novel 1,3,5-trimethylpyrazole-containing malonamide derivatives

Qi-Bo Li [1], Min Liao [1], Qing Liu [1], Tong Feng [1], Zhi-Yuan Xu [1], Chang-Hui Rui [2,*], and Shang-Zhong Liu [1,*]

[1] Department of Applied Chemistry, College of Science, China Agricultural University, Beijing 100193, China; lqb@cau.edu.cn (Q.-B.L.); cygnus@cau.edu.cn (M.L.); qingqing@cau.edu.cn (Q.L.); fibber@cau.edu.cn (T.F.); xuzhiyuan@cau.edu.cn (Z.-Y.X.)

[2] Key Laboratory of Integrated Pest Management in Crops, Ministry of Agriculture, Institute of Plant Protection, Chinese Academy of Agricultural Sciences, Beijing 100193, China;

[1,*]Correspondence: shangzho@cau.edu.cn. Tel: +86-10-62731070. (S.-Z.L.)

[2,*]Correspondence: huirch@163.com. Tel: +86-10-62815944. (C.-H.R.)

Content

The identification date of intermediate **3** and **6** 2

Table S1. The structures of intermediate **3**, **6** and all target compounds 3

^{1}H NMR spectrums of intermediate **3**, **6** and all target compounds 6

^{13}C NMR spectrums of all target compounds 20

HRMS spectrums of all target compounds 30

The identification date of intermediate **3** and **6**

Ethyl 3-((2-methyl-4-(perfluoropropan-2-yl)phenyl)amino)-3-oxopropanoate (intermediate **3-1**): White solid; m.p. 95-96 °C; yield 93.5%; ^{1}H NMR (300 MHz, Chloroform-*d*): δ 9.64 (s, 1H, NH), 8.27 (d, *J* = 8.6 Hz, 1H, Ar-H), 7.45-7.41 (m, 2H, Ar-H), 4.27 (q, *J* = 7.2 Hz, 2H, OCH_2CH_3), 3.52 (s, 2H, O=C-CH_2-C=O), 2.39 (s, 3H, Ar-CH_3), 1.33 (t, *J* = 7.2 Hz, 3H, OCH_2CH_3).

Ethyl 3-((4-(1,1,1,3,3,3-hexafluoro-2-methoxypropan-2-yl)-2-methylphenyl)amino)-3-oxopropanoate (intermediate **3-2**): Light yellow solid; m.p. 62-63 °C; yield 91.6%; ^{1}H NMR (300 MHz, Chloroform-*d*): δ 9.57 (s, 1H, NH), 8.21 (d, *J* = 8.6 Hz, 1H, Ar-H), 7.47-7.34 (m, 2H, Ar-H), 4.28 (q, *J* = 7.1 Hz, 2H, OCH_2CH_3), 3.53 (s, 2H, O=C-CH_2-C=O), 3.47 (s, 3H, OCH_3), 2.39 (s, 3H, Ar-CH_3), 1.33 (t, *J* = 7.2 Hz, 3H, OCH_2CH_3).

Ethyl 3-((4-(1,1,1,3,3,3-hexafluoro-2-methoxypropan-2-yl)-2-methoxyphenyl)amino)-3-oxopropanoate (intermediate **3-3**): Light yellow solid; m.p. 74-75 °C; yield 92.3%; ^{1}H NMR (300 MHz, Chloroform-*d*): δ 9.61 (s, 1H, NH), 8.45 (d, *J* = 8.6 Hz, 1H, Ar-H), 7.16 (d, *J* = 8.7 Hz, 1H, Ar-H), 7.04 (s, 1H, Ar-H), 4.25 (q, *J* = 7.2 Hz, 2H, OCH_2CH_3), 3.93 (s, 3H, Ar-OCH_3), 3.50 (s, 2H, O=C-CH_2-C=O), 3.47 (s, 3H, OCH_3), 1.31 (t, *J* = 7.2 Hz, 3H, OCH_2CH_3).

Ethyl 3-((4-(1,1,1,3,3,3-hexafluoro-2-methoxypropan-2-yl)-3-isopropylphenyl)amino)-3-oxopropanoate (intermediate **3-4**): Light yellow oil; yield 89.4%; ^{1}H NMR (300 MHz, Chloroform-*d*): δ 9.48 (s, 1H, NH), 8.27 (s, 1H, Ar-H), 7.39 (d, *J* = 8.4 Hz, 1H, Ar-H), 7.07 (dd, *J* = 8.5, 1.8 Hz, 1H, Ar-H), 4.24 (q, *J* = 7.1 Hz, 2H, OCH_2CH_3), 3.53 (s, 3H, OCH_3), 3.46 (s, 2H, O=C-CH_2-C=O), 2.93 (hept, *J* = 6.9 Hz, 1H, $CH(CH_3)_2$), 1.30 (t, *J* = 7.2 Hz, 3H, OCH_2CH_3), 1.25 (d, *J* = 6.9 Hz, 6H, $CH(CH_3)_2$).

N^1-(2-Methyl-4-(perfluoropropan-2-yl)phenyl)-N^3-(1,3,5-trimethyl-1H-pyrazol-4-yl)malonamide (intermediate **6-1**): White solid; m.p. 69-70 °C; yield 86.7%; ^{1}H NMR (300 MHz, DMSO-*d*$_6$): δ 9.91 (s, 1H, pyrazole-NH), 9.30 (s, 1H, Ar-NH), 7.95 (d, *J* = 8.4 Hz, 1H, Ar-H), 7.49-7.45 (m, 2H, Ar-H), 3.61 (s, 3H, N-CH_3), 3.53 (s, 2H, O=C-CH_2-C=O), 2.32 (s, 3H, Ar-CH_3), 2.05 (s, 3H, pyrazole-CH_3), 1.96 (s, 3H, pyrazole-CH_3).

N^1-(4-(1,1,1,3,3,3-Hexafluoro-2-methoxypropan-2-yl)-2-methylphenyl)-N^3-(1,3,5-trimethyl-1H-pyrazol-4-yl)malonamide (intermediate **6-2**): White solid; m.p. 65-66 °C; yield 82.5%; ^{1}H NMR (300 MHz, DMSO-*d*$_6$): δ 9.89 (s, 1H, pyrazole-NH), 9.33 (s, 1H, Ar-NH), 7.91 (d, *J* = 9.3 Hz, 1H, Ar-H), 7.40-7.38 (m, 2H, Ar-H), 3.63 (s, 3H, N-CH_3), 3.55 (s, 2H, O=C-CH_2-C=O), 3.45 (s, 3H, OCH_3), 2.33 (s, 3H, Ar-CH_3), 2.07 (s, 3H, pyrazole-CH_3), 1.99 (s, 3H, pyrazole-CH_3).

N[1]-(4-(1,1,1,3,3,3-Hexafluoro-2-methoxypropan-2-yl)-2-methoxyphenyl)-*N*[3]-(1,3,5-trimethyl-1H-pyrazol-4-yl)malonamide (intermediate **6-3**): Light yellow solid; m.p. 71-72 °C; yield 85.3%; ^{1}H NMR (300 MHz, DMSO-d_6): δ 10.06 (s, 1H, pyrazole-NH), 9.38 (s, 1H, Ar-NH), 8.37 (d, *J* = 8.6 Hz, 1H, Ar-H), 7.15 (d, *J* = 8.5 Hz, 1H, Ar-H), 7.07 (s, 1H, Ar-H), 3.90 (s, 3H, Ar-OCH_3), 3.63 (s, 3H, N-CH_3), 3.61 (s, 2H, O=C-CH_2-C=O), 3.47 (s, 3H, OCH_3), 2.07 (s, 3H, pyrazole-CH_3), 1.98 (s, 3H, pyrazole-CH_3).

N[1]-(4-(1,1,1,3,3,3-Hexafluoro-2-methoxypropan-2-yl)-3-isopropylphenyl)-*N*[3]-(1,3,5-trimethyl-1H-pyrazol-4-yl)malonamide (intermediate **6-4**): White solid; m.p. 56-57 °C; yield 84.8%; ^{1}H NMR (300 MHz, DMSO-d_6): δ 9.86 (s, 1H, pyrazole-NH), 9.41 (s, 1H, Ar-NH), 8.02 (d, *J* = 2.0 Hz, 1H, Ar-H), 7.39 (d, *J* = 8.4 Hz, 1H, Ar-H), 7.21 (dd, *J* = 8.5, 2.0 Hz, 1H, Ar-H), 3.61 (s, 3H, N-CH_3), 3.56 (s, 2H, O=C-CH_2-C=O), 3.42 (s, 3H, OCH_3), 2.90 (hept, *J* = 6.9 Hz, 1H, $CH(CH_3)_2$), 2.04 (s, 3H, pyrazole-CH_3), 1.96 (s, 3H, pyrazole-CH_3), 1.20 (d, *J* = 6.9 Hz, 6H, $CH(CH_3)_2$).

Table S1. The structures of intermediate **3**, **6** and all target compounds

Compd.	Structure	Compd.	Structure
Intermediate **3-1**	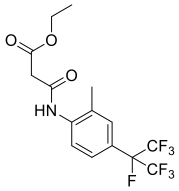	Intermediate **3-2**	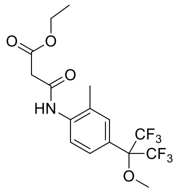
Intermediate **3-3**	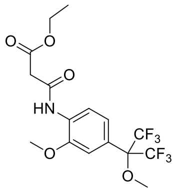	Intermediate **3-4**	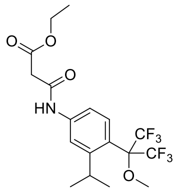
Intermediate **6-1**	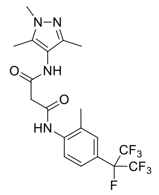	Intermediate **6-2**	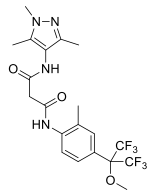
Intermediate **6-3**	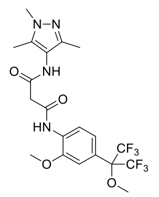	Intermediate **6-4**	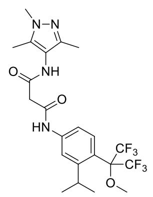

Compound **8a**

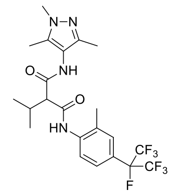

Compound **8b**

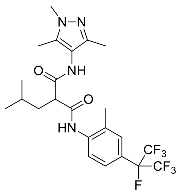

Compound **8c**

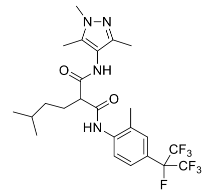

Compound **8d**

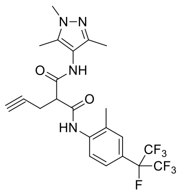

Compound **8e**

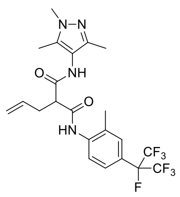

Compound **8f**

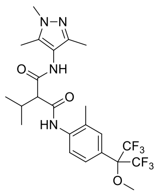

Compound **8g**

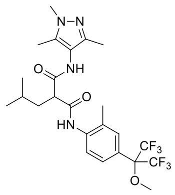

Compound **8h**

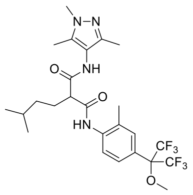

Compound **8i**

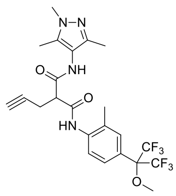

Compound **8j**

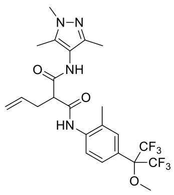

Compound **8k**

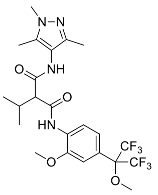

Compound **8l**

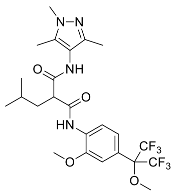

Compound **8m**

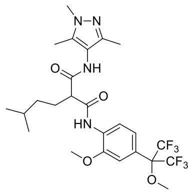

Compound **8n**

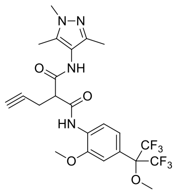

Compound **8o**

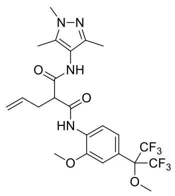

Compound **8p**

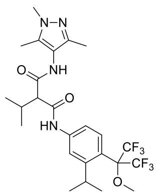

Compound **8q**	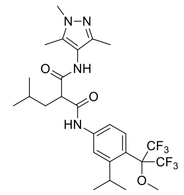	Compound **8r**	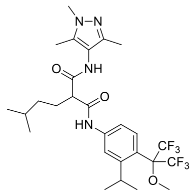
Compound **8s**	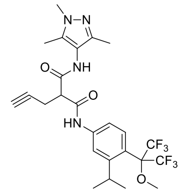	Compound **8t**	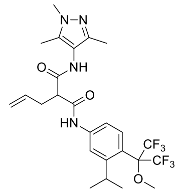

^{1}H NMR spectrums of intermediate **3**, **6** and all target compounds

Intermediate **3-1**

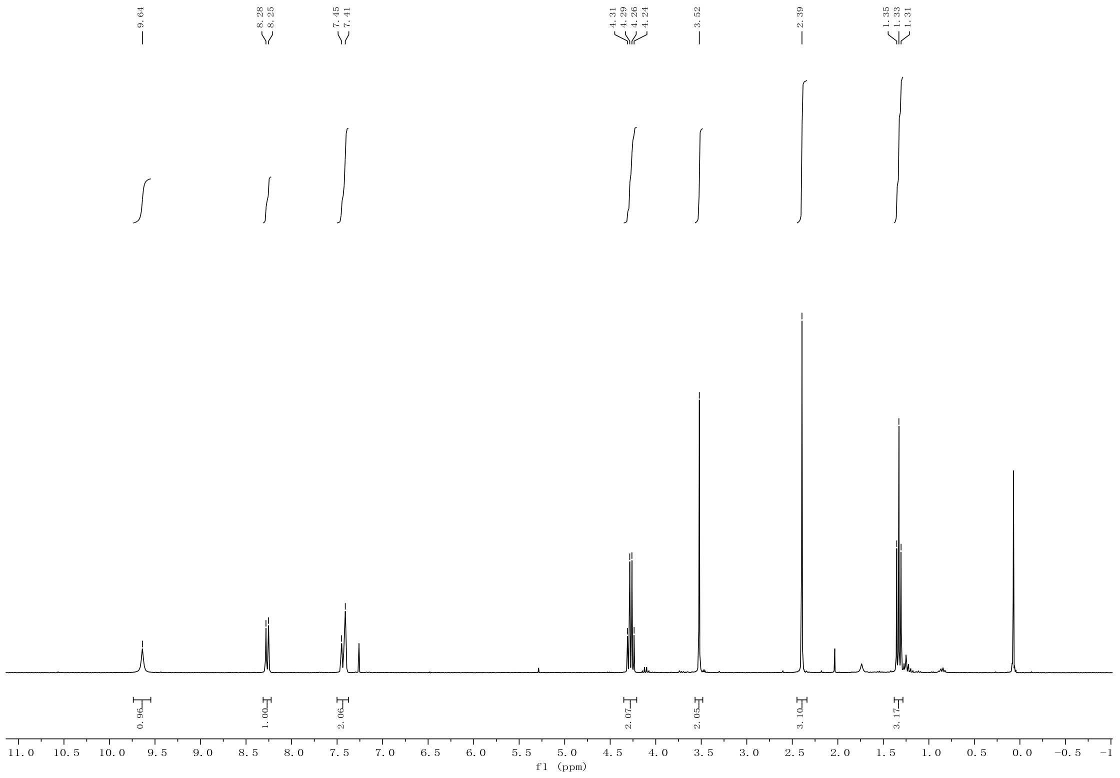

Intermediate **3-2**

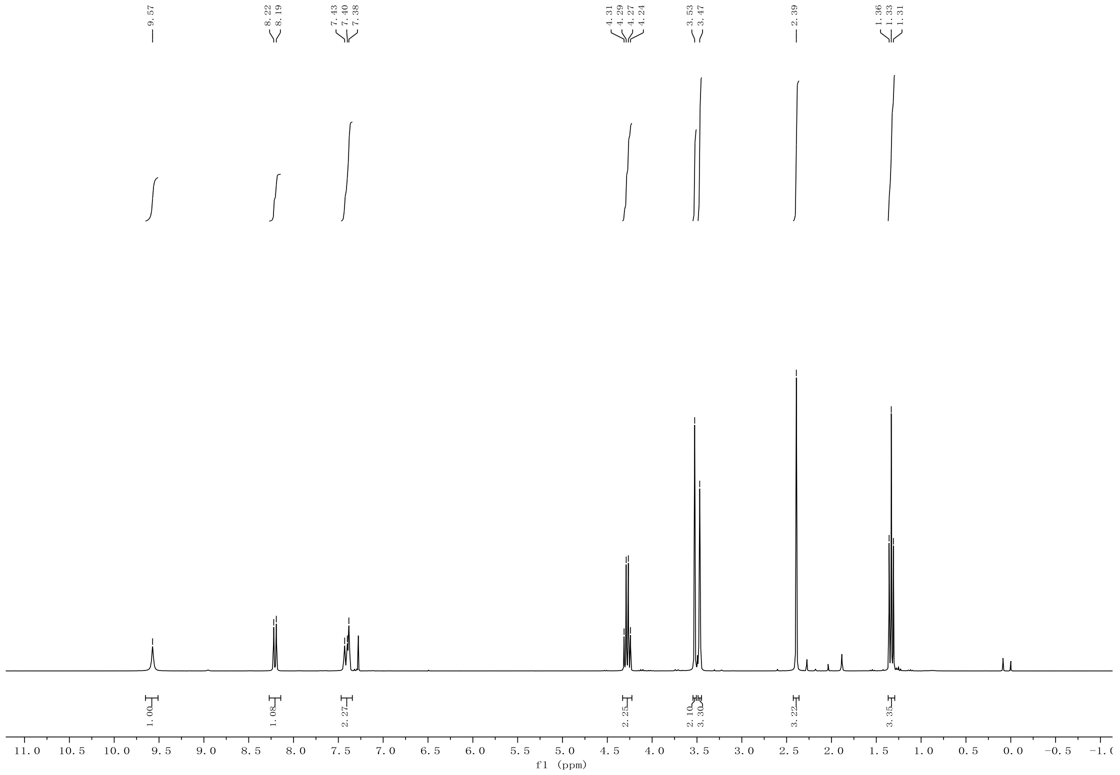

Intermediate **3-3**

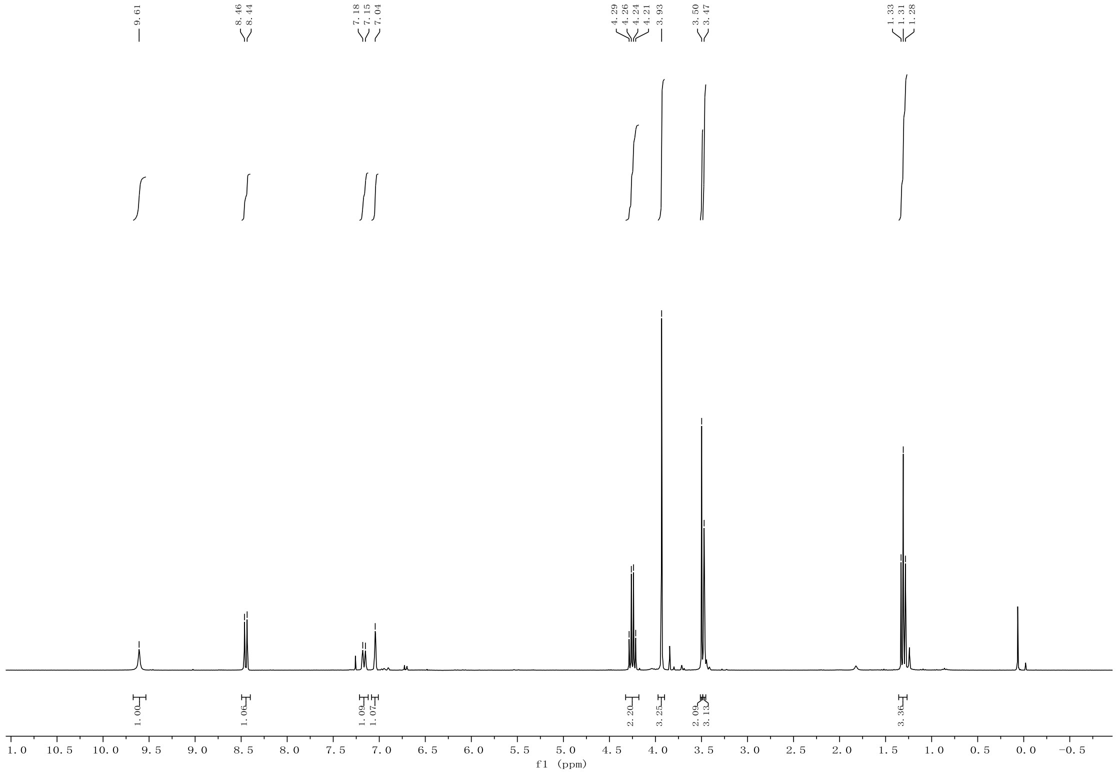

Intermediate **3-4**

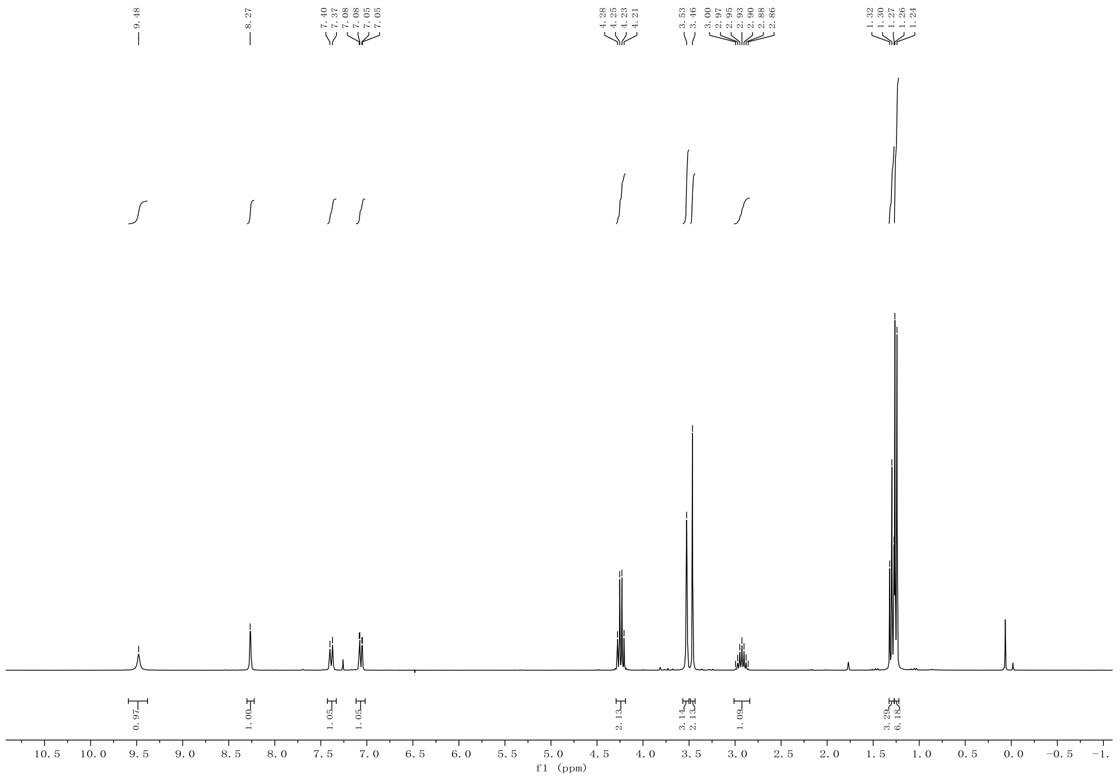

Intermediate **6-1**

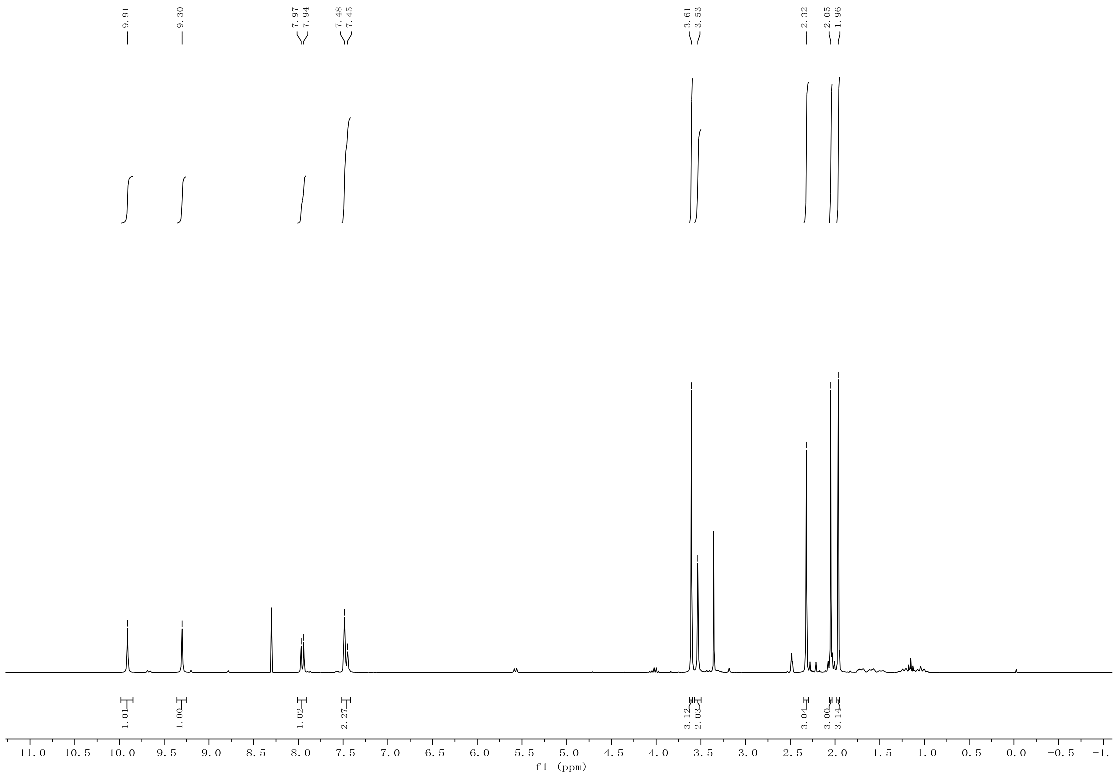

Intermediate **6-2**

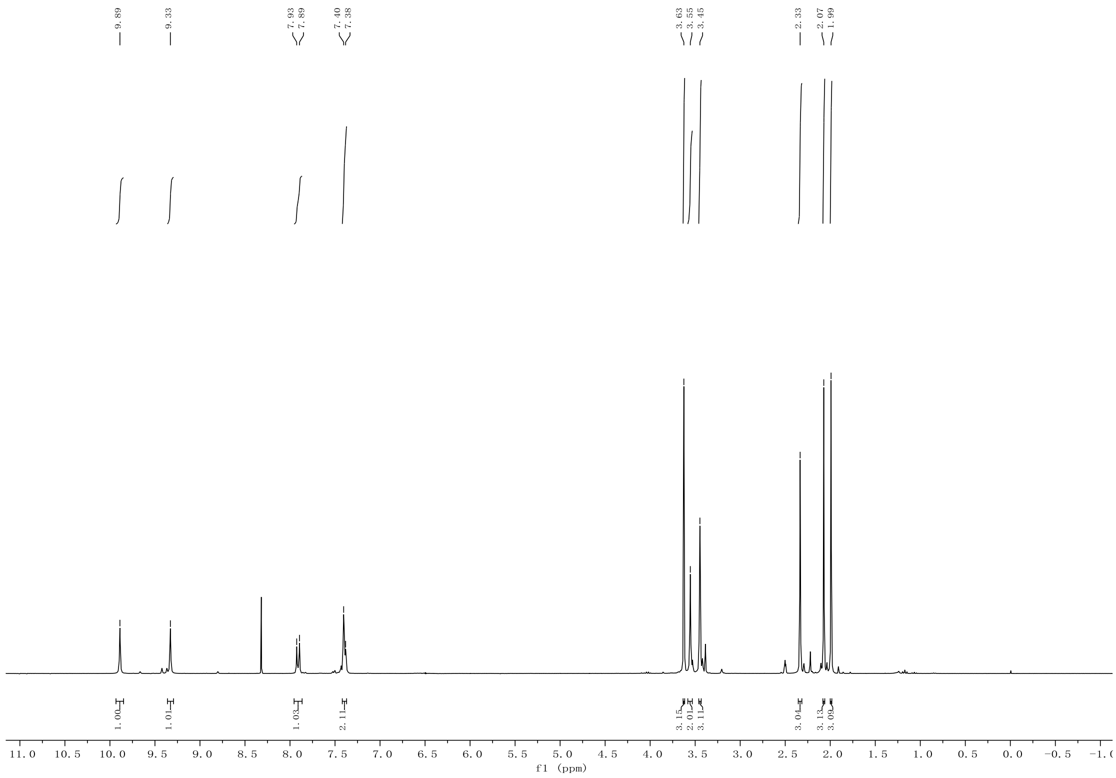

Intermediate **6-3**

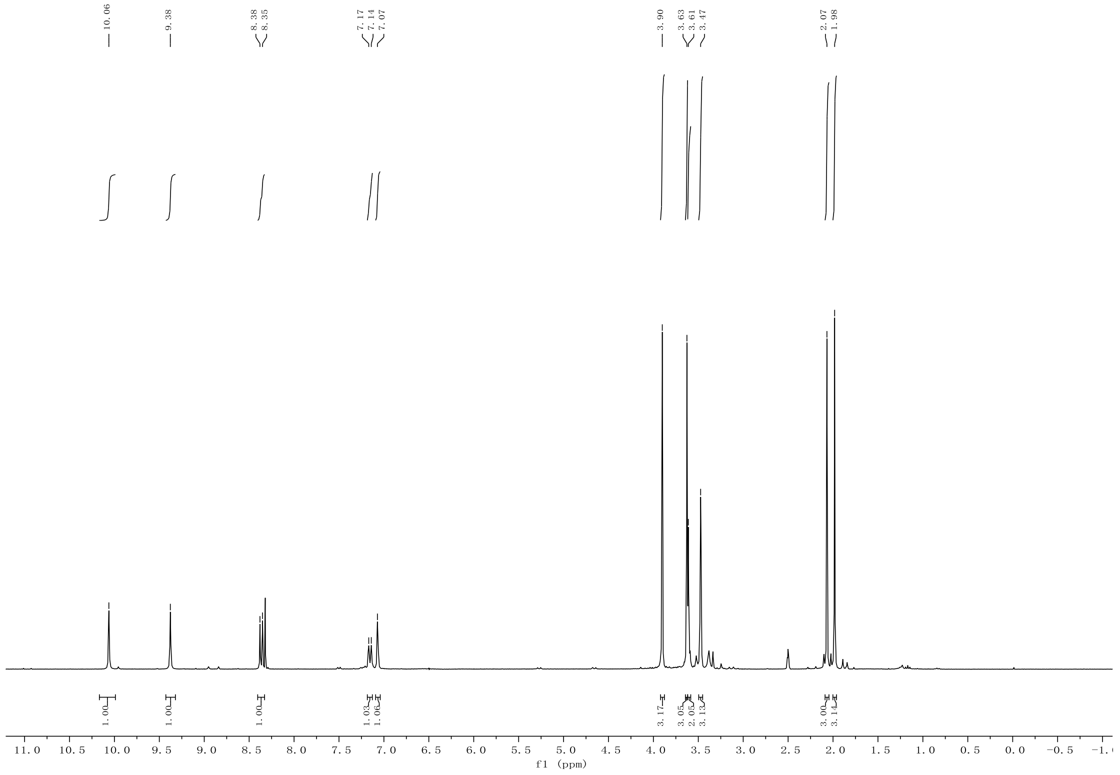

Intermediate **6-4**

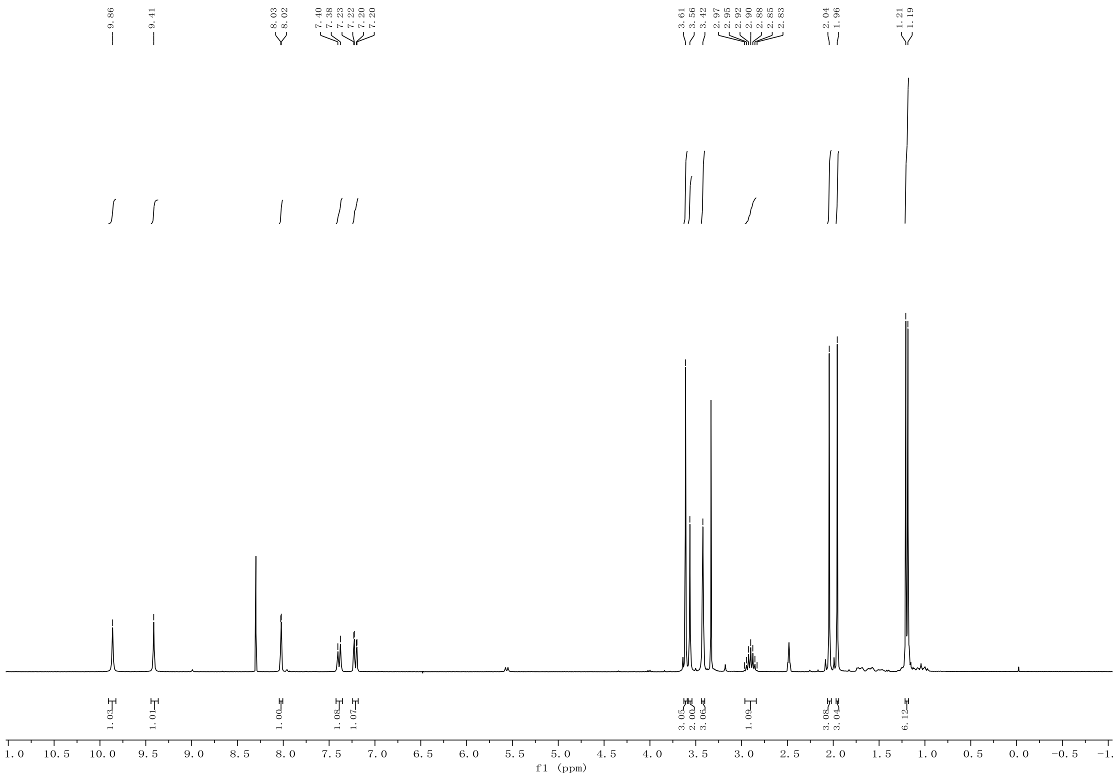

Compound **8a**

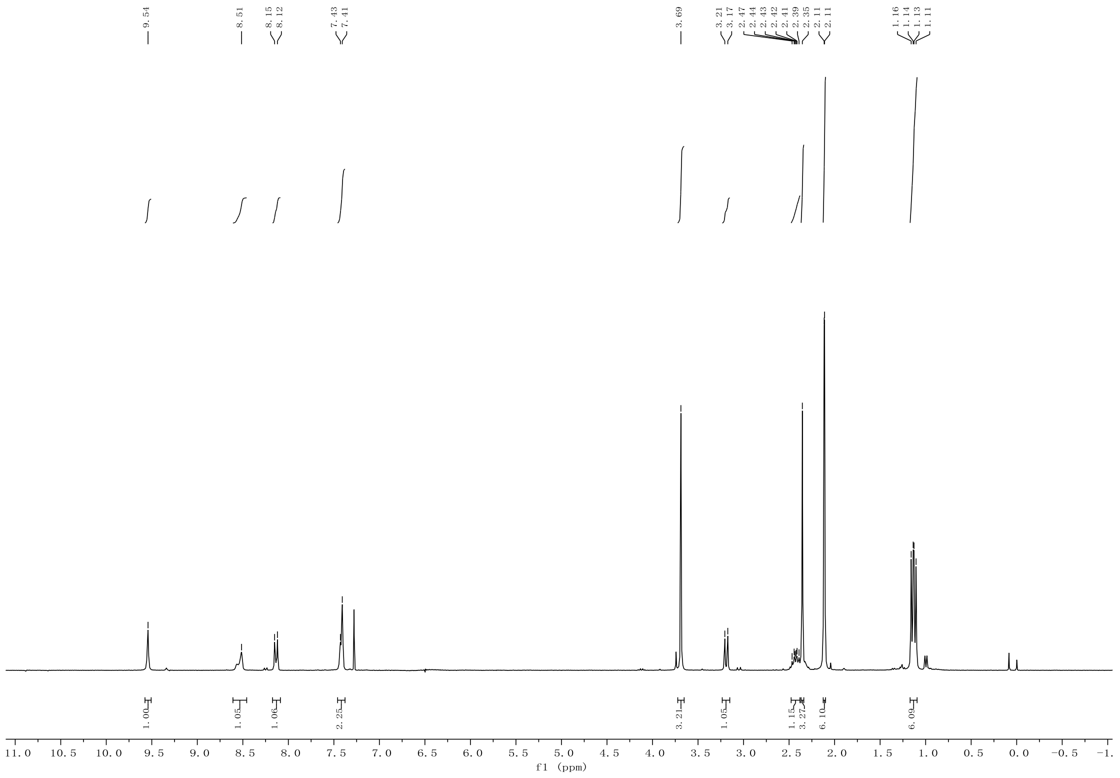

Compound **8b**

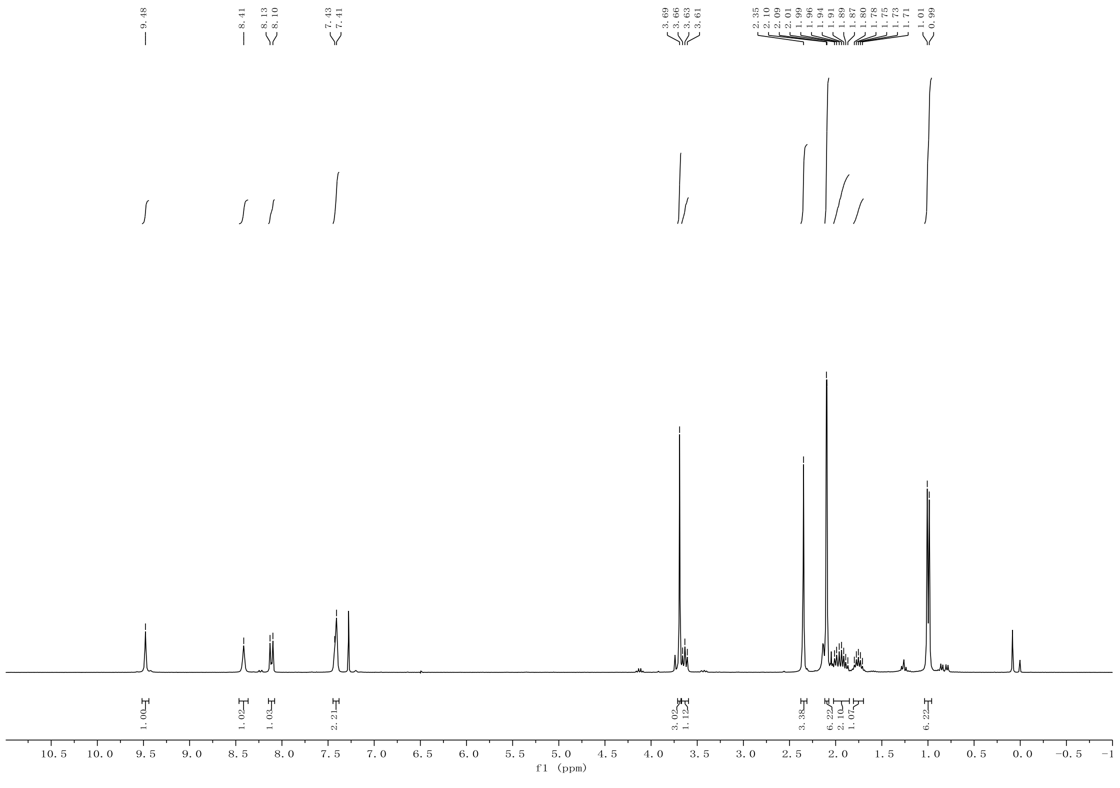

Compound **8c**

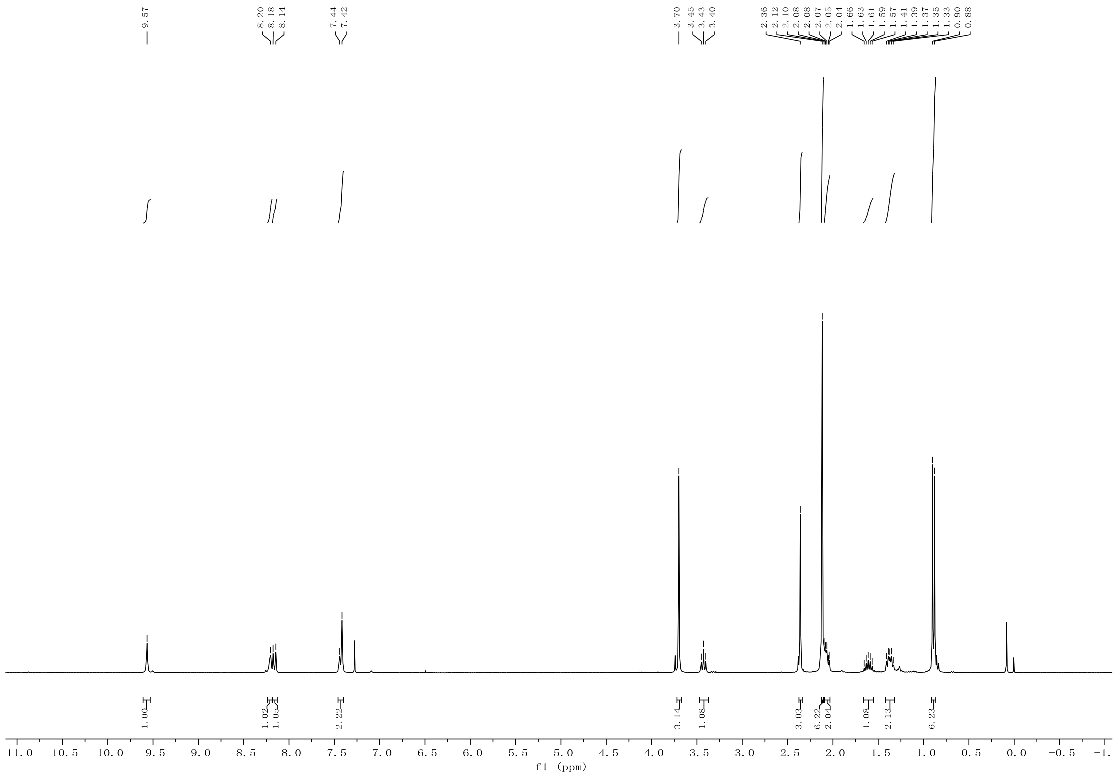

Compound **8d**

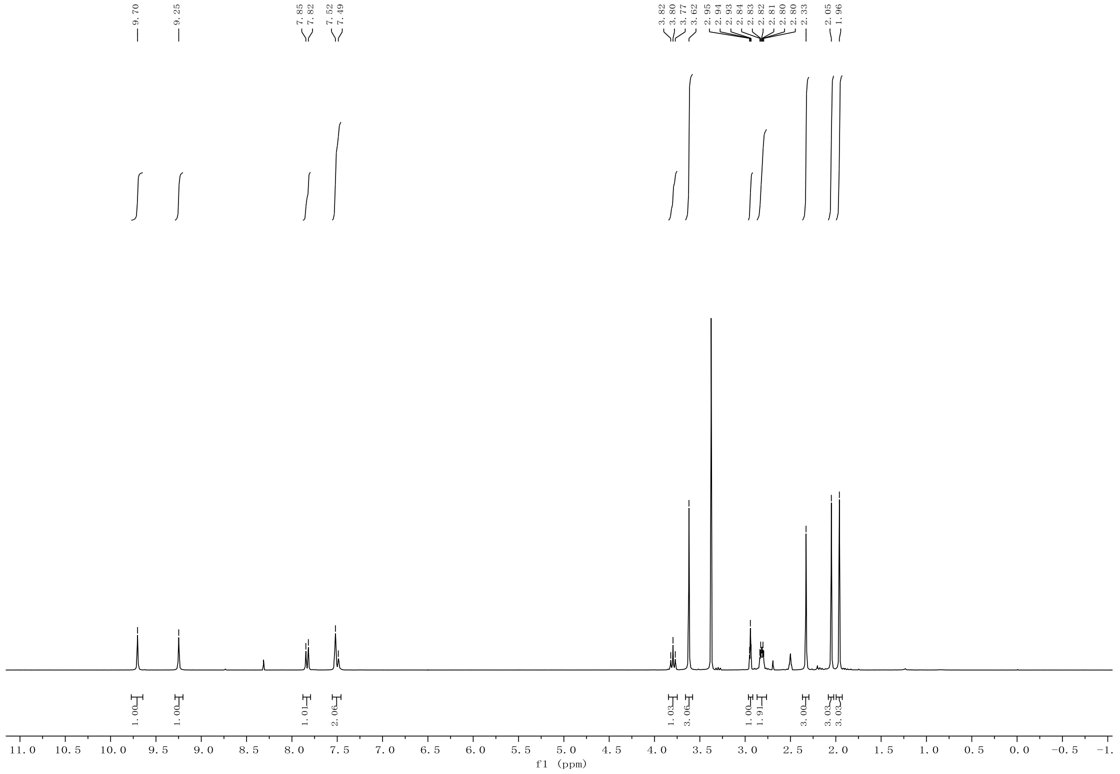

Compound **8e**

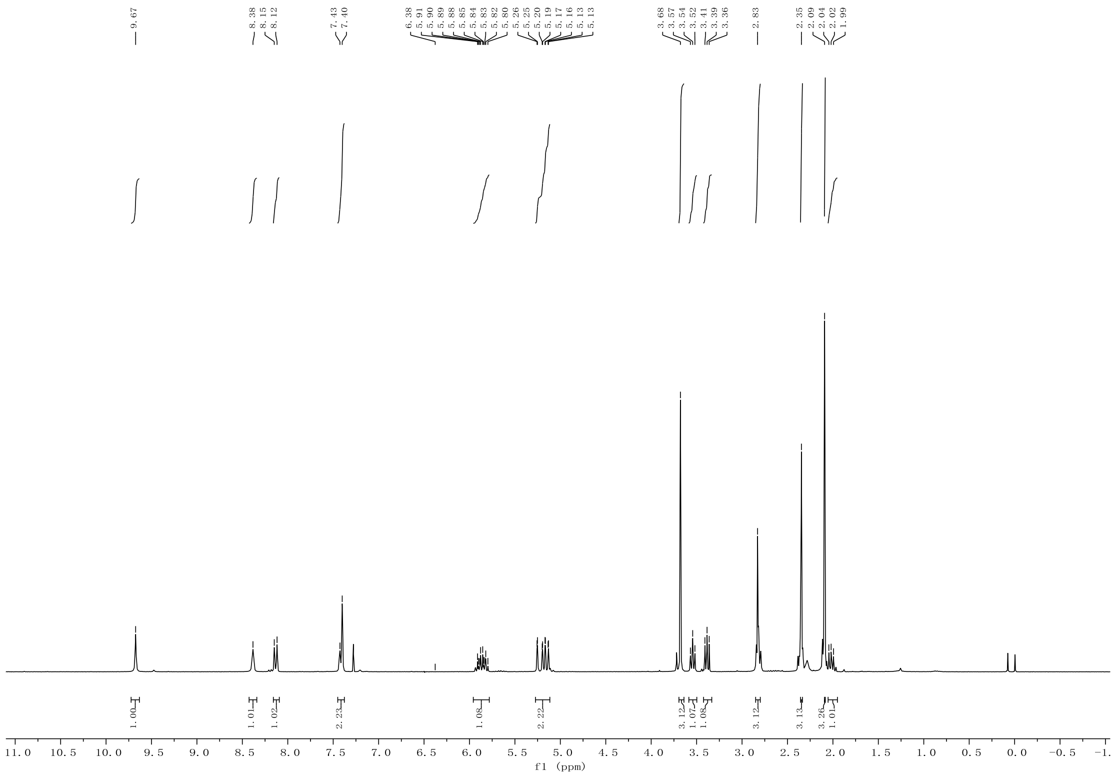

Compound **8f**

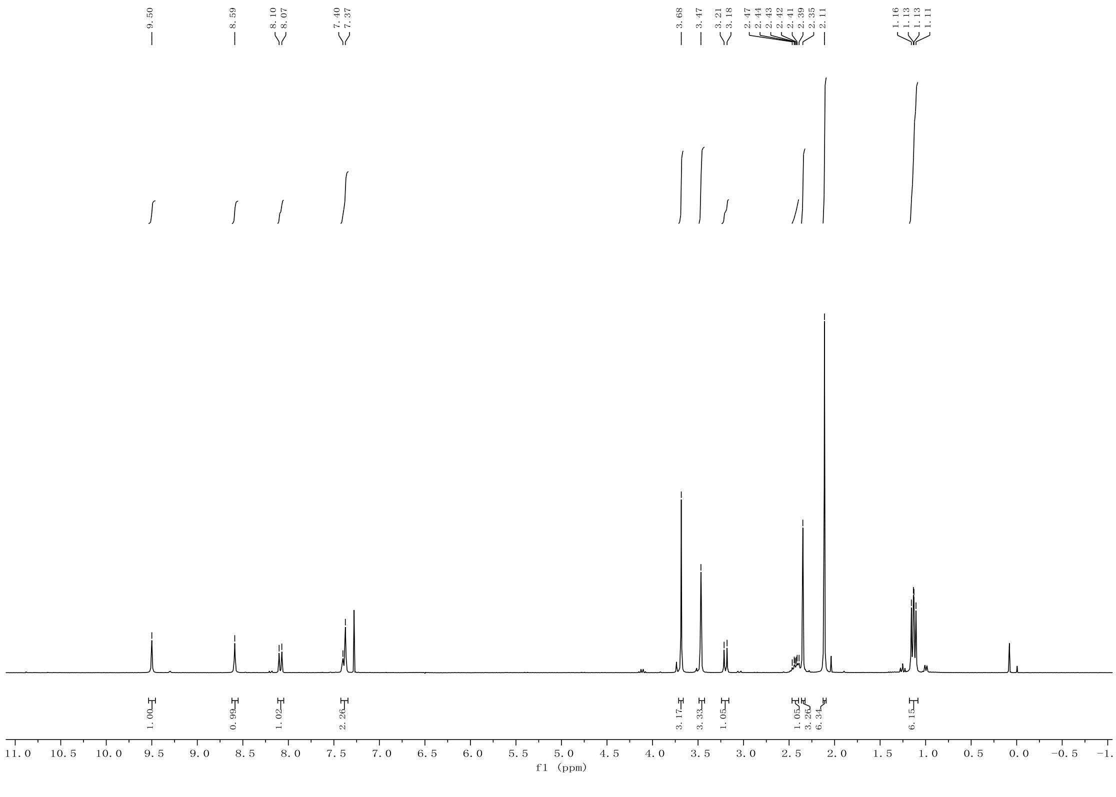

Compound **8g**

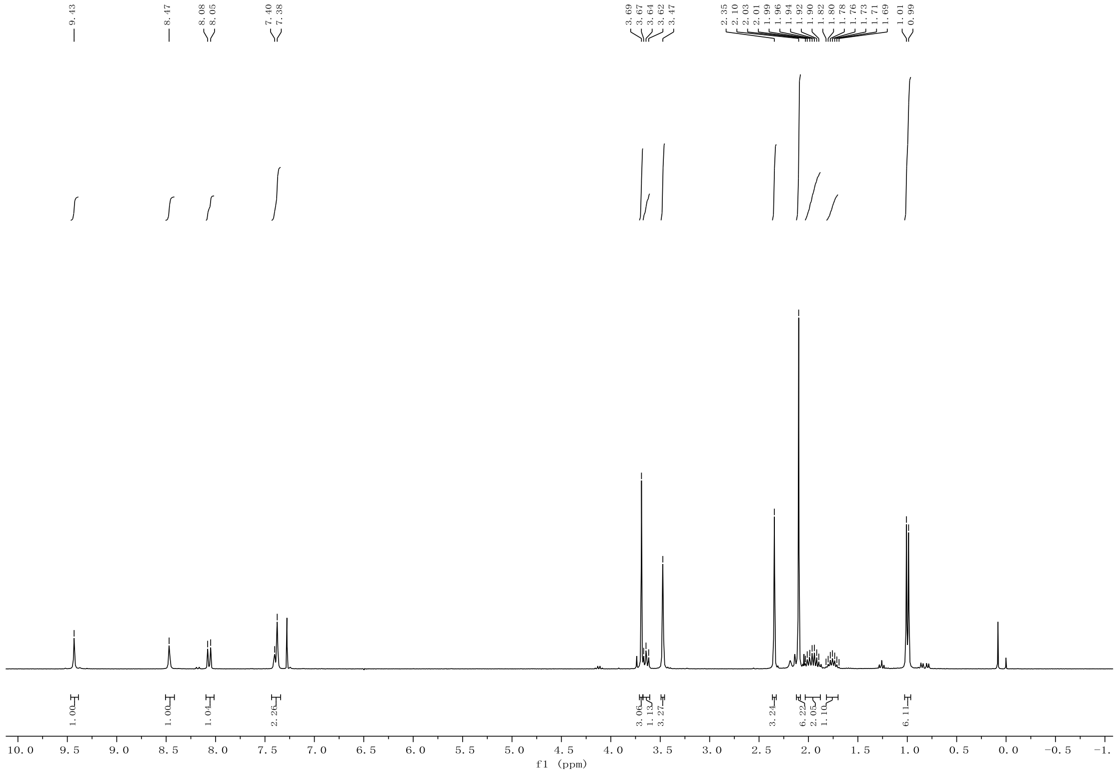

Compound **8h**

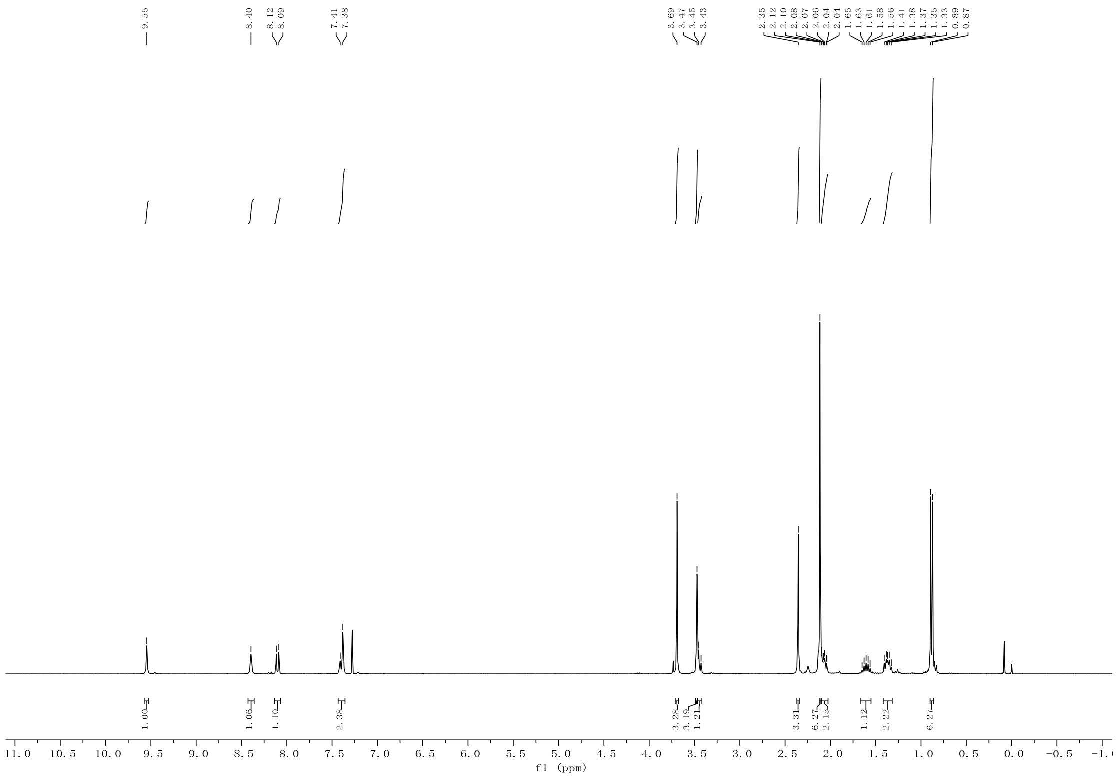

Compound **8i**

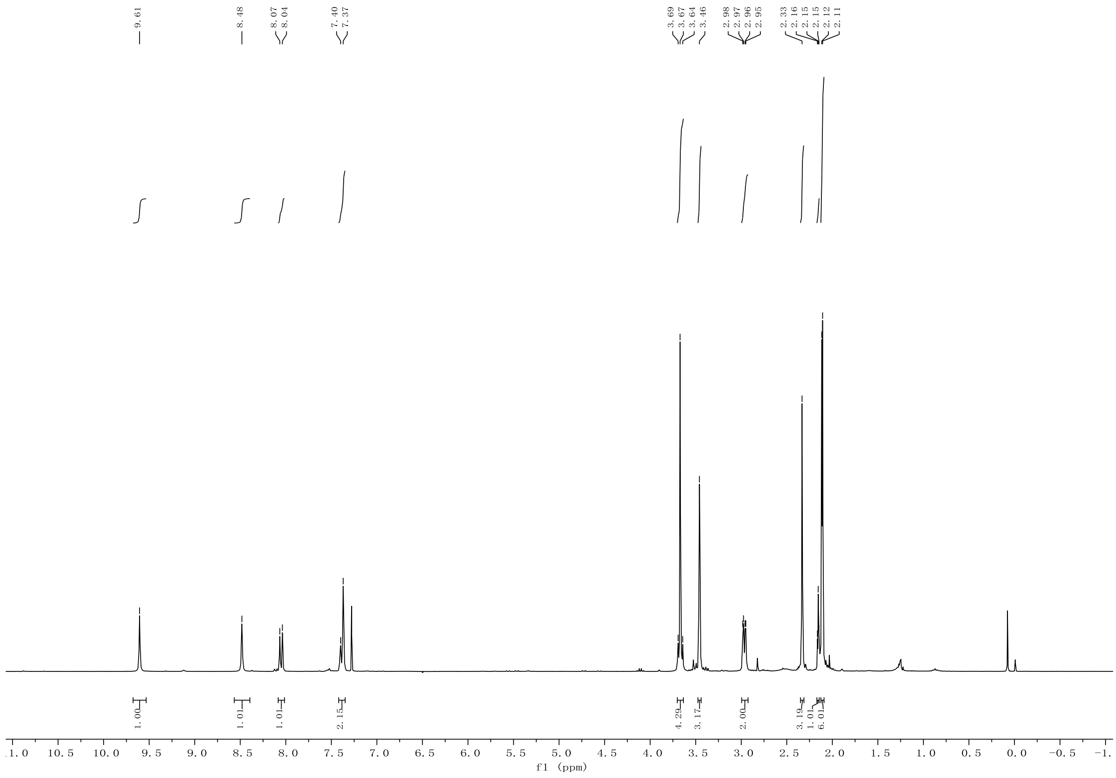

Compound **8j**

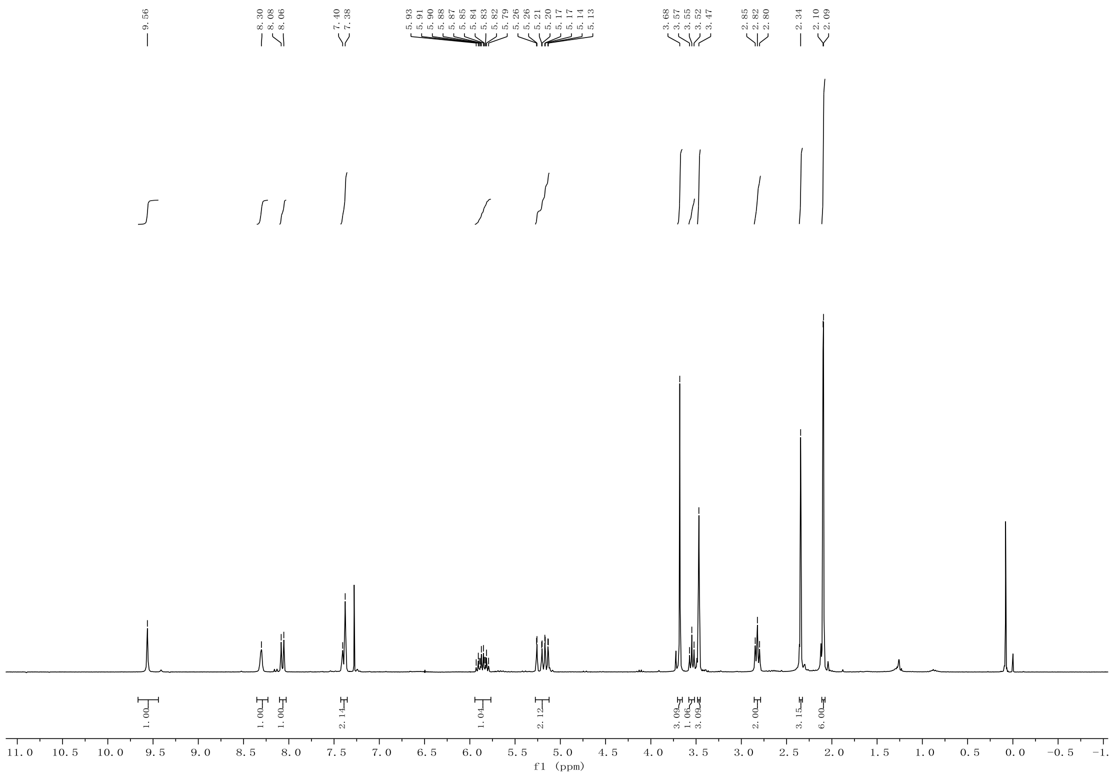

Compound **8k**

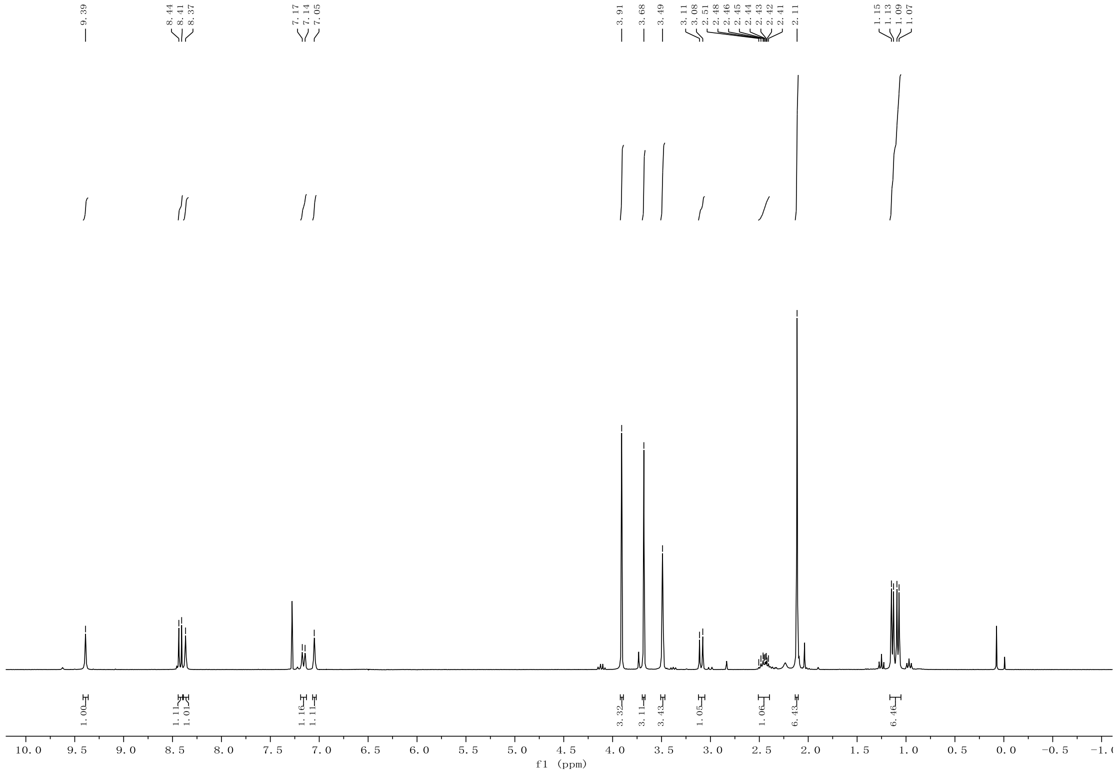

Compound **8l**

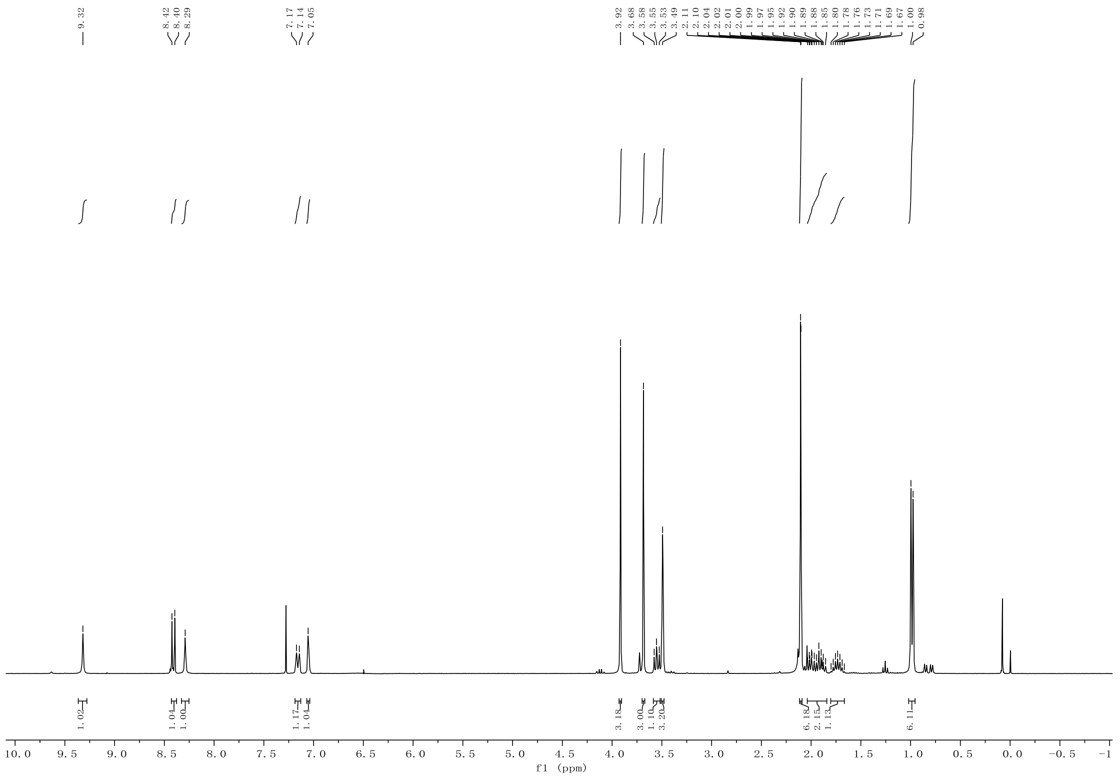

Compound **8m**

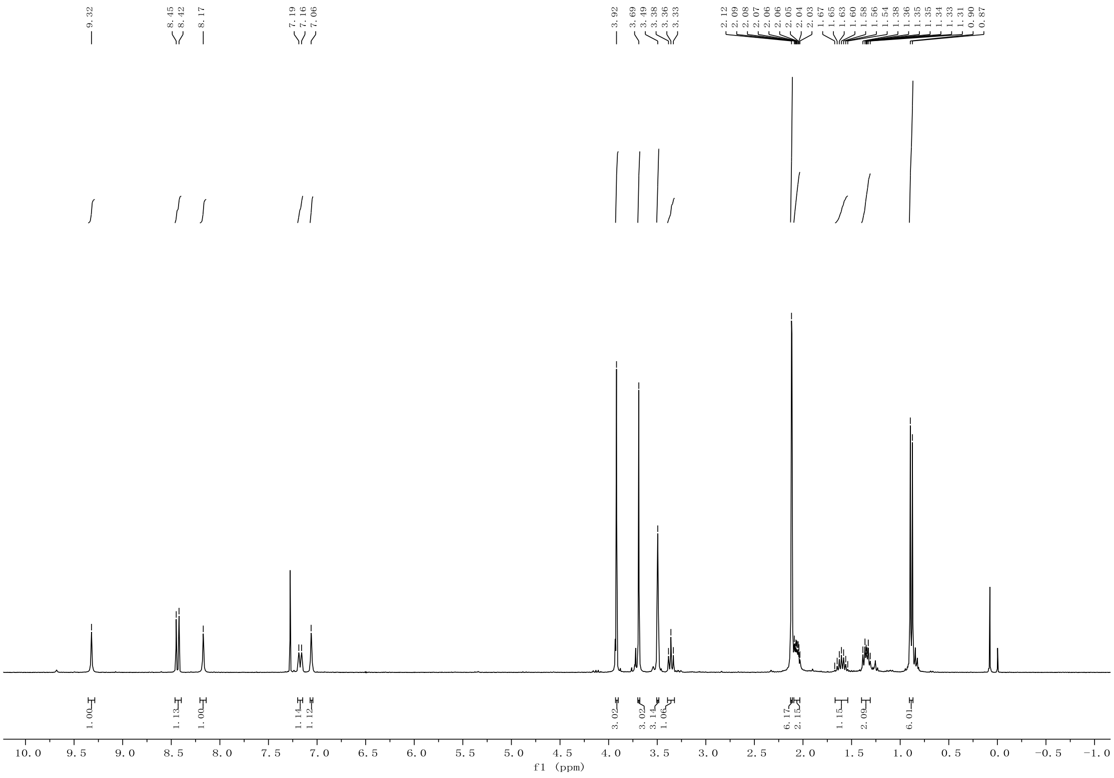

Compound **8n**

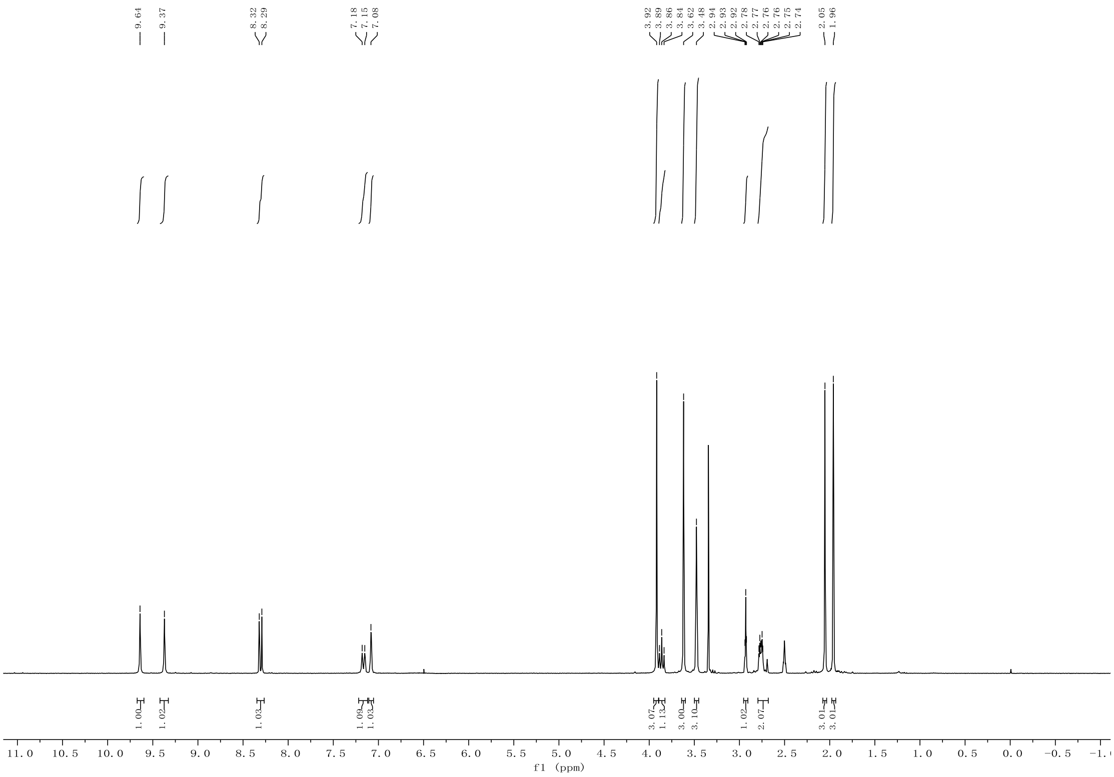

Compound **8o**

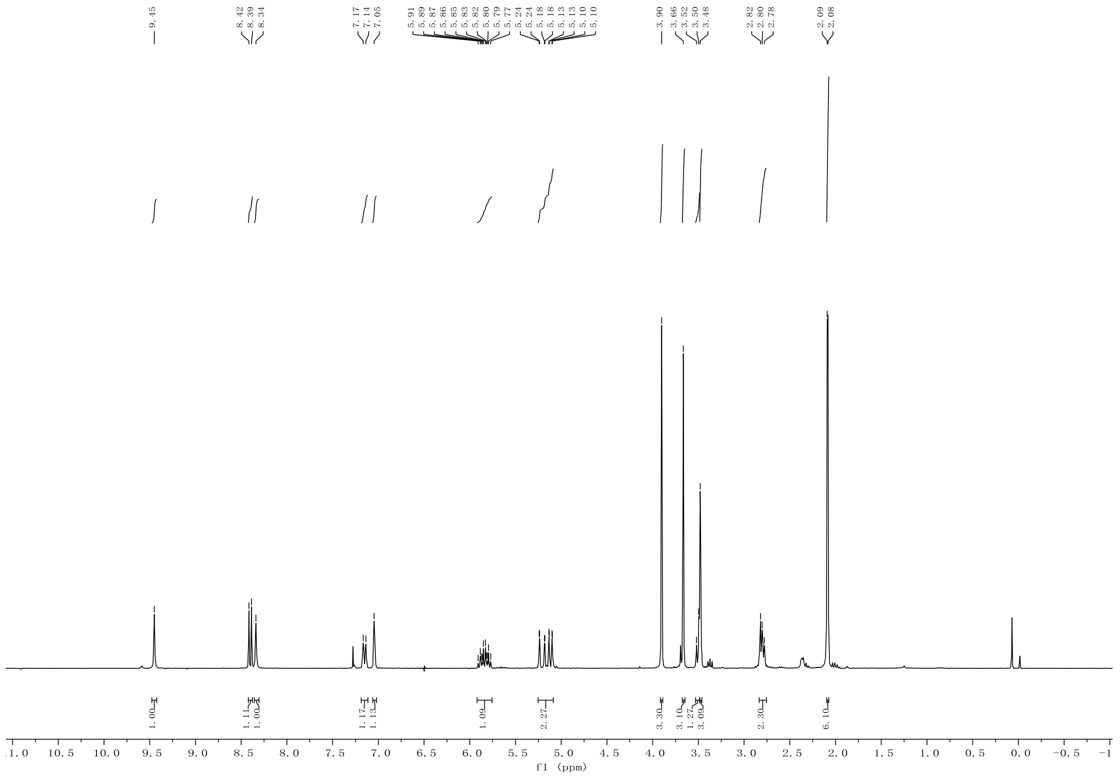

Compound **8p**

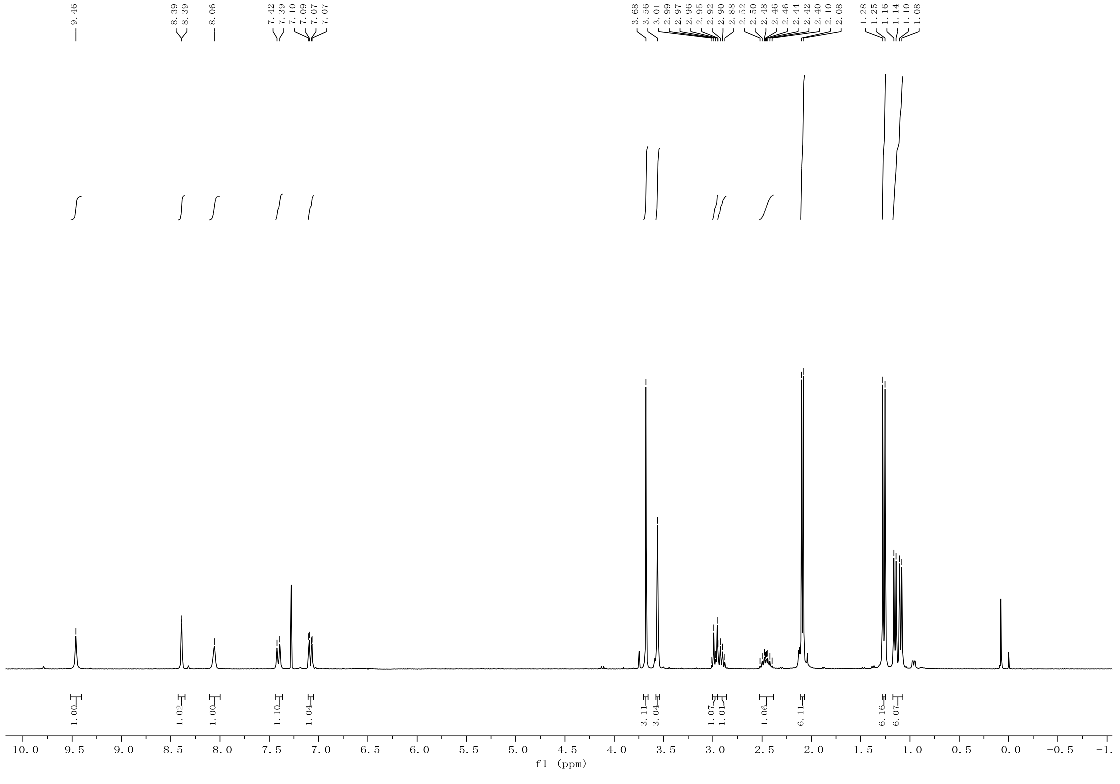

Compound **8q**

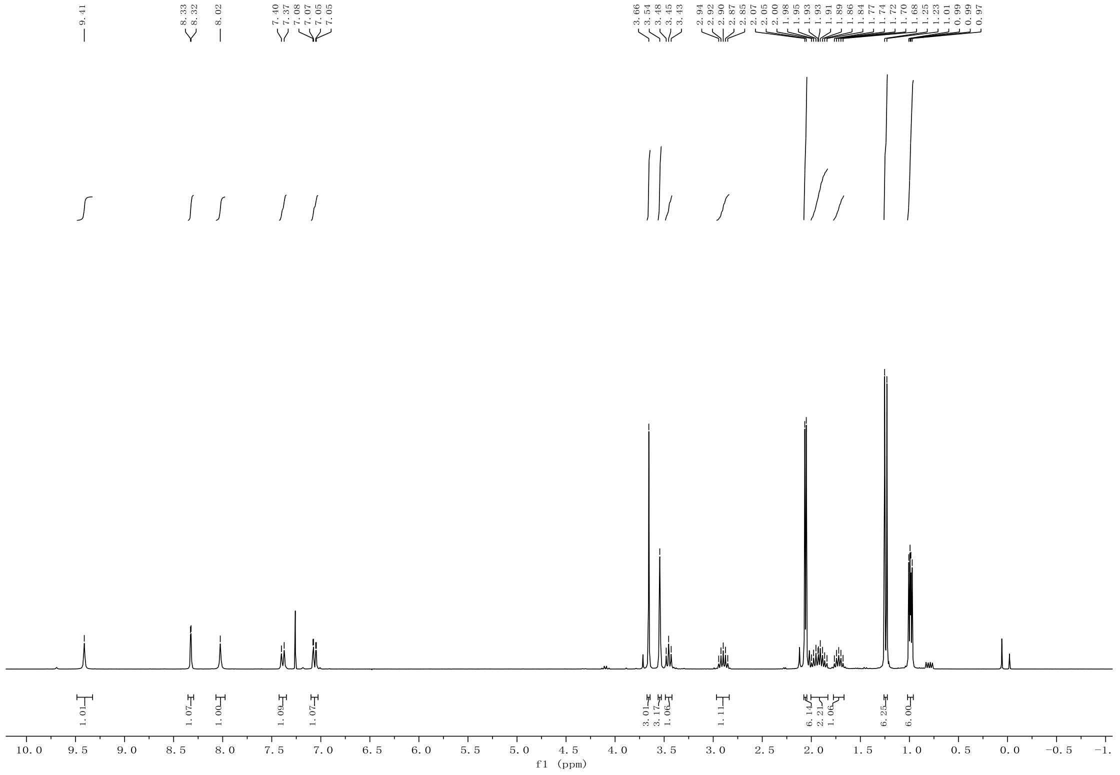

Compound **8r**

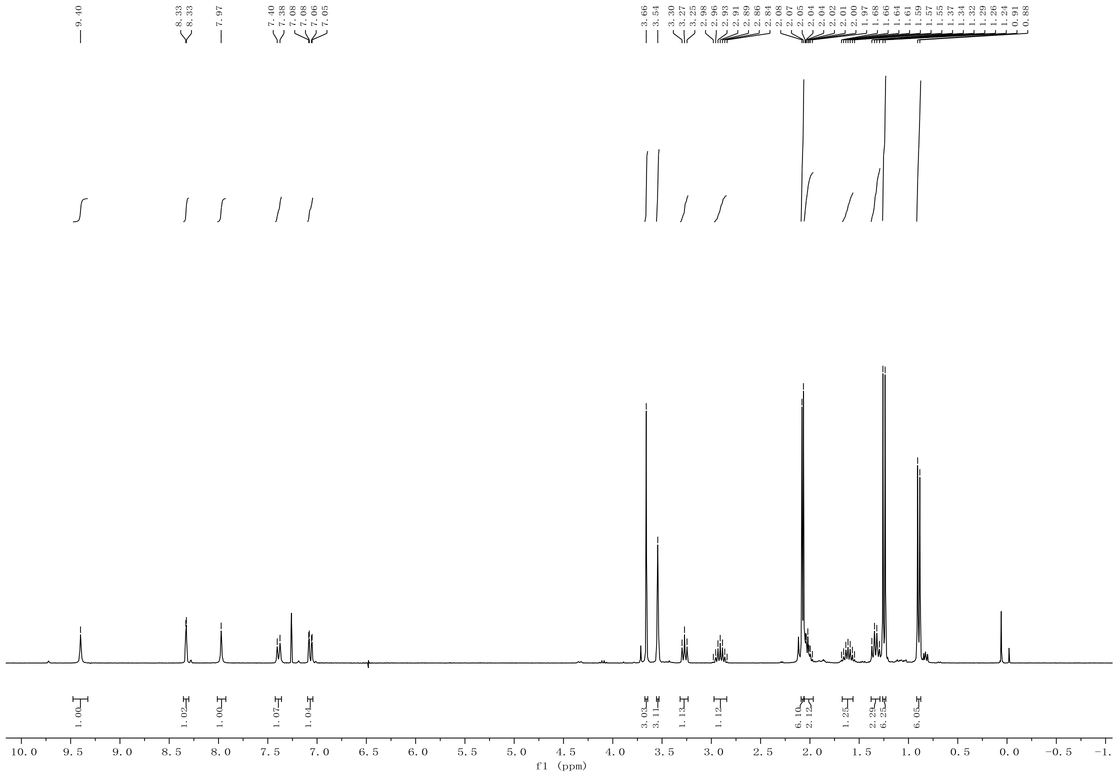

Compound **8s**

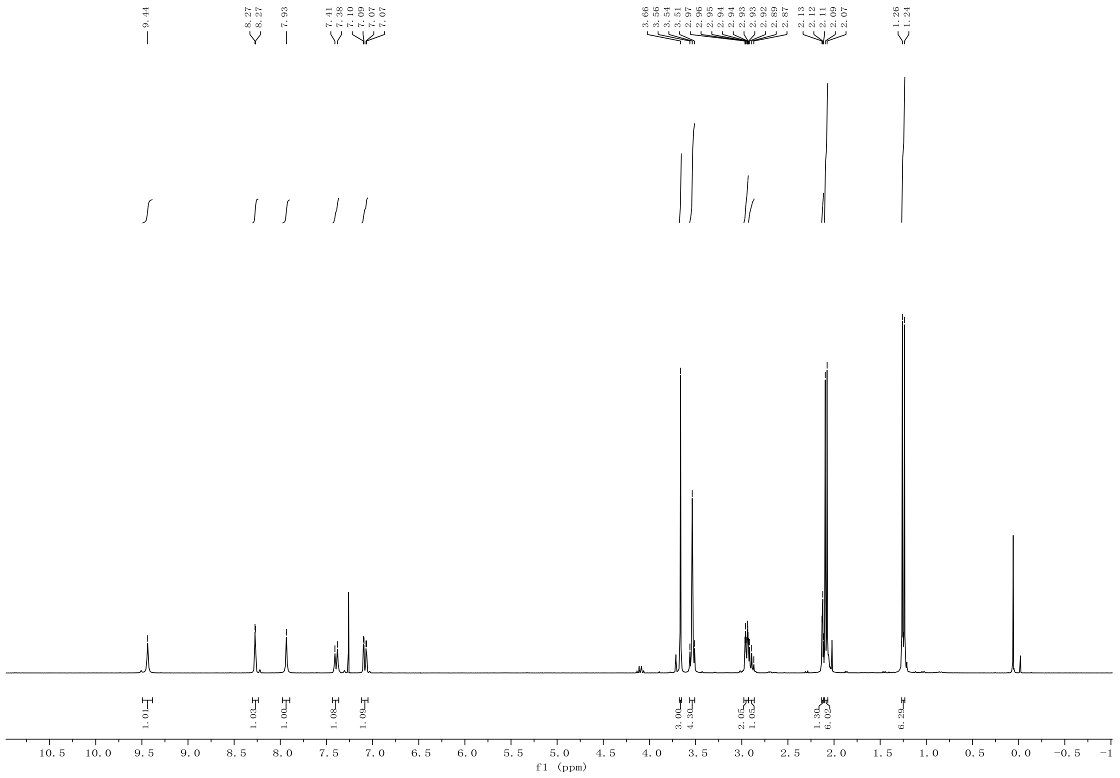

Compound **8t**

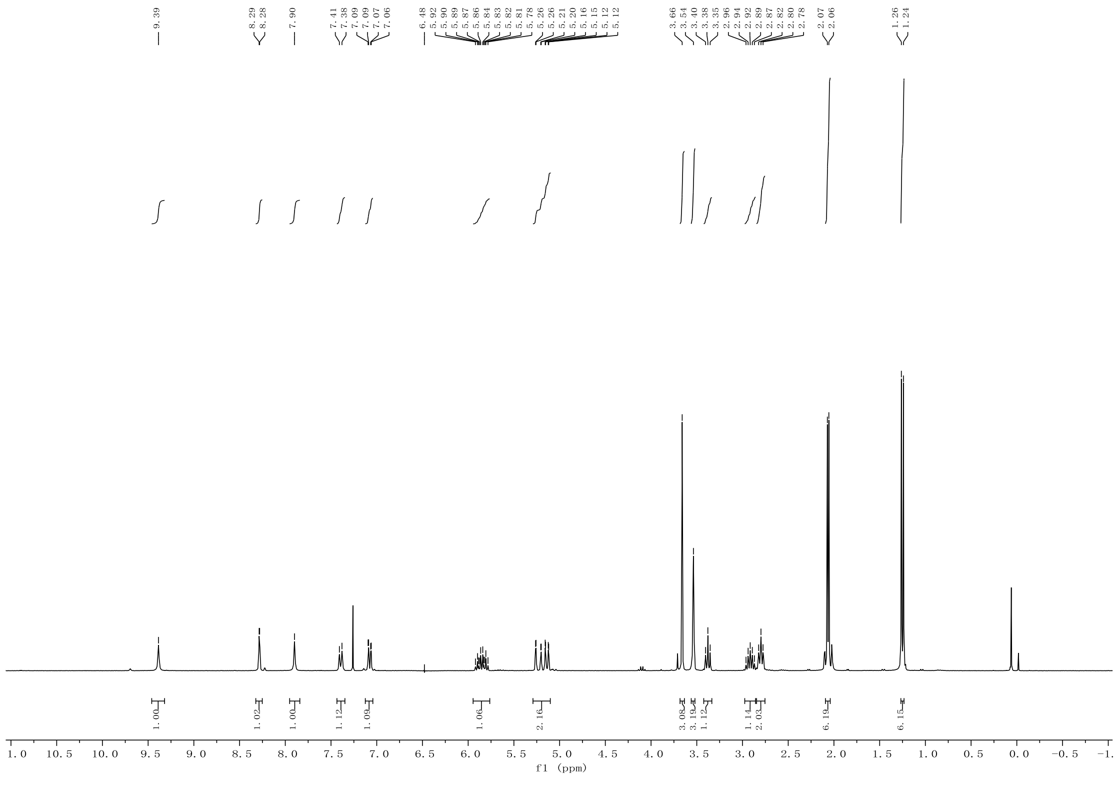

^{13}C NMR spectrums of all target compounds

Compound **8a**

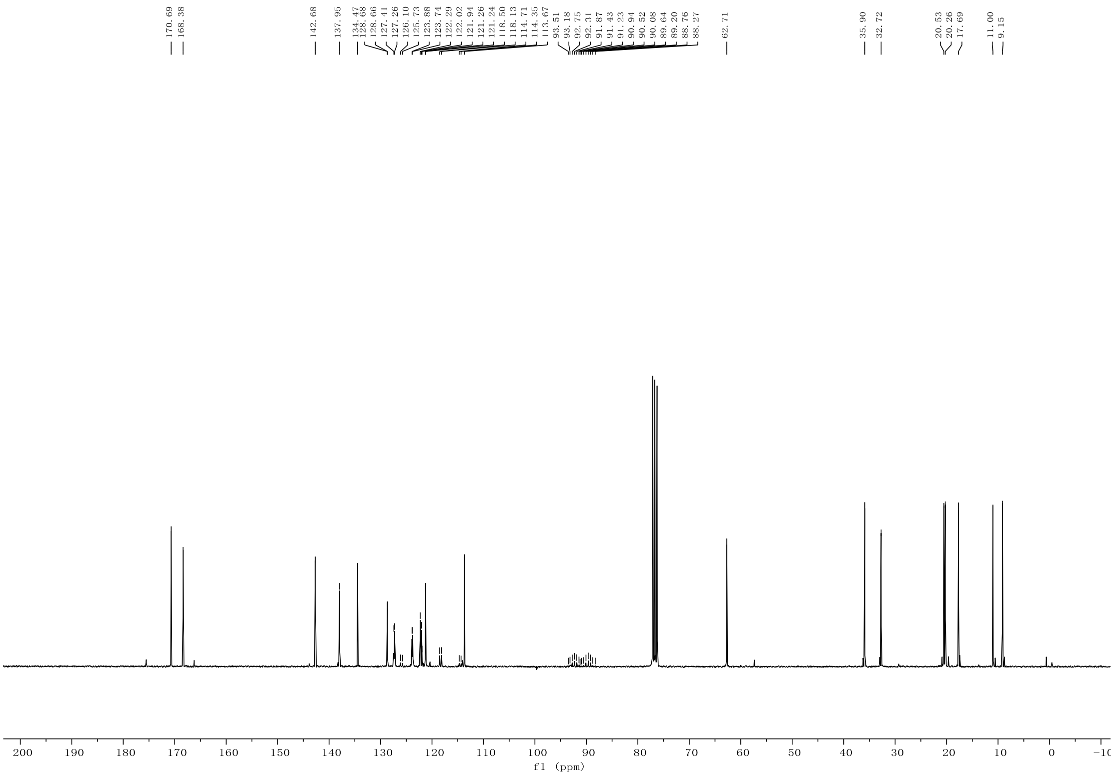

Compound **8b**

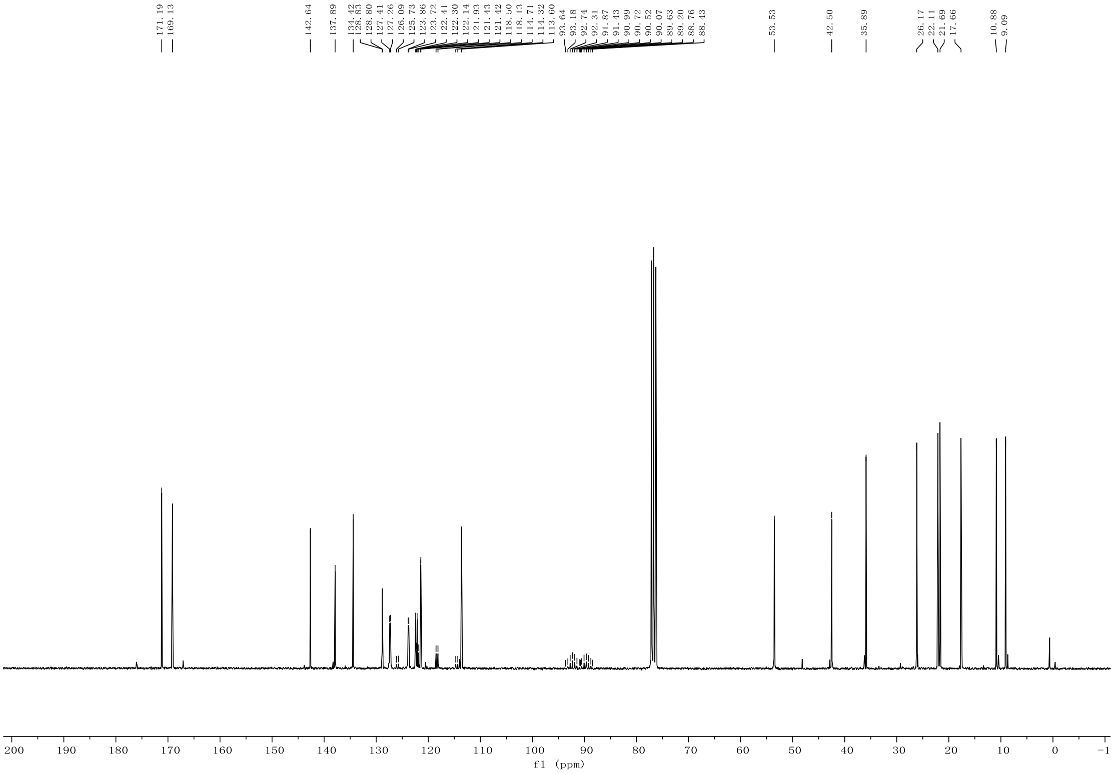

Compound **8c**

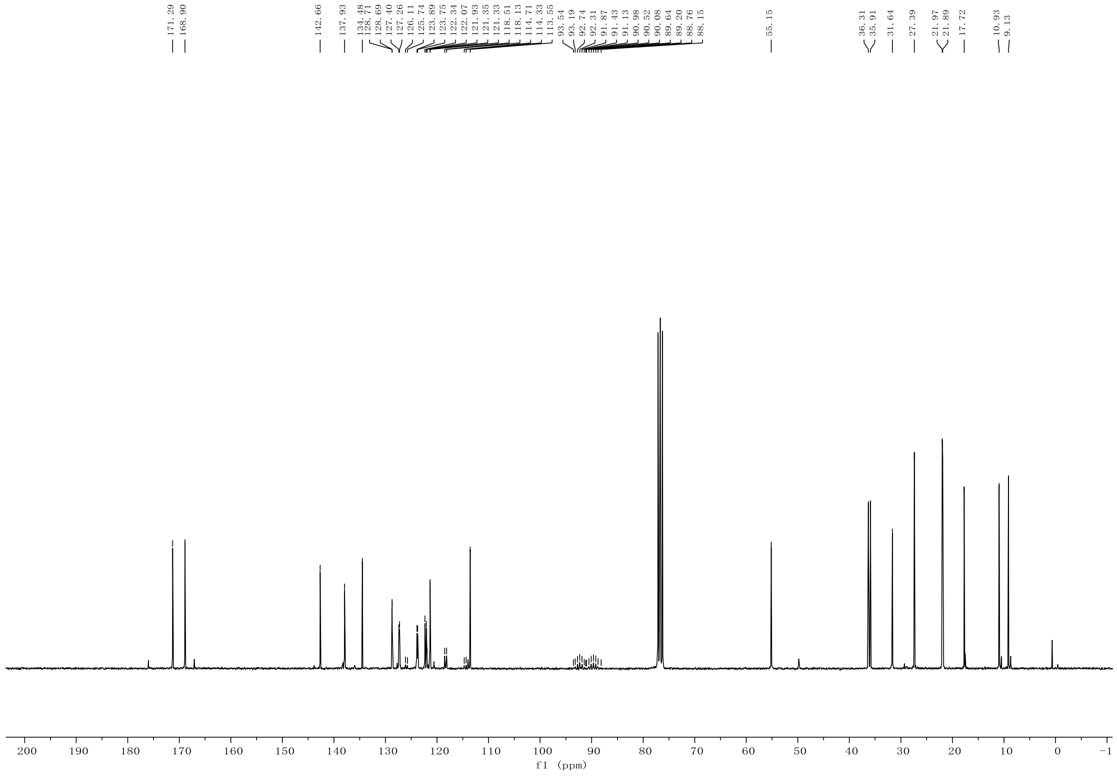

Compound **8d**

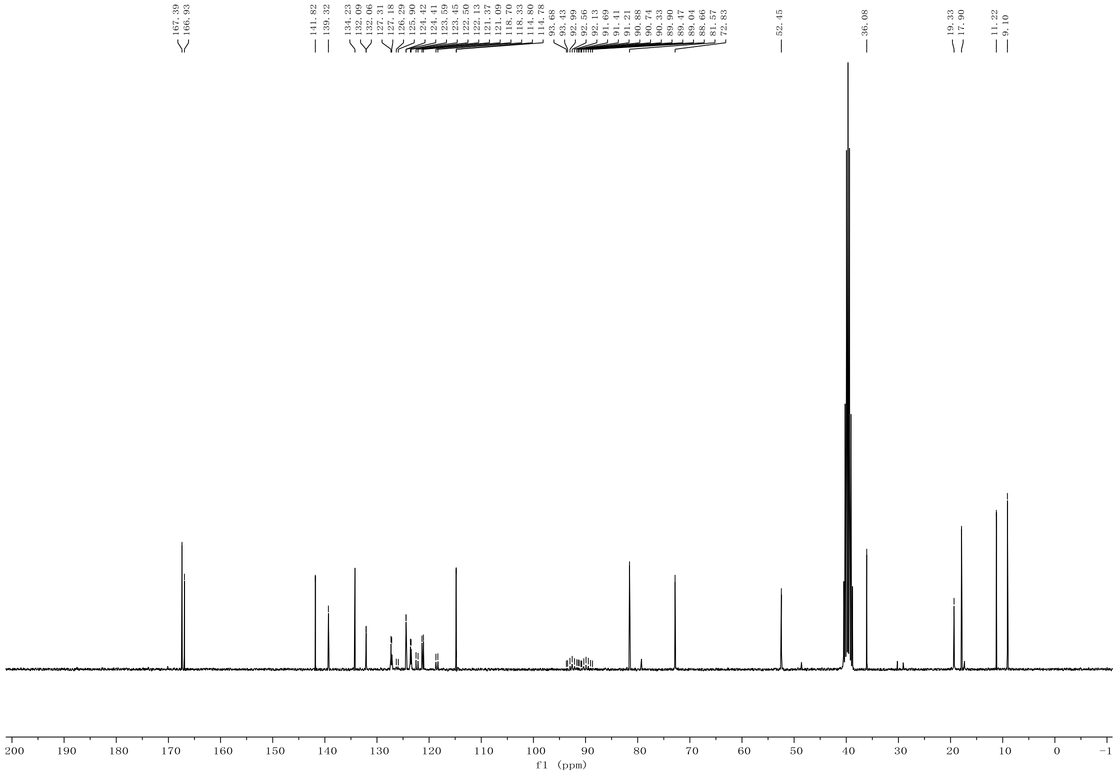

Compound **8e**

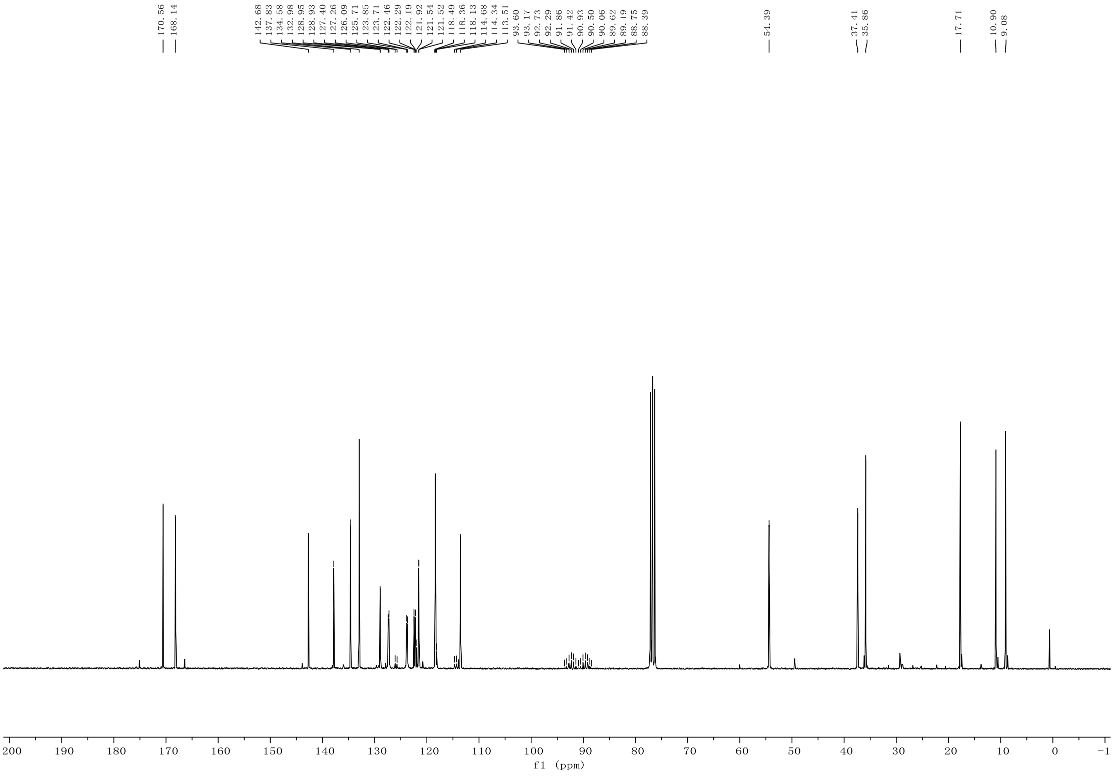

Compound **8f**

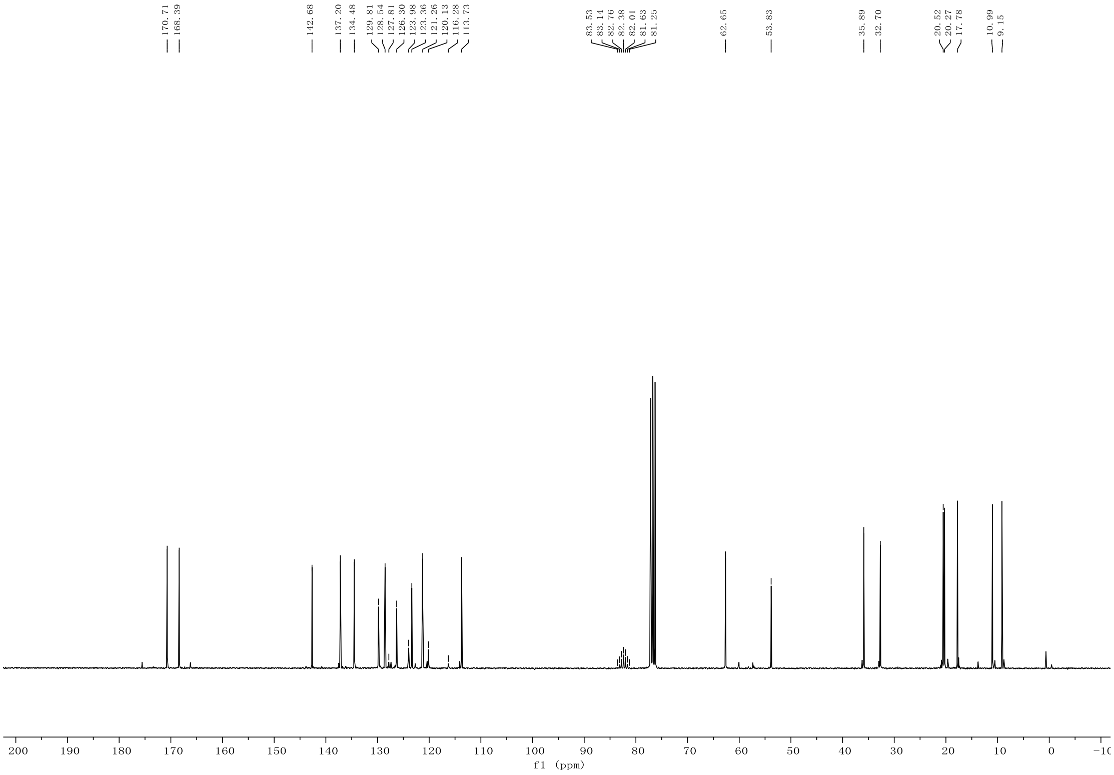

Compound **8g**

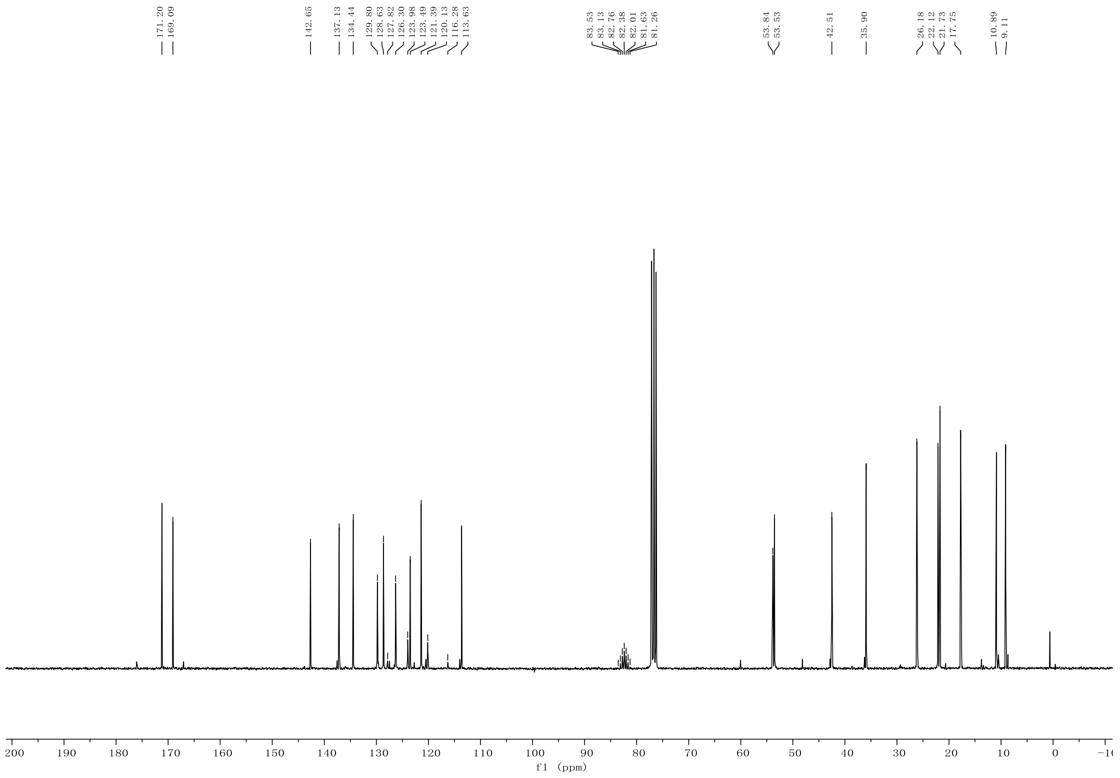

Compound **8h**

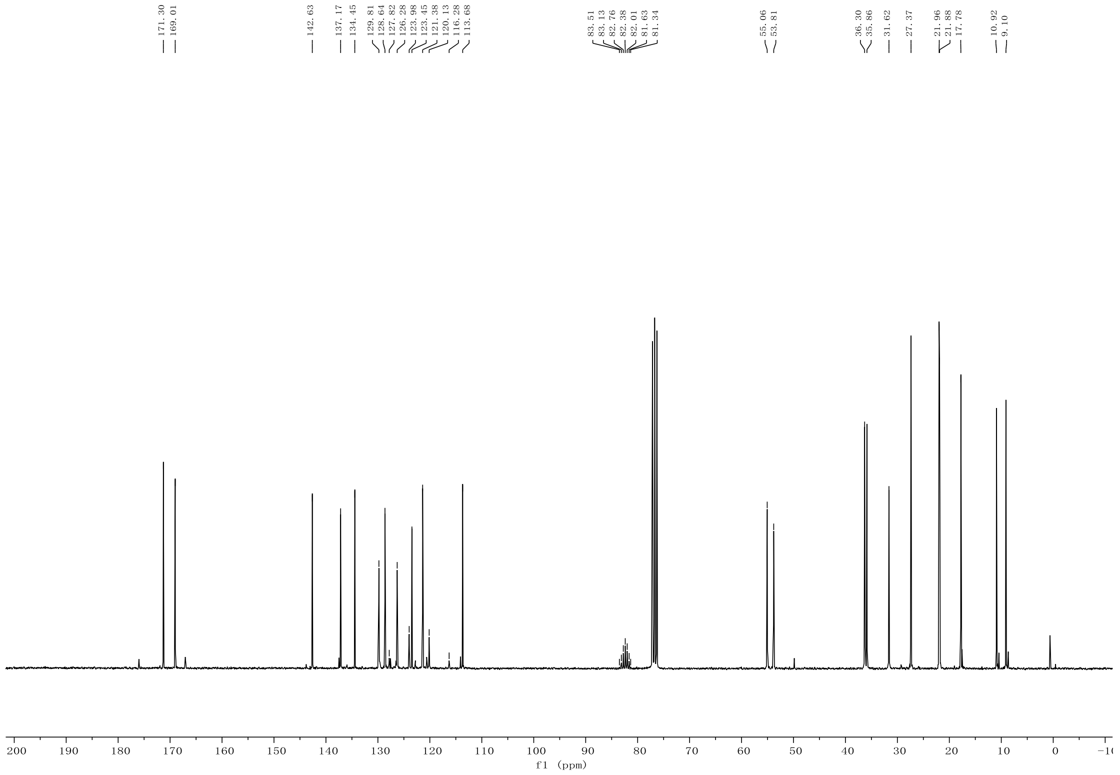

Compound **8i**

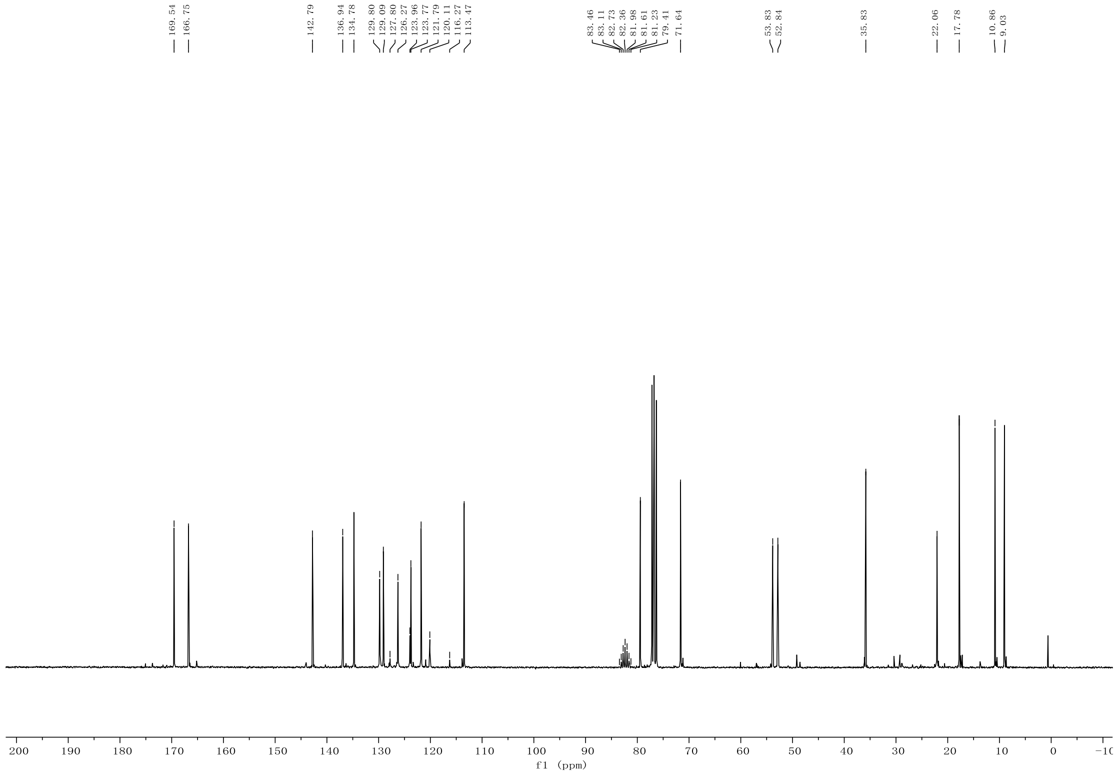

Compound **8j**

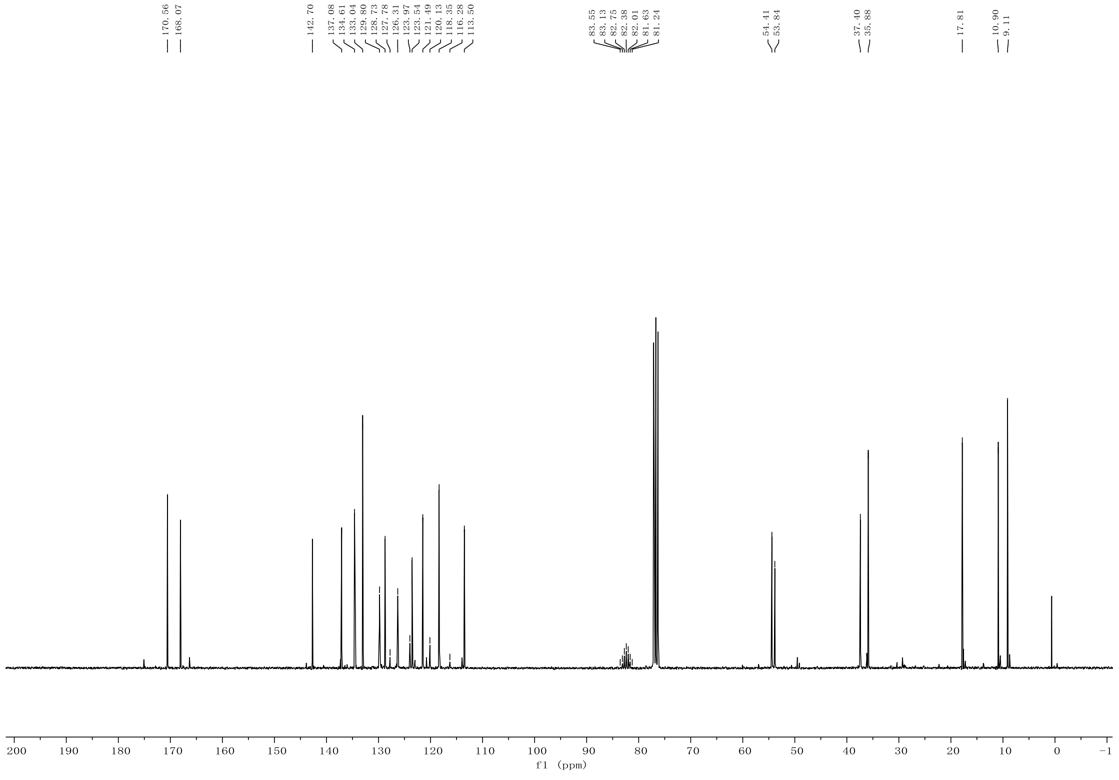

Compound **8k**

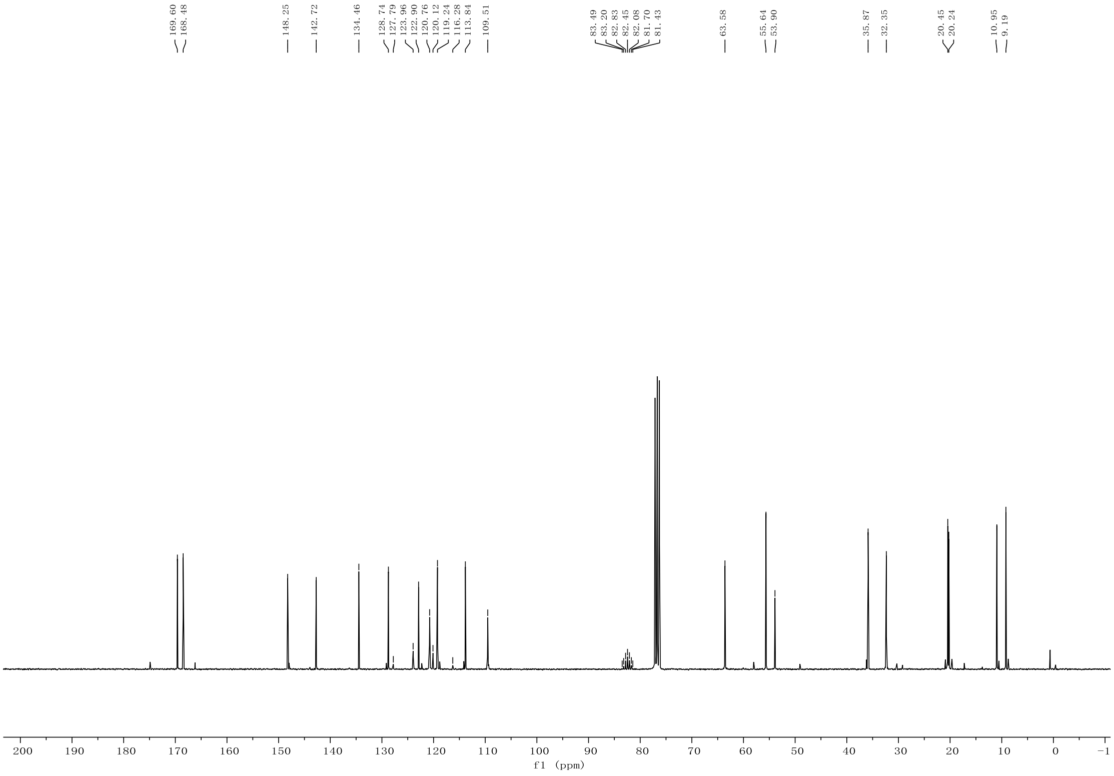

Compound **8l**

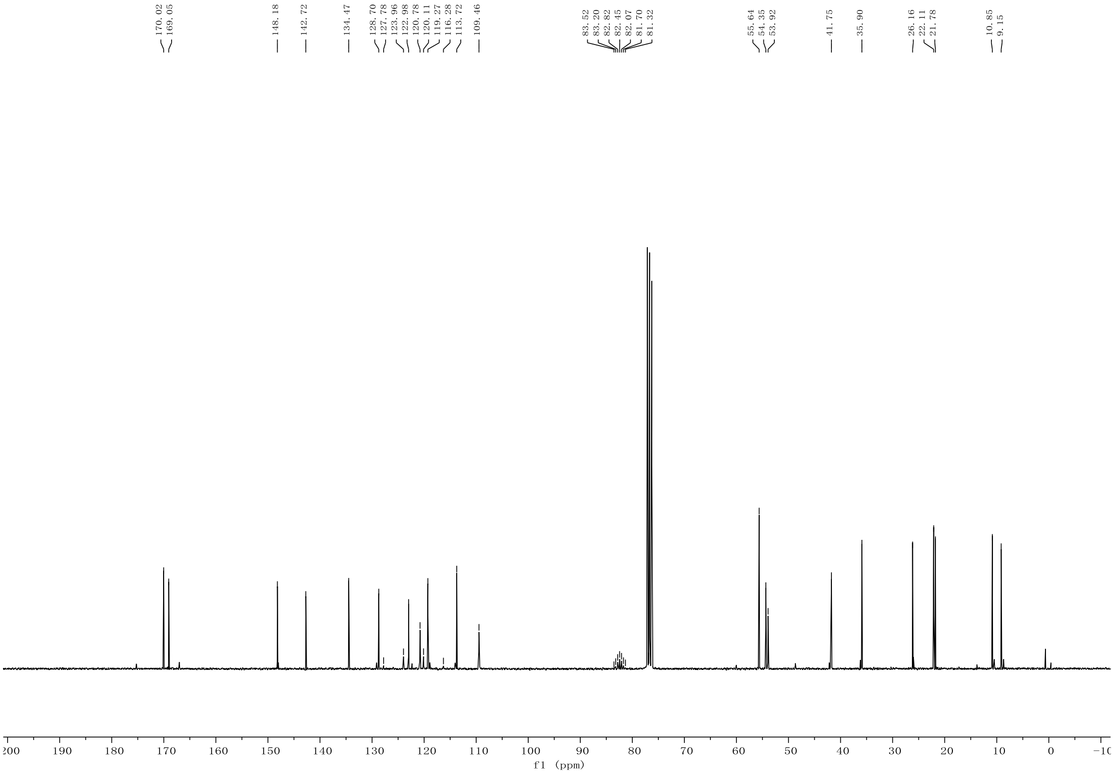

Compound **8m**

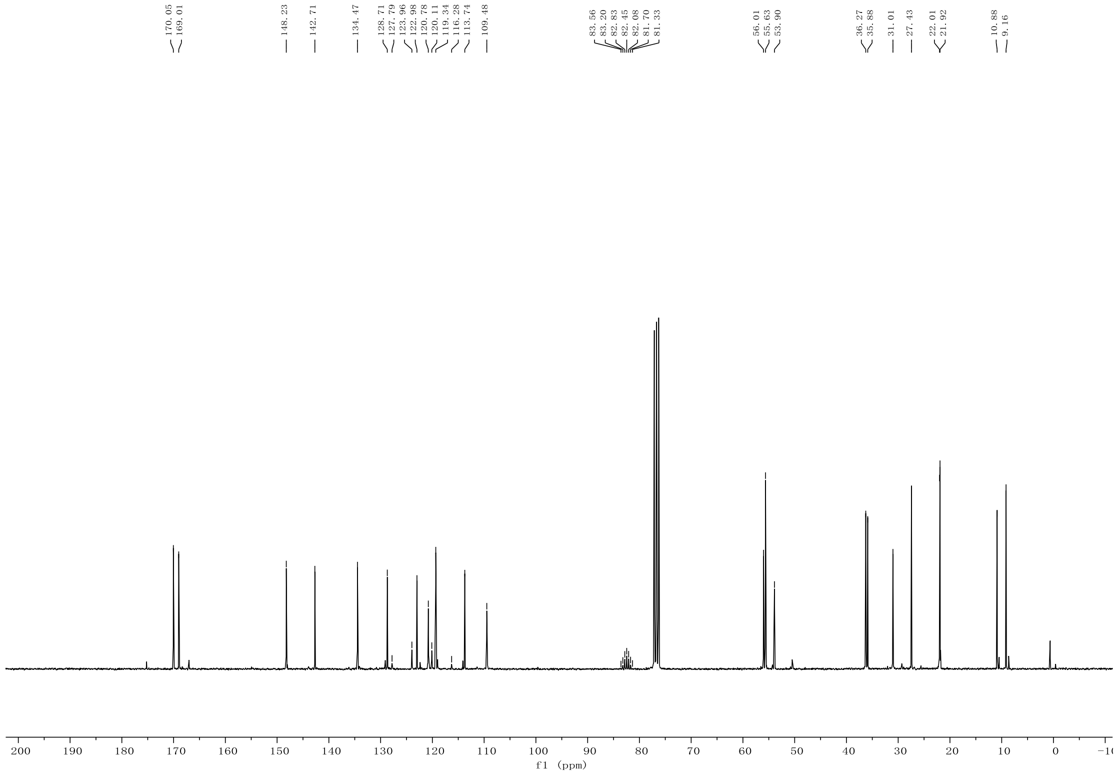

Compound **8n**

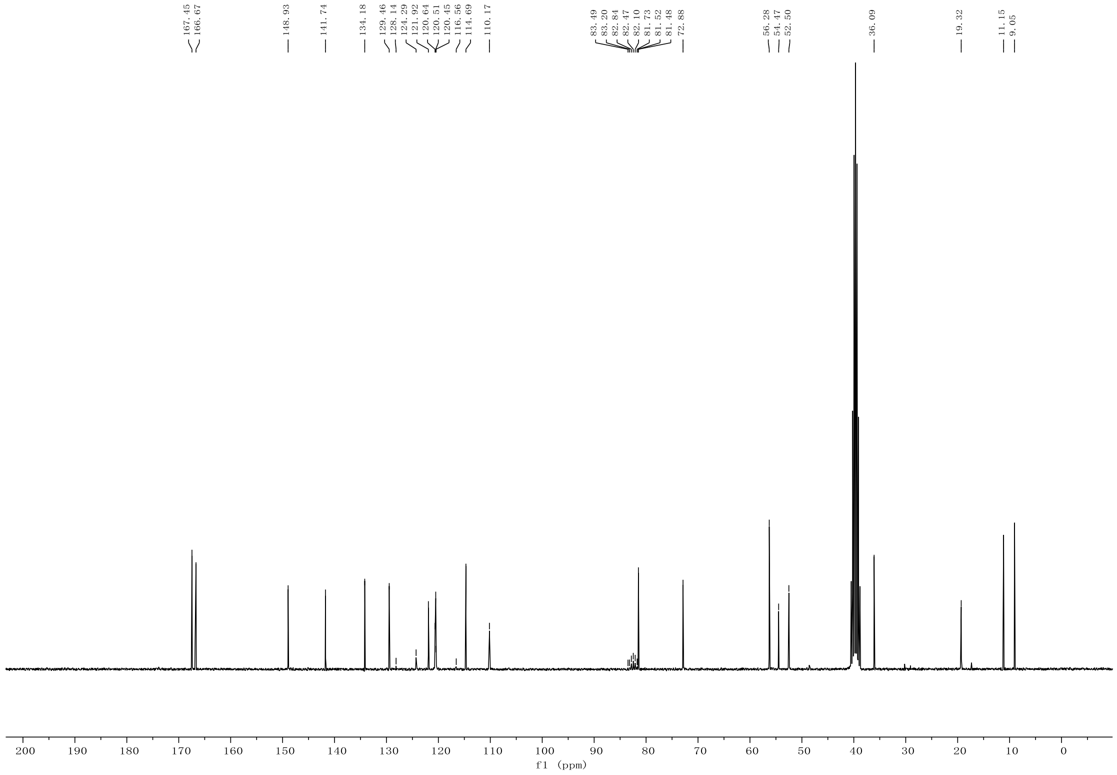

Compound **8o**

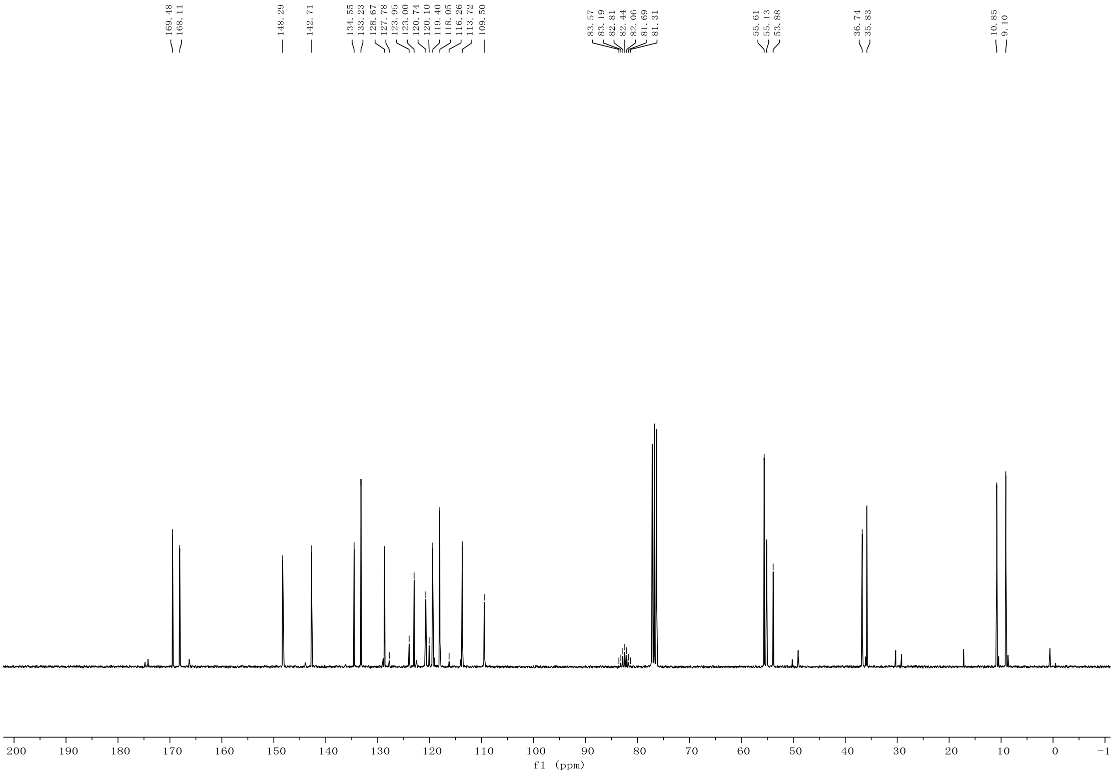

Compound **8p**

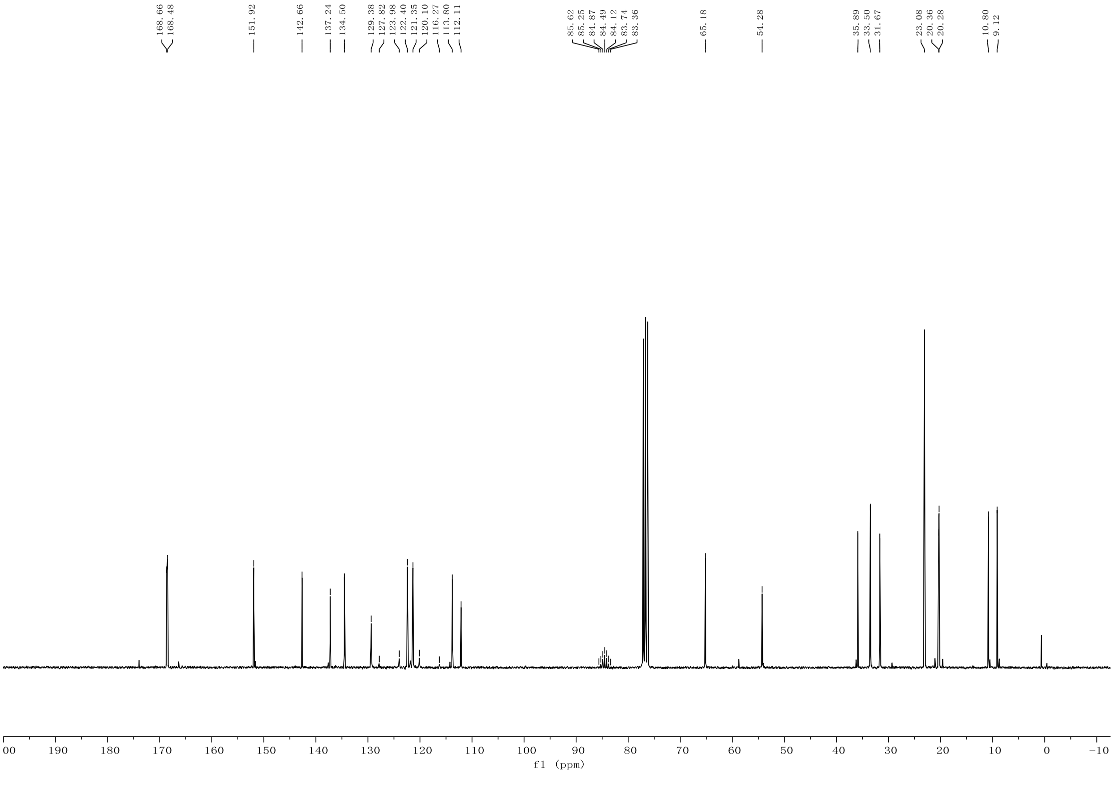

Compound **8q**

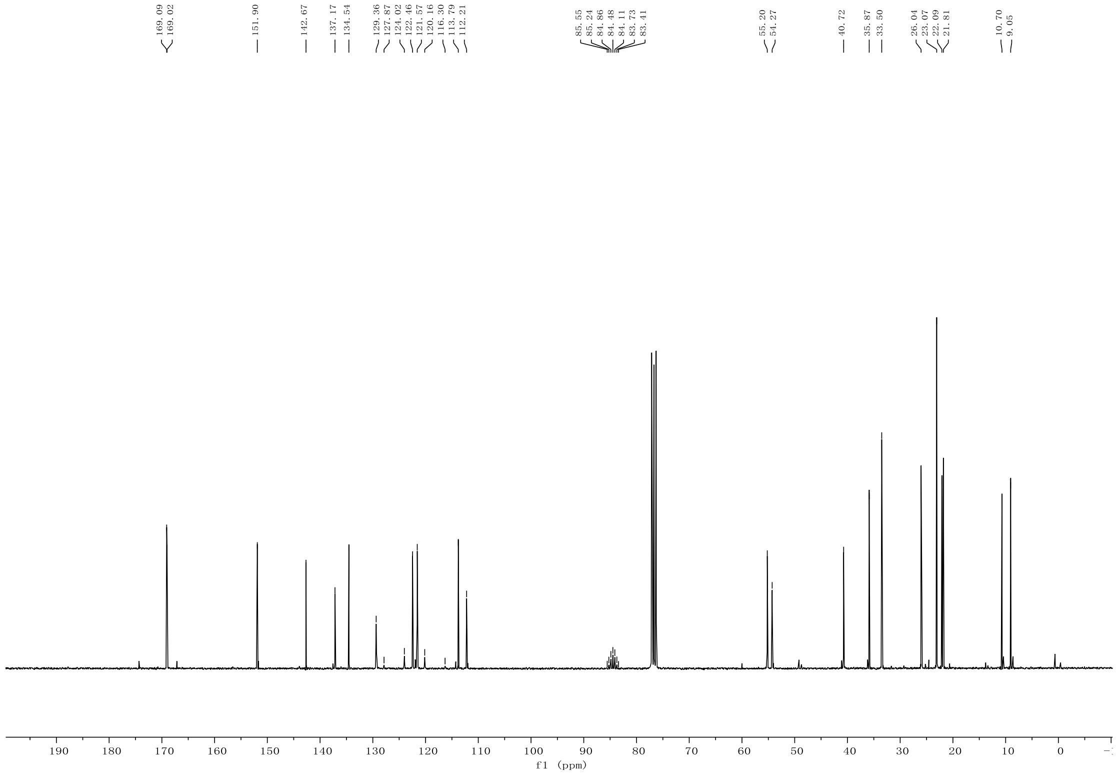

Compound **8r**

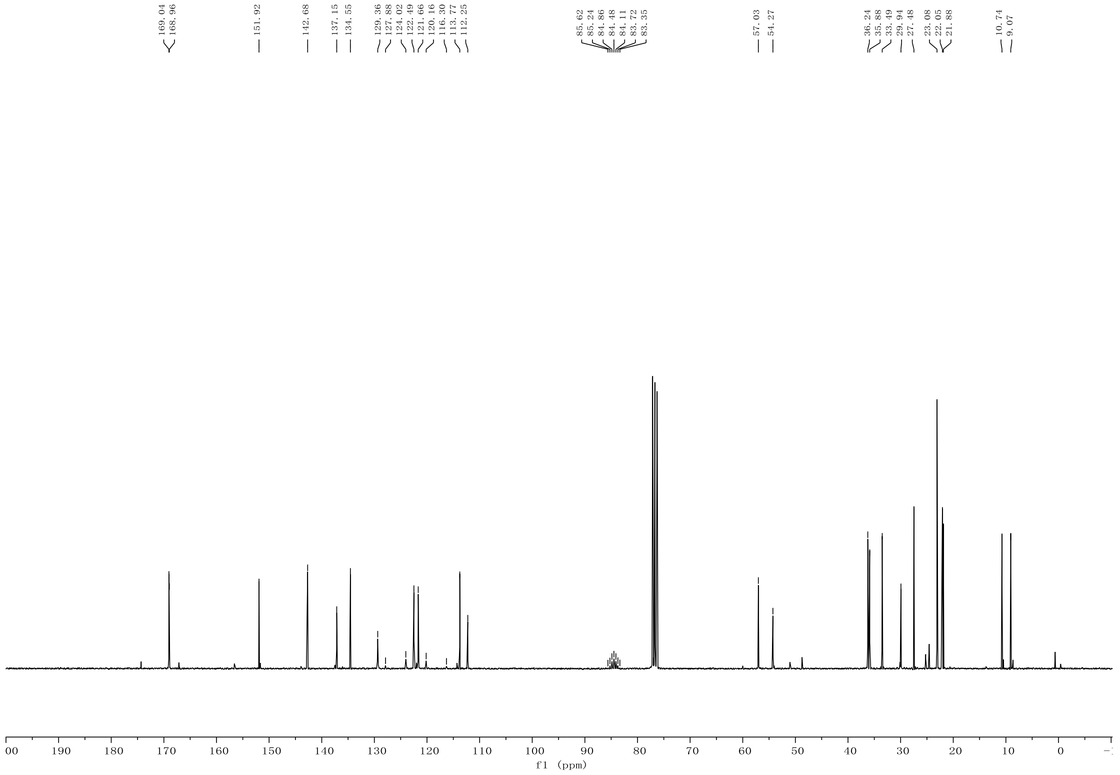

Compound **8s**

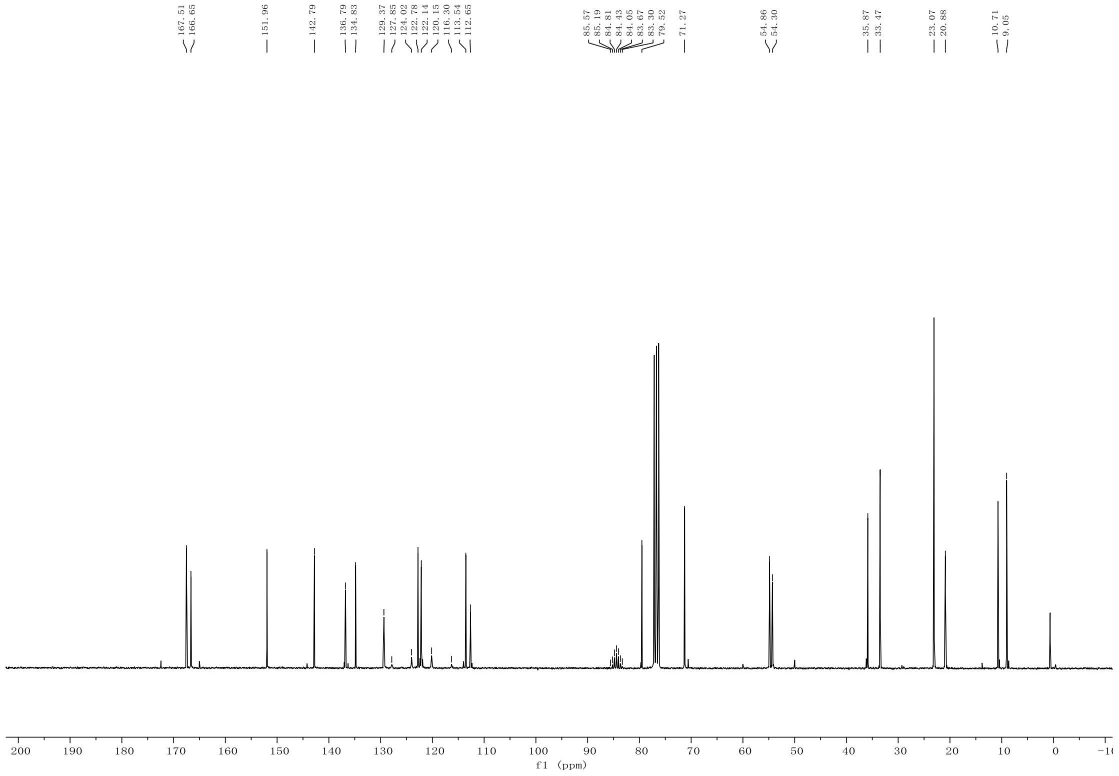

Compound **8t**

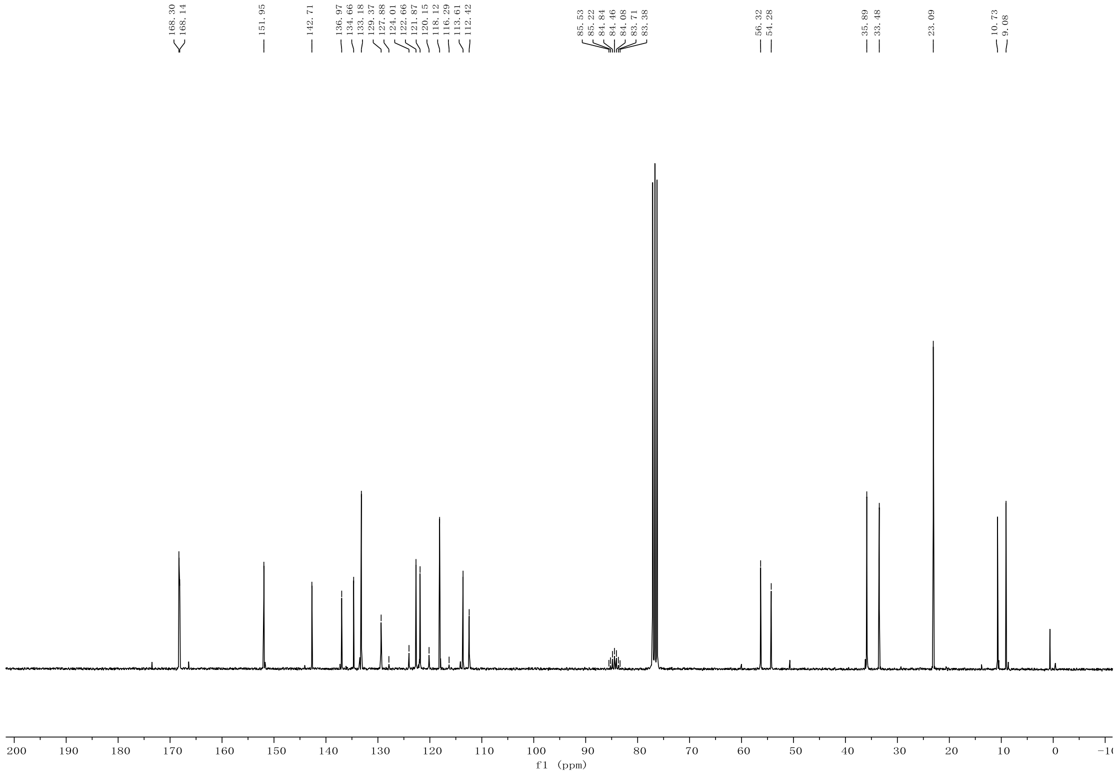

HRMS spectrums of all target compounds

Compound **8a**

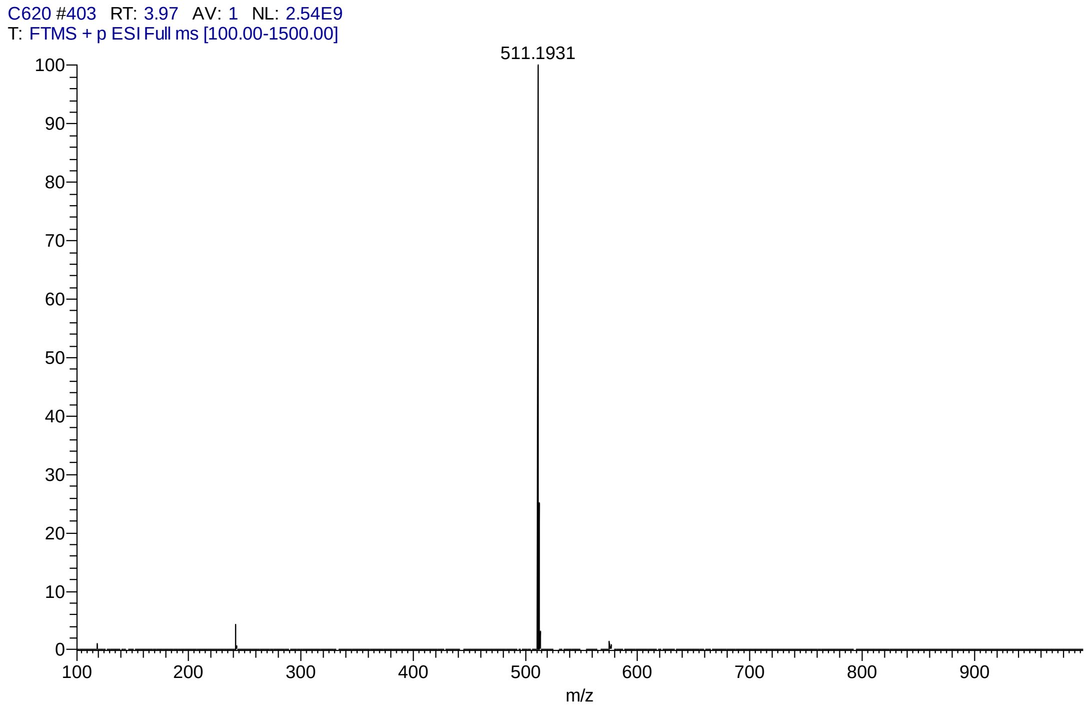

Compound **8b**

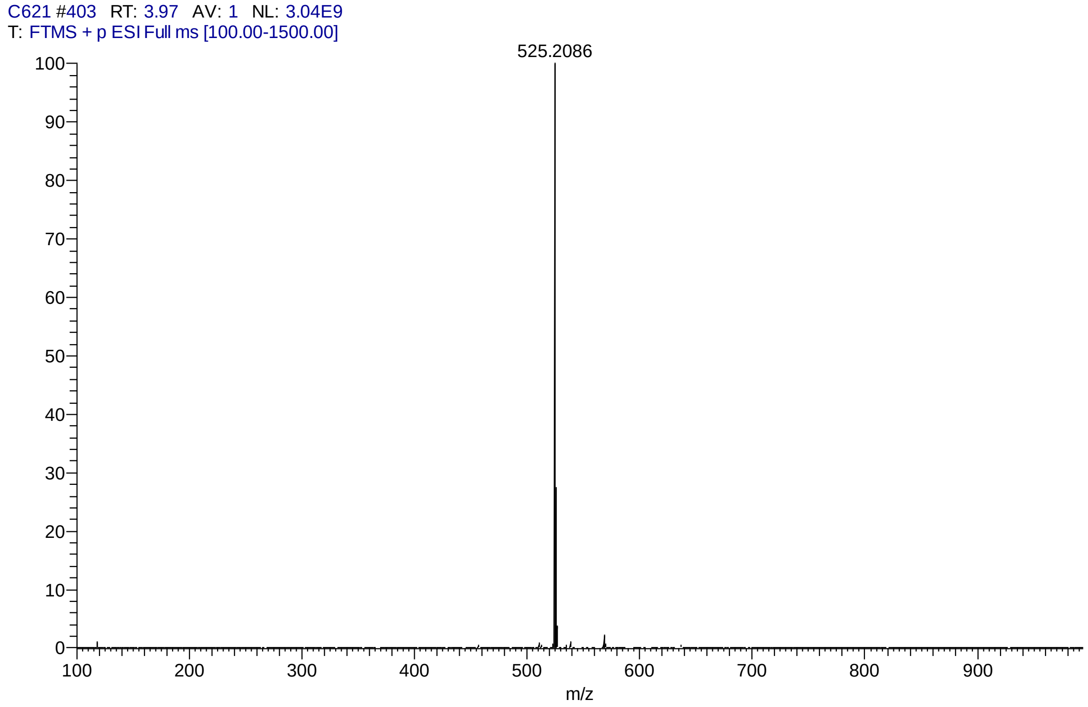

Compound **8c**

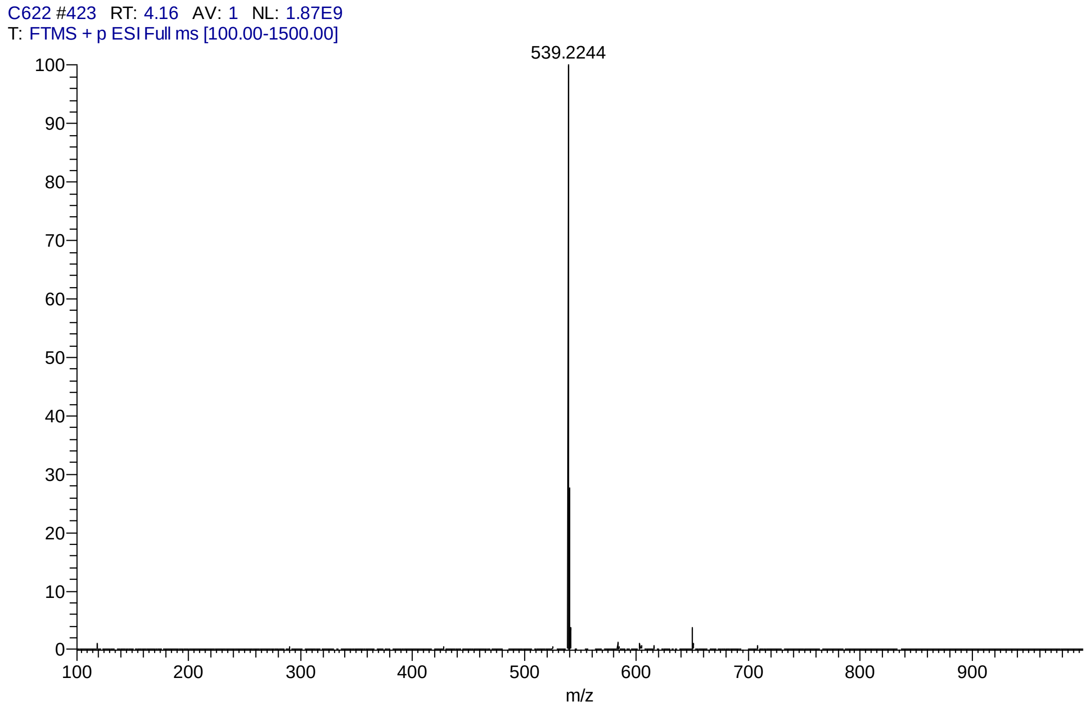

Compound **8d**

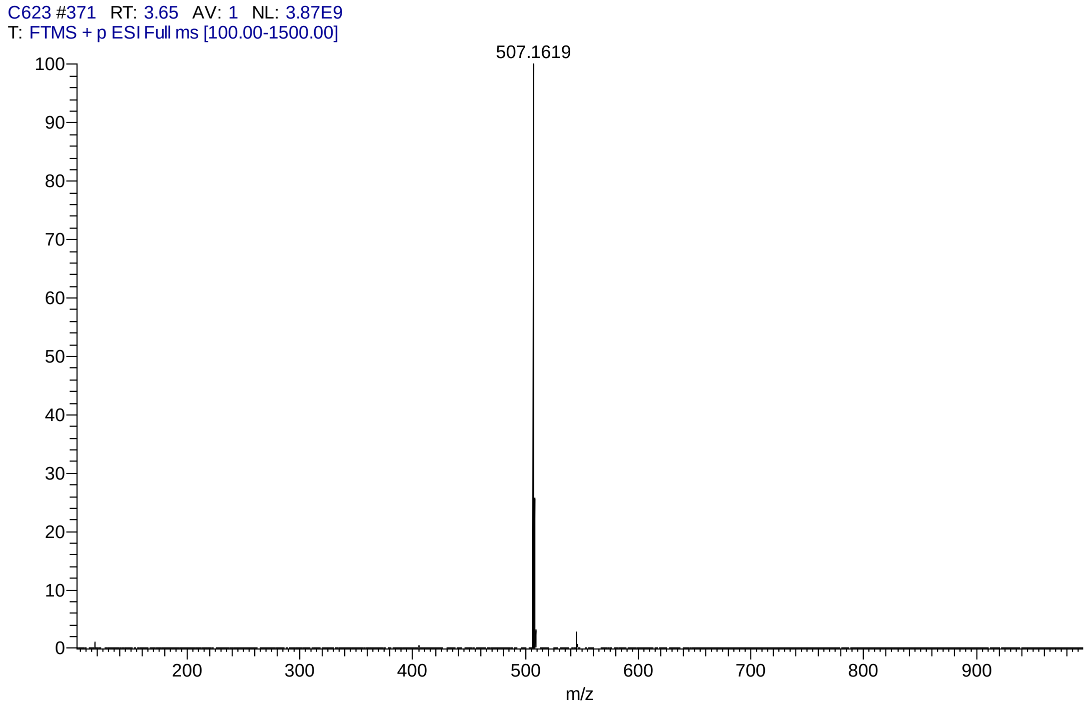

Compound **8e**

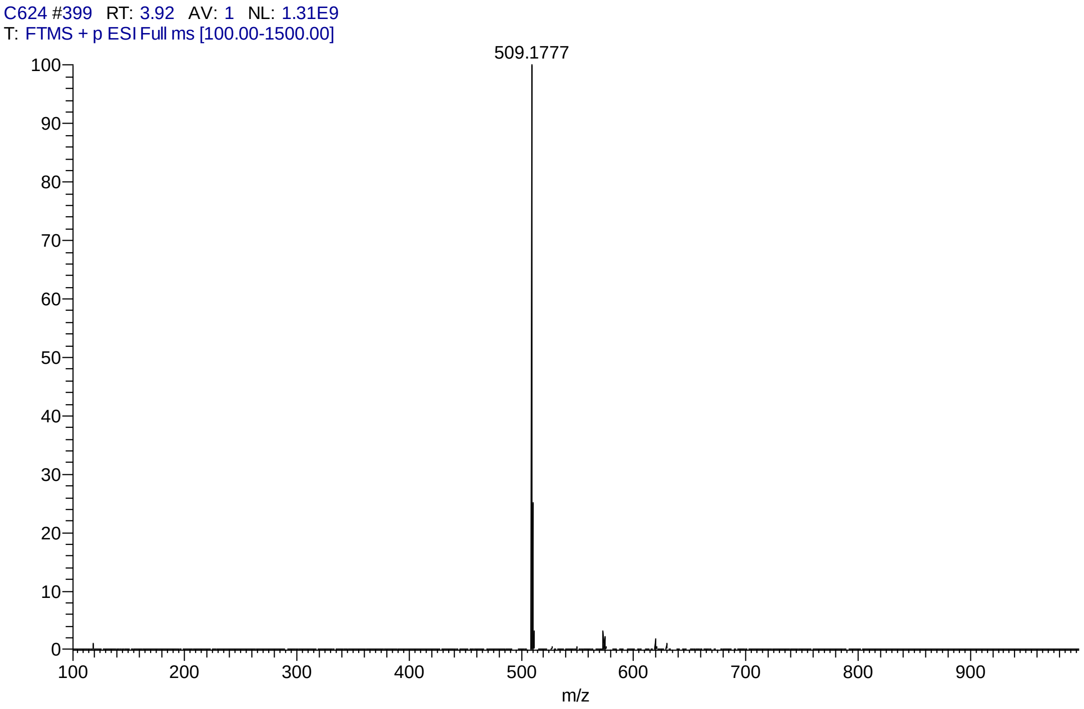

Compound **8f**

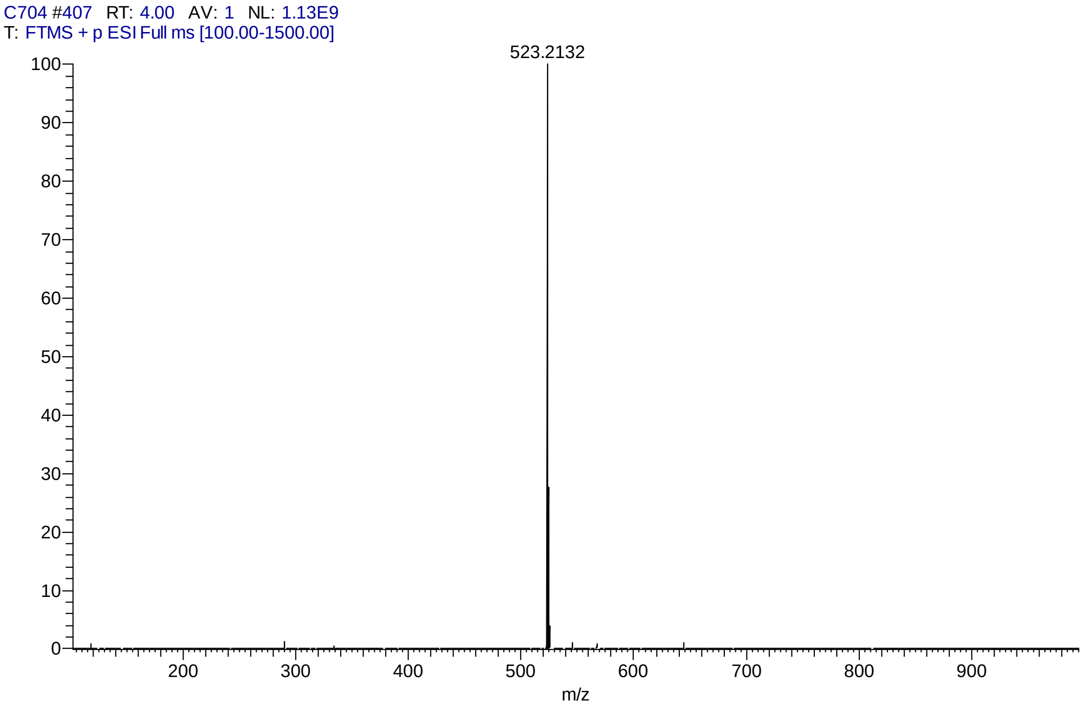

Compound **8g**

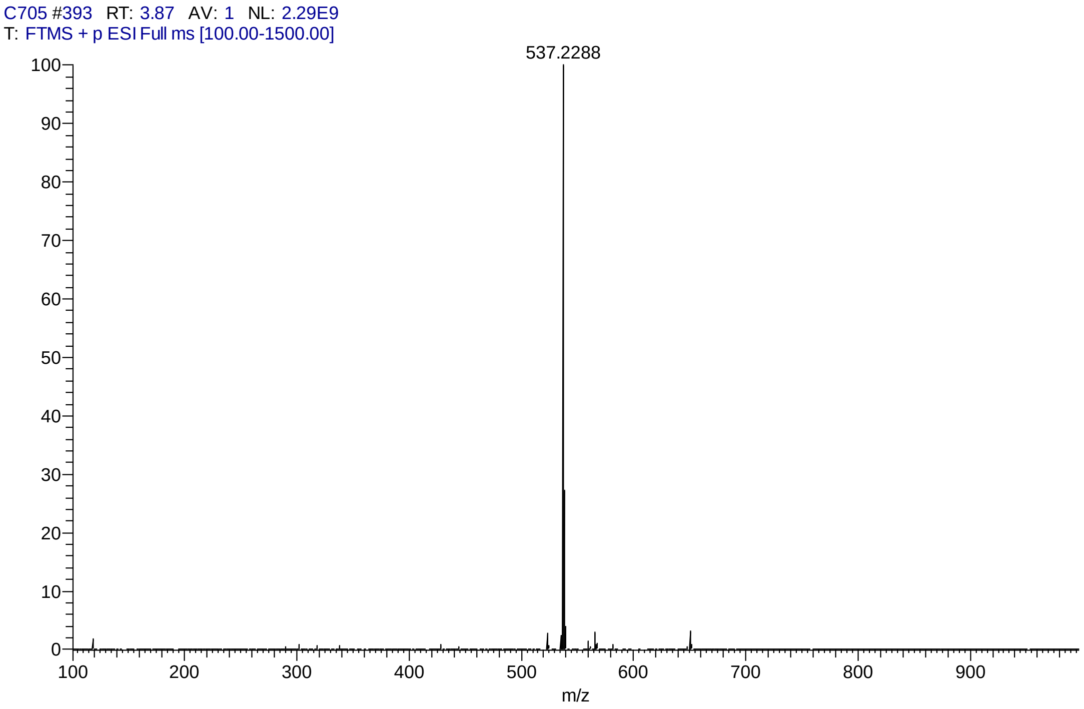

Compound **8h**

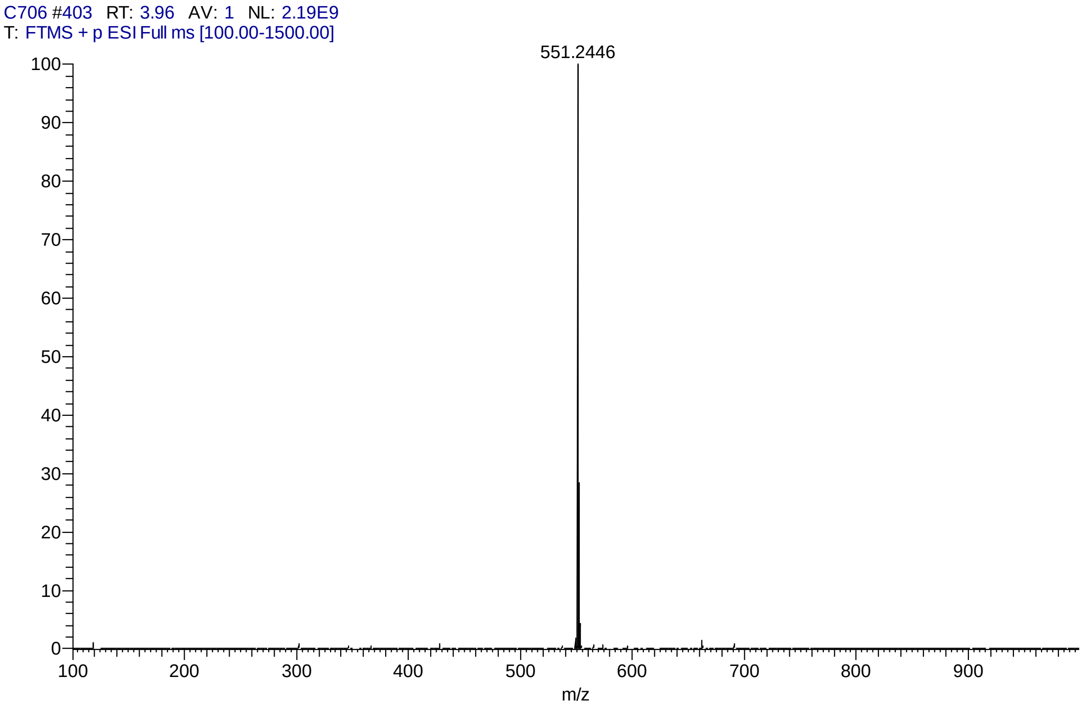

Compound **8i**

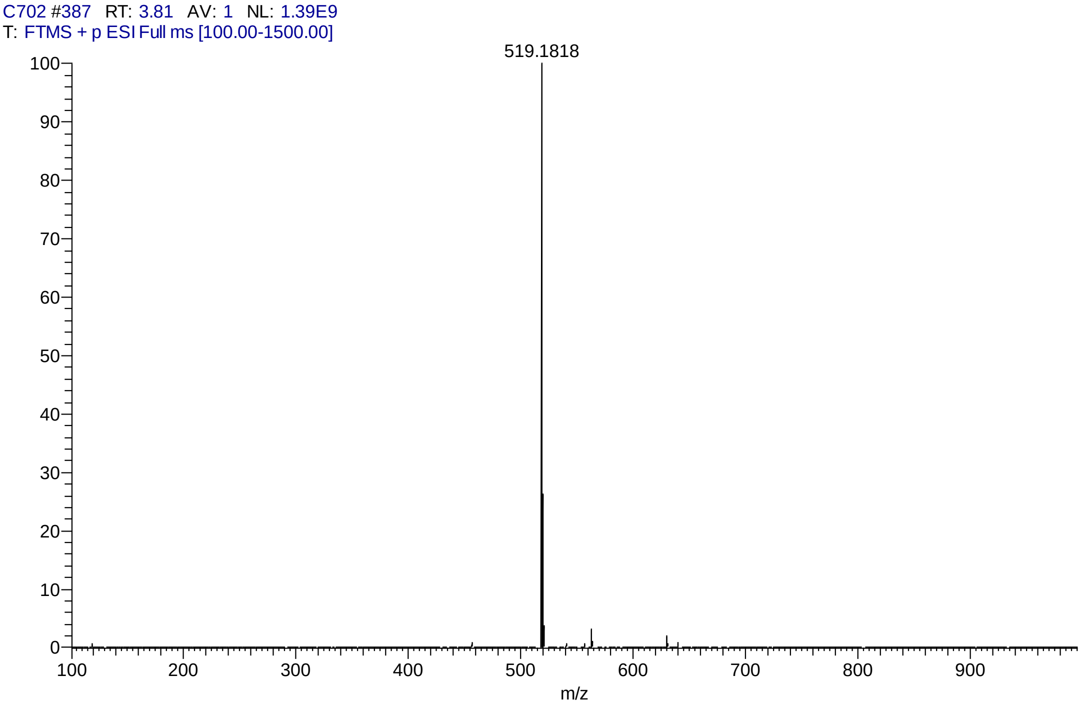

Compound **8j**

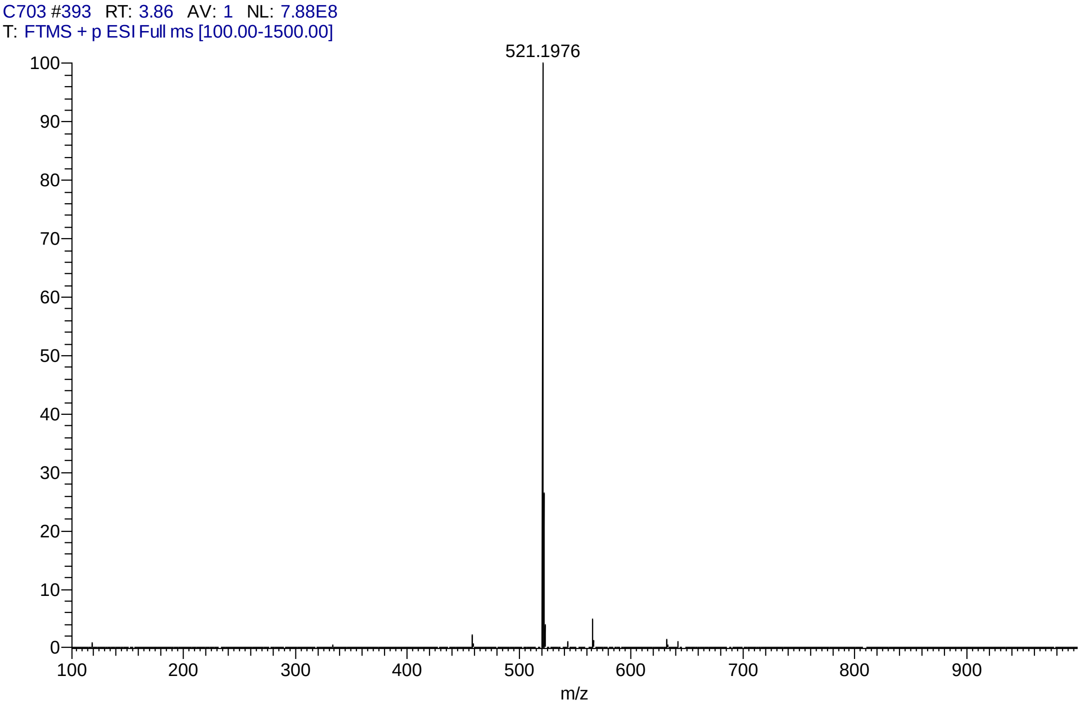

Compound **8k**

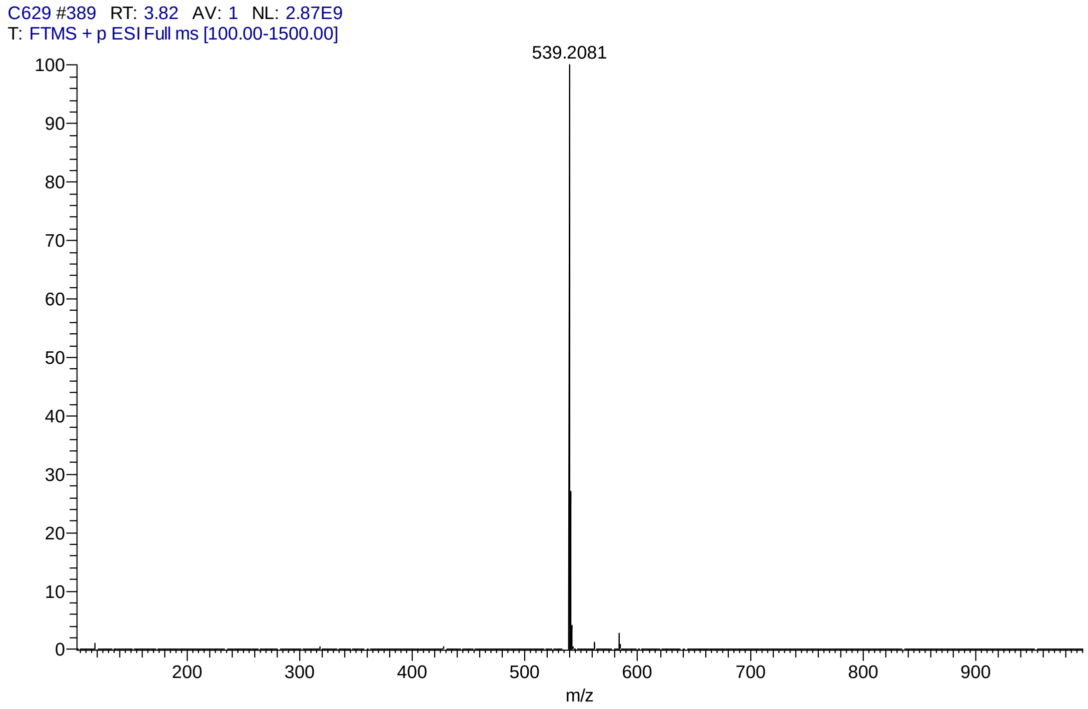

Compound **8l**

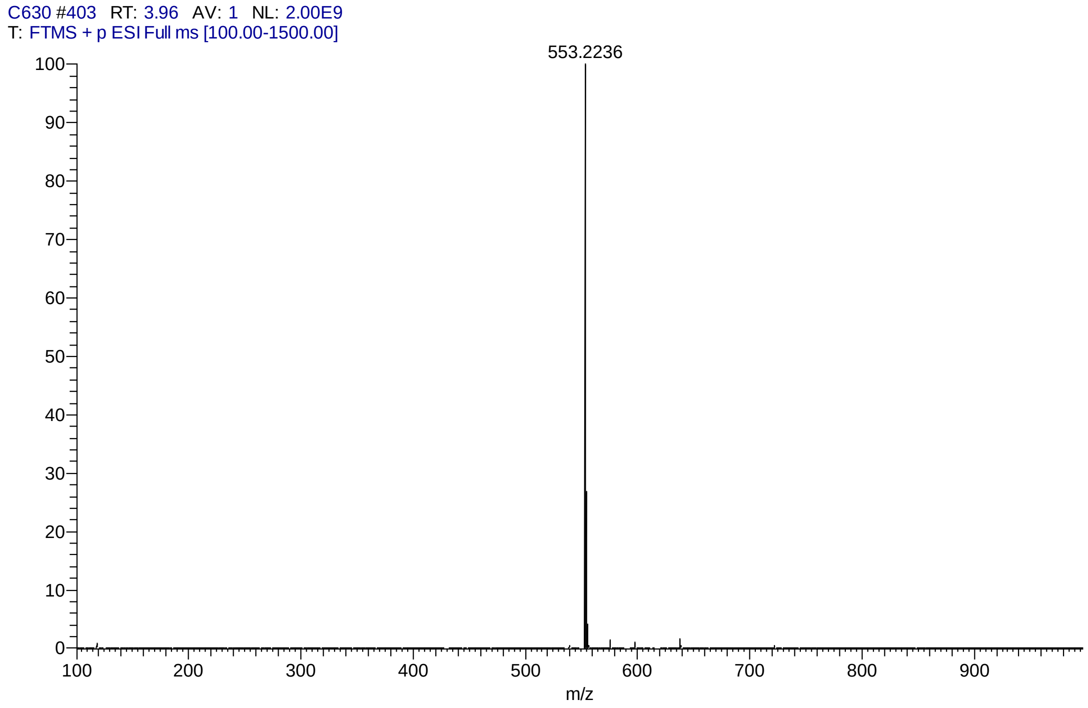

Compound **8m**

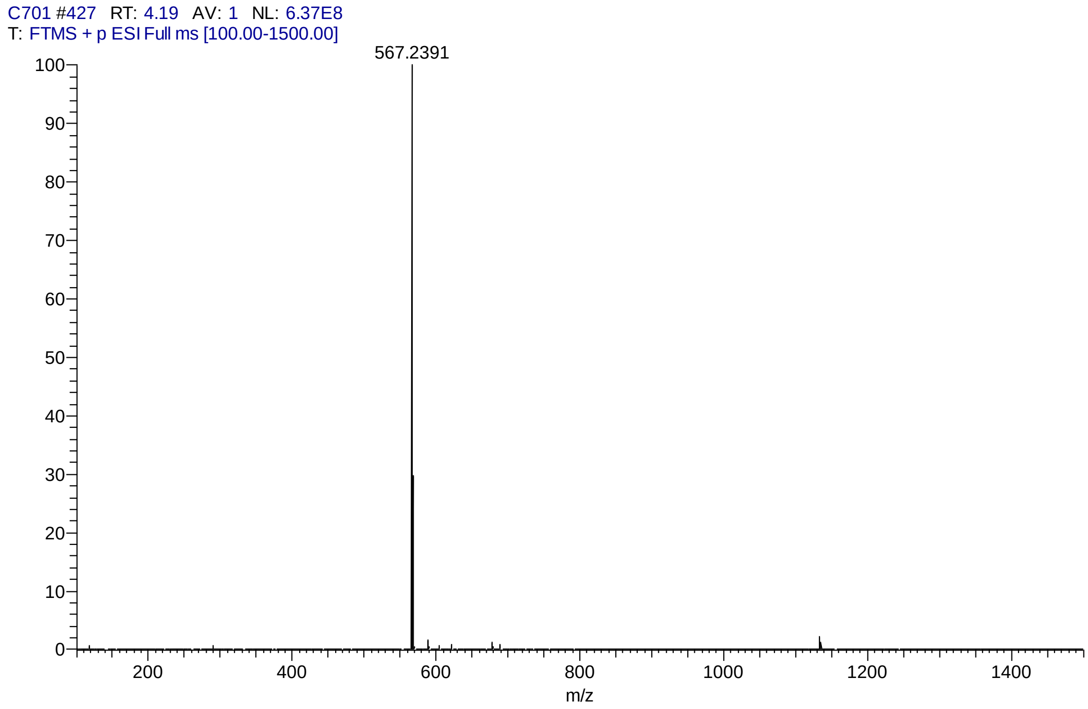

Compound **8n**

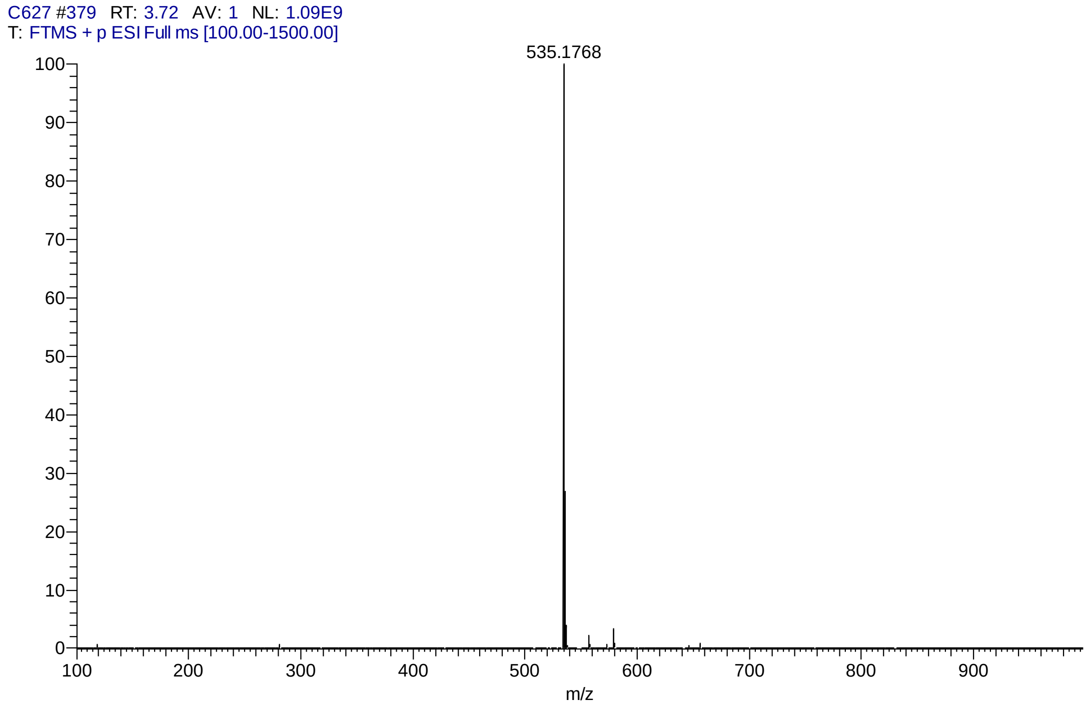

Compound **8o**

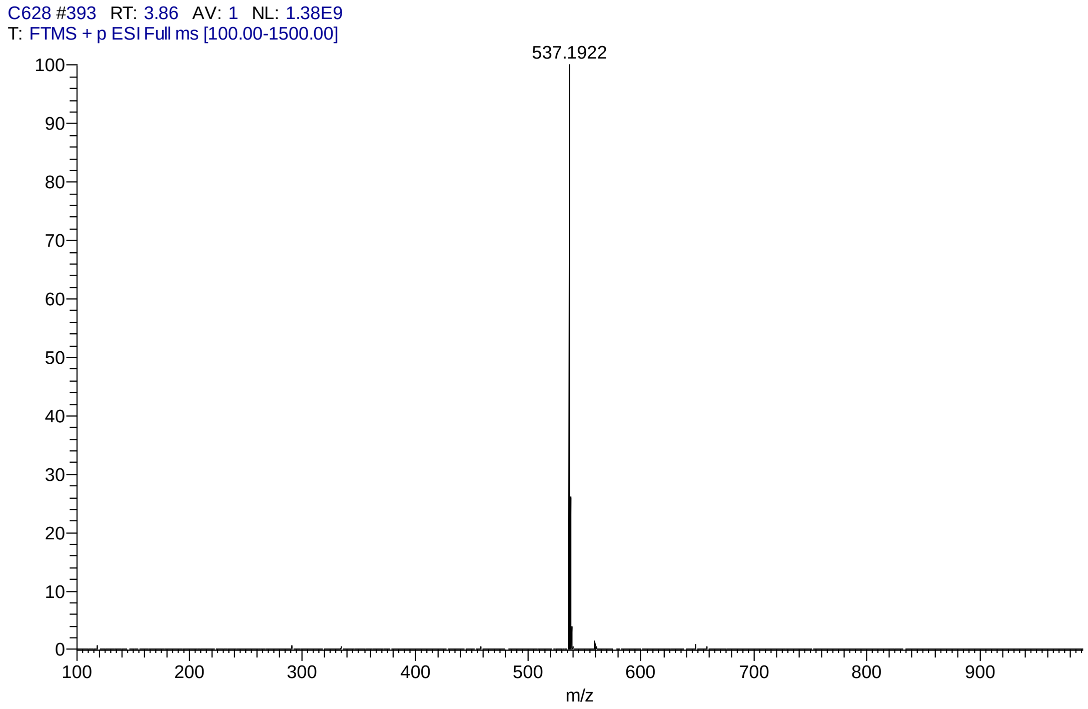

Compound **8p**

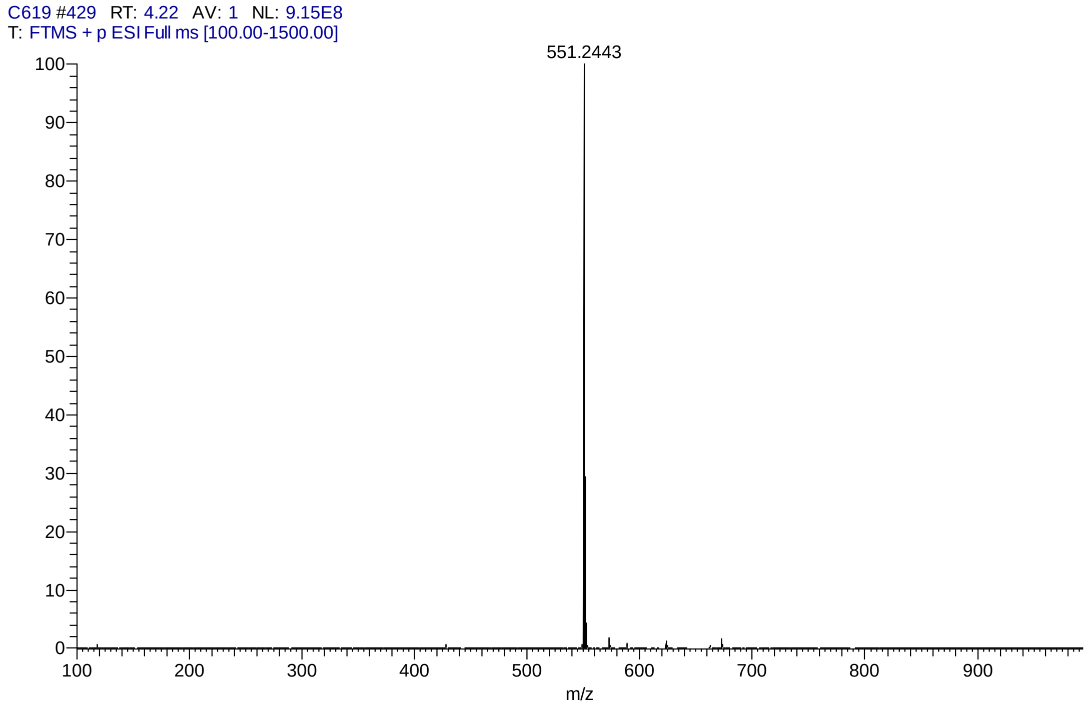

Compound **8q**

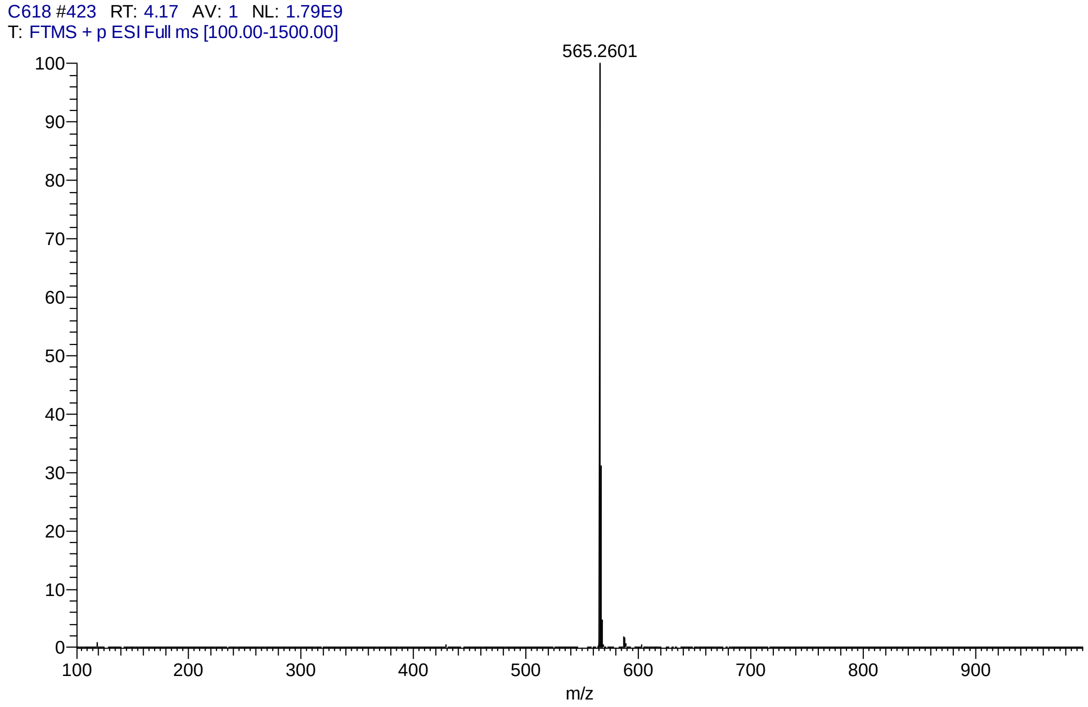

Compound **8r**

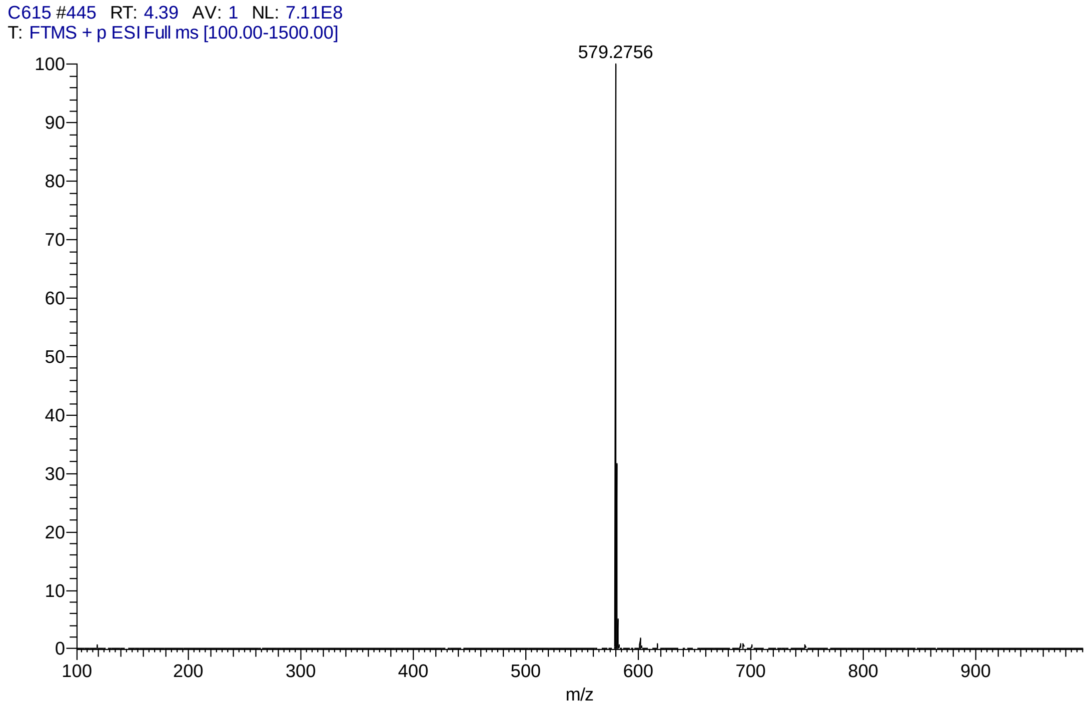

Compound **8s**

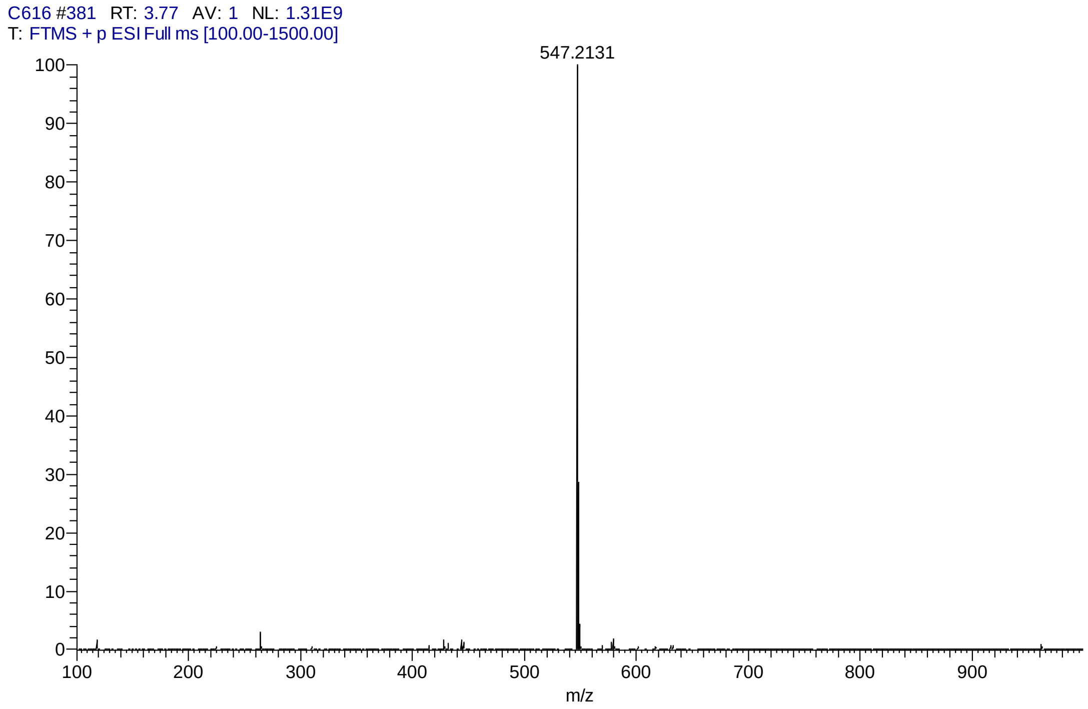

Compound **8t**

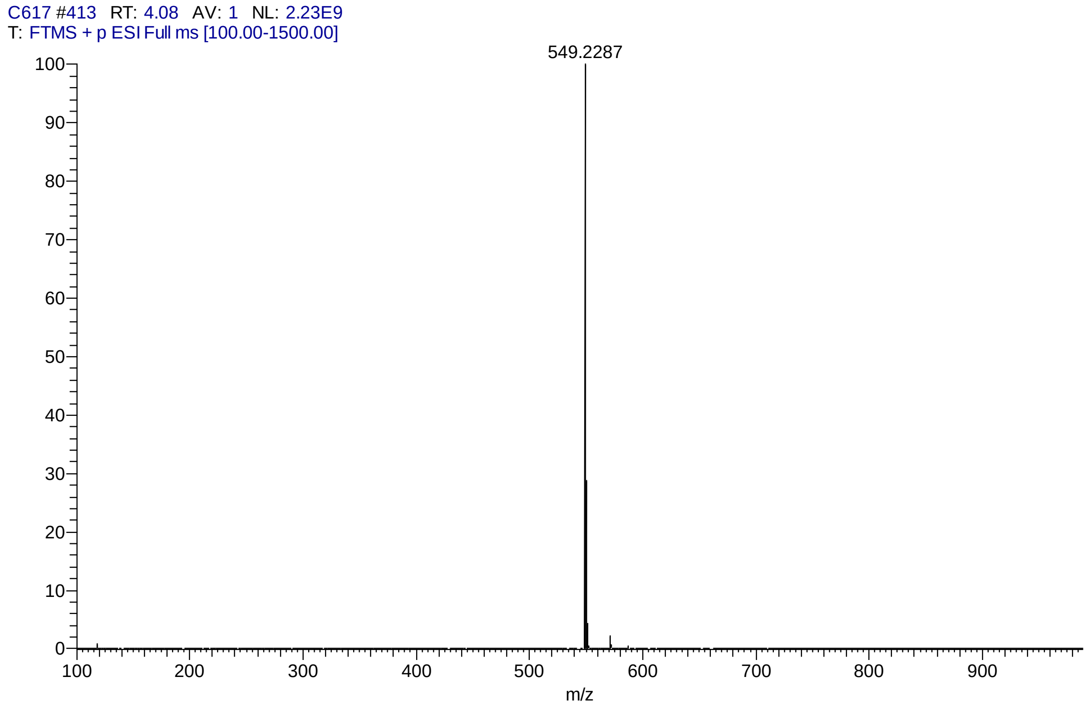

Supplement: Supplementary file 1 [file molecules-24-00562-s001.pdf]
